# Injury-driven stromal exhaustion disrupts intrinsic regenerative capability

**DOI:** 10.1038/s41467-026-76108-z

**Published:** 2026-08-19

**Authors:** Sixun Wu, Hirotaka Matsumoto, Keita Kondo, Aiko Kuroda, Jumpei Morita, Takeshi Moriishi, Shawn A. Hallett, Yuki Matsuo, Azumi Noguchi, Mitsuaki Ono, Makoto Abe, Kosei Ito, Shinsuke Ohba, Noriaki Ono, Yuki Matsushita

**Affiliations:** 1 Department of Skeletal Development and Regenerative Biology, Nagasaki University Graduate School of Biomedical Sciences, Nagasaki, Japan; 2 Leading Medical Research Core Unit, Life Science Innovation, Nagasaki University Graduate School of Biomedical Sciences, Nagasaki, Japan; 3 School of Information and Data Sciences, Nagasaki University, Nagasaki, Japan; 4 Omics AI Research Team, RIKEN Center for Biosystems Dynamics Research, Saitama, Japan; 5 Department of Periodontics and Oral Medicine, University of Michigan School of Dentistry, Ann Arbor, MI USA; 6 Department of Oral Rehabilitation and Regenerative Medicine, Dentistry and Pharmaceutical Sciences, Okayama University Graduate School of Medicine, Okayama, Japan; 7 Department of Tissue and Developmental Biology, Graduate School of Dentistry, The University of Osaka, Osaka, Japan; 8 Department of Molecular Tumor Biology, Nagasaki University Graduate School of Biomedical Sciences, Nagasaki, Japan; 9 University of Texas Health Science Center at Houston School of Dentistry, Houston, TX USA

**Keywords:** Ageing, Mesenchymal stem cells, Regeneration

## Abstract

Bone marrow stromal cells, marked by leptin receptor (Lepr) and C-X-C motif chemokine ligand 12 (Cxcl12), orchestrate osteogenesis and maintain bone homeostasis. Following injury, these stromal cells directly generate reparative bone and subsequently restore the marrow microenvironment, a process widely regarded as a reliable regenerative response. However, it remains unclear whether stromal cells retain full regenerative capacity after prior injury. Here, we show that Lepr^+^Cxcl12^+^ stromal cells have limited regenerative capacity and become dysfunctional upon repeated activation, defining a state of stromal exhaustion. *Lepr-Cre-labeled* stromal cells progressively lose osteogenic potential after repeated injury and instead differentiate into bone marrow adipocytes. Exhausted stromal cells are distinct from aging stromal cells, exhibiting metabolic abnormalities, chronic inflammation associated with stress responses, and impaired regenerative plasticity. Furthermore, β-catenin deletion during injury repair induces adipogenesis through stromal lineage switching, whereas pharmacological and genetic activation of Wnt/β-catenin signaling partially rescues osteogenesis, indicating that dysregulated Wnt/β-catenin signaling drives stromal exhaustion and impaired regeneration. As repeated regenerative activation may occur in recurrent fractures, orthopedic surgeries, osteoporosis, and osteogenesis imperfecta, stromal exhaustion represents a critical barrier to bone regeneration and a potential therapeutic target for restoring stromal plasticity.

## Abstract (web summary)

This study reveals a finite regenerative capacity of Lepr^+^Cxcl12^+^ stromal cells, showing that repeated injury drives stromal exhaustion, adipogenic lineage switching, and impaired bone regeneration via altered Wnt/β-catenin signaling.

## Introduction

As the global population enters a super-aged society, osteoporosis and fractures in the elderly pose serious medical and societal challenges. Fractures frequently result in loss of mobility, causing a bedridden state, and can even lead to increased mortality, making bone repair a critical determinant of a healthy lifespan^1,2^. In the process of bone injury repair, skeletal stem and progenitor cells residing in skeletal tissues play a crucial role. Following injury, these cells are promptly recruited to the injury site and contribute to regeneration through their robust osteogenic potential^3^. It is widely believed that healed bone can regain its original strength and function. Indeed, many clinical guidelines and textbooks explicitly state that the goal of fracture healing is to fully restore the bone to its pre-injury structural and functional state^4^.

However, current clinical and experimental observations raise the possibility that this regenerative capacity may be limited. For example, a large-scale international meta-analysis demonstrated that prior fracture history significantly increases the risk of future fractures, although the underlying mechanisms remain elusive^5^. Thus, the skeleton may not fully regain its regenerative capability following injury. Interestingly, insights from highly regenerative species, such as the axolotl, may offer valuable clues in understanding this phenomenon. Despite their remarkable ability to regenerate entire limbs throughout life, axolotls exhibit a progressive decline in regenerative potential after repeated amputations^6^, pointing to an intrinsic limitation in regenerative cell function over time. These phenomena led us to hypothesize that skeletal cells, which initially exhibit osteogenic potential upon injury, may not indefinitely maintain this ability. Instead, they may enter a state of functional exhaustion, characterized by impaired osteogenic potential and diminished regenerative capacity.

From a biological perspective, periosteal cells and bone-marrow mesenchymal cells coordinate to support bone regeneration by giving rise to osteoblasts and rebuilding skeletal tissue^7–11^. Within the bone marrow environment, leptin receptor (Lepr)- and C-X-C motif chemokine ligand 12 (Cxcl12)-expressing reticular stromal cells in the central marrow space^8,12^ contribute to regeneration, particularly in adulthood, through injury-induced osteogenic differentiation and cellular plasticity^7,8,10,13,14^. Lepr^+^Cxcl12^+^ stromal cells predominantly reside in the perivascular regions of the central marrow cavity^8,15^. During bone growth, these stromal cells are provided by distinct developmental skeletal stem and progenitor cells, including fetal chondrocytes and perichondrial cells^16,17^, early postnatal growth plate stem cells^18,19^, mesenchymal metaphyseal progenitors^20^, and fibroblast growth factor receptor 3 (Fgfr3)-expressing juvenile endosteal stem cells^13^. Lepr^+^Cxcl12^+^ stromal cells are maintained within the central marrow cavity, support the hematopoietic niche^21,22^, and contribute to skeletal homeostasis^8^. A subset of these reticular-shaped stromal cells gradually transitions into adipocytes with aging^8,10^. These stromal cells exhibit a tendency to favor adipogenesis over osteogenesis, thereby increasing susceptibility to age-related osteoporosis^23,24^. Functionally, Lepr and Cxcl12 expressed by reticular stromal cells promote and suppress bone marrow adipogenesis, respectively^25,26^. During injury, these stromal cells exhibit stem cell-like regenerative capacity by differentiating into osteoblasts, temporarily replacing the marrow space with bone, and subsequently reestablishing the stromal and hematopoietic architecture to seemingly restore their original state^10^. However, whether the healed bone marrow environment fully recapitulates its original state and whether Lepr^+^Cxcl12^+^ stromal cells can indefinitely retain their regenerative potential after injury remain unclear.

Here, we demonstrate that repeated activation of bone marrow stromal cells leads to a progressive decline in their regenerative potential, culminating in a distinct state of injury-driven stromal exhaustion marked by loss of osteogenic capacity, a lineage shift toward adipogenesis, and the emergence of chronic inflammatory and metabolic dysregulation. In this context, stromal exhaustion denotes a durable, stress-induced decline in plasticity and regenerative function that is mechanistically and phenotypically distinct from physiological aging. This phenomenon bears resemblance to T cell exhaustion observed in chronic infections, where persistent stimulation leads to a gradual loss of functional potential^27^. Mechanistically, we show that Wnt/β-catenin signaling is essential for preserving stromal identity during regeneration, and its disruption facilitates lineage conversion of stromal cells toward adipogenesis. Conversely, pharmacological or genetic activation of Wnt/β-catenin signaling restores osteogenic regeneration and suppresses adipogenic conversion, supporting its causal role in preventing stromal exhaustion. Our findings establish stromal exhaustion as a central barrier to effective skeletal regeneration and highlight the need to understand its underlying mechanisms for the development of regenerative therapies.

## Results

### Bone marrow stromal cells induce both osteogenic and adipogenic profiles upon injury

To elucidate the transcriptomic response of bone marrow reticular stromal cells to injury, we integrated previously published single-cell RNA sequencing (scRNA-seq) datasets of stromal cell lineages derived from ablated and uninjured control femurs of adult *Cxcl12-creER; R26R*^*tdTomato*^ mice (Supplementary Fig. 1a). The total dataset included 1262 cells from the day 7 injured group and 3231 cells from the control group, which were clustered into 13 distinct clusters (Supplementary Fig. 1b). We further analyzed the expression of stromal, osteogenic, and adipogenic marker genes in the tdTomato^+^ populations (clusters 0, 5, and 11) (Supplementary Fig. 1b). No significant differences were observed in reticular cell markers, such as *Cxcl12*, *Lepr*, *Adipoq*, *Pdgfrb*, *Kitl*, and *Ebf3* indicating that the stability of these cells was maintained in the early phase after injury. In contrast, osteogenic marker genes such as *Alpl*, *Sp7*, *Col1a1*, *Bglap*, *Spp1*, and *Ifitm5* were significantly upregulated in the ablated group (Supplementary Fig. 1c), consistent with our prior findings^10^. This upregulation suggests that stromal cells are actively involved in osteogenesis early in the bone regeneration process. Interestingly, several adipogenesis-related genes, including *Pparg*, were expressed at higher levels in the control group, whereas others, such as *Scd1* and *Rbp4*, were increased in the ablated group. (Supplementary Fig. 1c). These findings suggest that stromal cells maintain their transcriptional identity during the early phase of bone regeneration while initiating both osteogenic and adipogenic differentiation in response to injury.

### Exhausted stromal cells lose osteogenic and proliferative potential

**Fig. 1:**
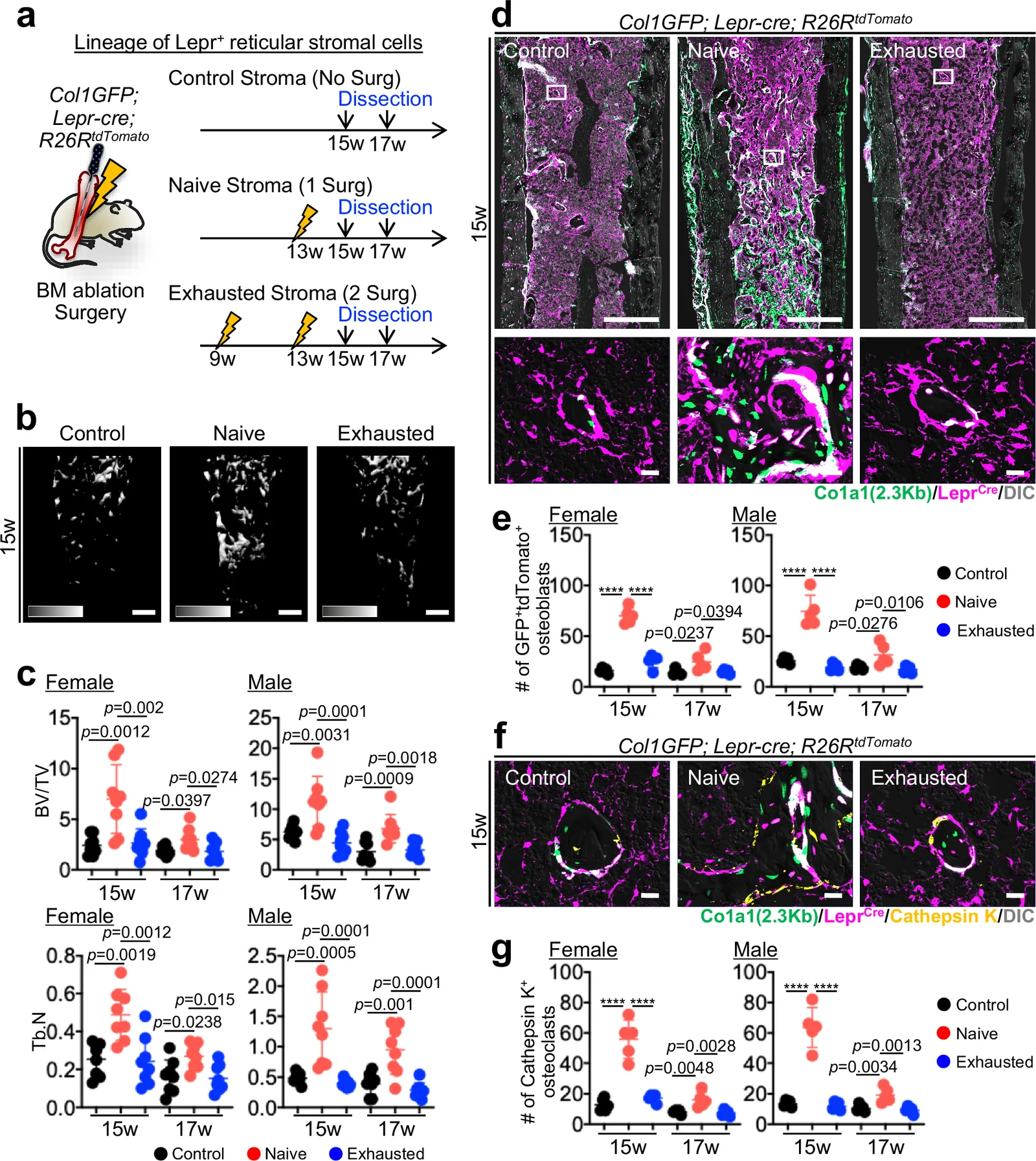
Exhausted Lepr^+^ stromal cells lose osteogenic and proliferative potential. **a** Experimental design illustrating the timeline of bone marrow ablation surgeries and analysis in *Col1a1(2.3 kb)-GFP*; *Lepr-cre*; *R26R*^*tdTomato*^ mice. **b** Representative micro-CT images of distal femurs showing trabecular bone structure in control, naive, and exhausted groups at 15 weeks of age. Scale bar: 500 μm. **c** Quantification of trabecular BV/TV and Tb.N. Control (black), naive (red), and exhausted groups (blue). *n* = 8 mice per group. **d** *Col1a1(2.3 kb)-GFP; Lepr-cre; R26R*^*tdTomato*^ femurs in control, naive, and exhausted groups at 15 weeks of age. Scale bar: upper panel, 500 μm; lower panel, 20 μm. **e** Quantitative analysis of Lepr^Cre^-tomato^+^ osteoblasts at 15 and 17 weeks of age. *n* = 5 mice per group. **f** Immunofluorescence staining for Cathepsin K from *Col1GFP; Lepr-cre; R26R*^*tdTomato*^ mice at 15 weeks. Scale bar: 20 μm. **g** Quantification of Cathepsin K^+^ osteoclasts. *n* = 5 mice per group. Exact P values are shown in the graphs; ****p < 0.0001. Two-tailed, One-way ANOVA followed by Tukey’s post hoc test. Data are presented as mean ± S.D. Source data are provided as a Source Data file.

To investigate the dynamics of injury-driven stromal exhaustion —a durable, stress-induced decline in the plasticity and regenerative function of bone marrow stromal cells, typically accompanied by an adipogenic lineage shift— we first established a mechanical stimulation model by performing bone marrow ablation surgery on the right femur^10,28^. We traced the fates of Lepr^+^ reticular stromal cells in response to mechanical injury. We utilized *Col1a1(2.3 kb)-GFP; Lepr-cre; R26R*^*tdTomato*^ mice, stratified into three experimental groups: control (no surgery), naive (single surgery at 13 weeks of age), and exhausted (repeated surgery at 9 and 13 weeks). Analyses were performed at 15 and 17 weeks, corresponding to 2- and 4-weeks post-surgery, respectively (Fig. 1a). Notably, the bone marrow appeared to have returned to a baseline state that closely resembled the uninjured marrow at approximately 4 weeks after the first surgery, indicating that the marrow environment at the time of the second surgery was histologically comparable to uninjured control (Supplementary Fig. 2a, b).

First, we examined the bone regenerative potential of Lepr^+^ stromal cells in each state. Micro-CT analysis revealed that the distal femur in the naive group had a greater trabecular bone area than that in the exhausted group (Fig. 1b). Quantitative analysis further showed that bone volume/tissue volume (BV/TV) and trabecular number (Tb.N) were significantly higher in the naive group than in the exhausted group, when compared to their respective contralateral (control) femurs, regardless of sex (Fig. 1c). Serial in vivo micro-CT analysis including baseline time points further confirmed that trabecular bone in the analyzed region was robustly regenerated after the first ablation, whereas this regenerative response was markedly impaired after the second ablation (Supplementary Fig. 3a–c). Histological analysis confirmed that bone marrow ablation induced the differentiation of Lepr^Cre^-tdTomato^+^ cells into Col1a1(2.3 kb)-GFP^+^ osteoblasts within the regenerative bone of the naive group (Fig. 1d). Interestingly, in the exhausted group, little to no bone regeneration was observed, and Lepr⁺ stromal cells failed to differentiate into osteoblasts. At both time points, the number of Col1a1(2.3 kb)-GFP^+^Lepr^Cre^-tdTomato^+^ osteoblasts in the naive group remained significantly higher than in the exhausted group, despite a reduction in osteogenesis from 15 weeks to 17 weeks (Fig. 1e). These findings indicate that stromal cell exhaustion results in a diminished capacity for osteoblast differentiation and bone formation.

Next, to verify whether repeated injury significantly impairs the proliferative capacity of Lepr⁺ stromal cells, we performed ex vivo colony-forming unit-fibroblast (CFU-F) assays of bone marrow cells isolated from each group and cultured at clonal density (Supplementary Fig. 4a). Both the number and size of Lepr^Cre^-tdTomato^+^ colonies as well as the total CFU-Fs, were significantly higher in the naive versus exhausted group (Supplementary Fig. 4b). Notably, the exhausted group exhibited fewer colonies than the control group, indicating impaired proliferative and colony-forming capacity (Supplementary Fig. 4b). These results suggest that repeated injury impairs the ability of stromal cells to sustain progenitor populations and contribute effectively to tissue repair.

Additionally, in the naive group, elevated numbers of osteoclasts indicated enhanced bone resorption in response to injury (Fig. 1f, g), consistent with a high bone turnover state. Importantly, it is considered that bone formation was more active than resorption, contributing to an increase in bone mass. In contrast, the exhausted group exhibited markedly impaired osteogenic capacity, suggesting a low turnover state characterized by reduced bone formation and limited regenerative potential.

Collectively, our findings demonstrate that repeated injury induces exhaustion in marrow reticular stromal cells, leading to compromised proliferation and osteogenesis. This stromal exhaustion results in reduced bone formation capacity, which contributes to impaired bone regeneration and increased susceptibility to bone loss.

### Injury-driven exhausted stromal cells promote adipogenic differentiation

**Fig. 2:**
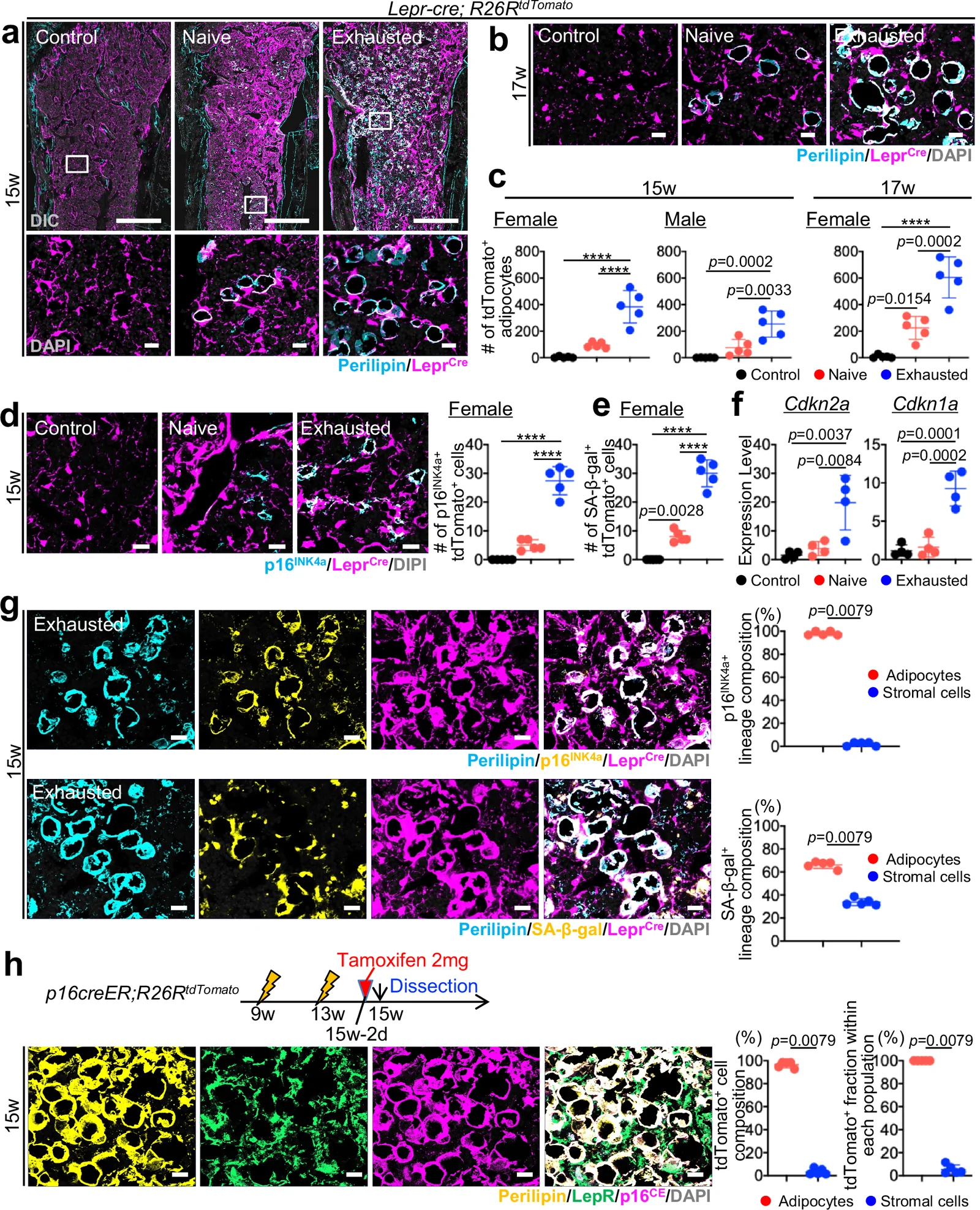
Injury-driven exhausted stromal cells promote adipogenic differentiation. Representative immunofluorescence images and quantification of Perilipin^+^ adipocytes in femurs from *Lepr-cre; R26R*^*tdTomato*^ mice in control, naive, and exhausted groups at 15 weeks (**a, c**) and 17 weeks (**b, c**) of age. Scale bar: upper panel in (**a**), 500 μm; lower panel in (**a**) and **b**, 20 μm. *n* = 5 mice per group. **d** Representative images and quantification of p16^INK4a+^tdTomato^+^ cells at 15 weeks. Scale bars, 20 μm. *n* = 5 mice per group. **e** Quantification of SA-β-gal^+^tdTomato^+^ cells at 15 weeks. *n* = 5 mice per group. **f** Quantitative RT-PCR analysis of FACS-sorted CD45^-^Ter119^-^CD31^-^ bone marrow mesenchymal cells for *Cdkn2a* and *Cdkn1a*. *n* = 4 mice per group. **g** Representative fluorescence images and quantification of p16^INK4a^ and SA-β-gal signals in Perilipin^+^ adipocytes and Lepr^+^ stromal cells. Scale bars, 20 μm. *n* = 5 mice per group. **h** *p16-creER*-labeled cells in the adipocyte and stromal cell fractions. Bone marrow ablation surgeries were performed at 9 and 13 weeks of age, and femurs were dissected at 15 weeks (tamoxifen was injected 2 days before dissection). Scale bars, 20 μm. *n* = 5 mice per group. Exact *P* values are shown in the graphs; ****p < 0.0001. Two-tailed, One-way ANOVA followed by Tukey’s post hoc test for (**c–f**). Two-tailed, Mann-Whitney U test for **(g, h**). Data are presented as mean ± S.D. Source data are provided as a Source Data file.

In the bone marrow, osteogenesis and adipogenesis are generally regarded as reciprocal processes^29,30^. To investigate whether exhausted stromal cells exhibit alterations in adipogenic differentiation, we conducted histological assessments of femurs from *Lepr-cre; R26R*^*tdTomato*^ mice using immunofluorescence staining with the adipocyte marker Perilipin^8^. Our data revealed a significant increase in Lepr^Cre^-tdTomato^+^ adipocytes in the exhausted group two weeks post-surgery (Fig. 2a, c). Four weeks post-surgery, adipocytes in the exhausted group were densely distributed throughout the marrow cavity (Fig. 2b), further confirming the enhanced adipogenic differentiation associated with bone marrow fat accumulation observed in exhausted stromal cells (Fig. 2c and Supplementary Fig. 5a). Moreover, consistent with our scRNA-seq results, the number of adipocytes in the naive group following injury also increased compared to controls (Supplementary Fig. 1). Notably, female mice in the exhausted group exhibited a higher number of adipocytes compared to male mice, which may be attributed to estrogen’s role in promoting imbalances in fat-bone distribution^31^. These results suggest that Lepr^+^ bone marrow stromal cells, especially in the exhausted state, contribute to marrow adipocytes after bone marrow injury.

Cellular senescence refers to the process by which cells lose their ability to divide and regenerate, effectively contributing to aging^32^. To assess whether exhausted stromal cells become senescent, we performed immunofluorescence staining for the senescence-associated marker p16^INK4a^ and SA-β-gal staining^33^. Two weeks post-surgery, Lepr^Cre^-tdTomato^+^p16^INK4a^⁺ and Lepr^Cre^-tdTomato^+^ SA-β-gal⁺ cells were increased in the exhausted group, with a more pronounced effect in females (Fig. 2d, e and Supplementary Fig. 5b, c). Additionally, qRT-PCR analysis of FACS-sorted CD45^-^Ter119^-^CD31^-^ bone marrow mesenchymal cells confirmed that *Cdkn2a* and *Cdkn1a* expression levels were significantly elevated in the exhausted group compared with the naive and control groups (Fig. 2f). However, further histological analysis revealed that p16^INK4a^ and SA-β-gal signals were predominantly detected in perilipin⁺ adipocytes rather than in LeprCre-tdTomato⁺ stromal cells (Fig. 2g). Consistently, *p16-creER*; *R26R*^tdTomato^ lineage cells labeled after the second injury showed that p16^+^ cells largely overlapped with adipocytes, whereas only a limited fraction overlapped with LepR⁺ stromal cells (Fig. 2h). These findings suggest that, although senescence-associated cells accumulate in the exhausted marrow environment, the exhausted phenotype of stromal cells is not simply explained by classical cellular senescence of the stromal population itself.

In summary, our findings demonstrate that exhausted bone marrow stromal cells undergo significant adipogenic differentiation, leading to elevated marrow fat accumulation. Although senescence-associated cells increased in the exhausted marrow environment, these signals were predominantly associated with adipocytes rather than stromal cells themselves. Thus, the exhausted phenotype is characterized primarily by adipogenic conversion and loss of stromal regenerative capacity, rather than being simply explained by classical cellular senescence of stromal cells.

### Stromal exhaustion occurs in fracture repair and persists after prolonged recovery

**Fig. 3:**
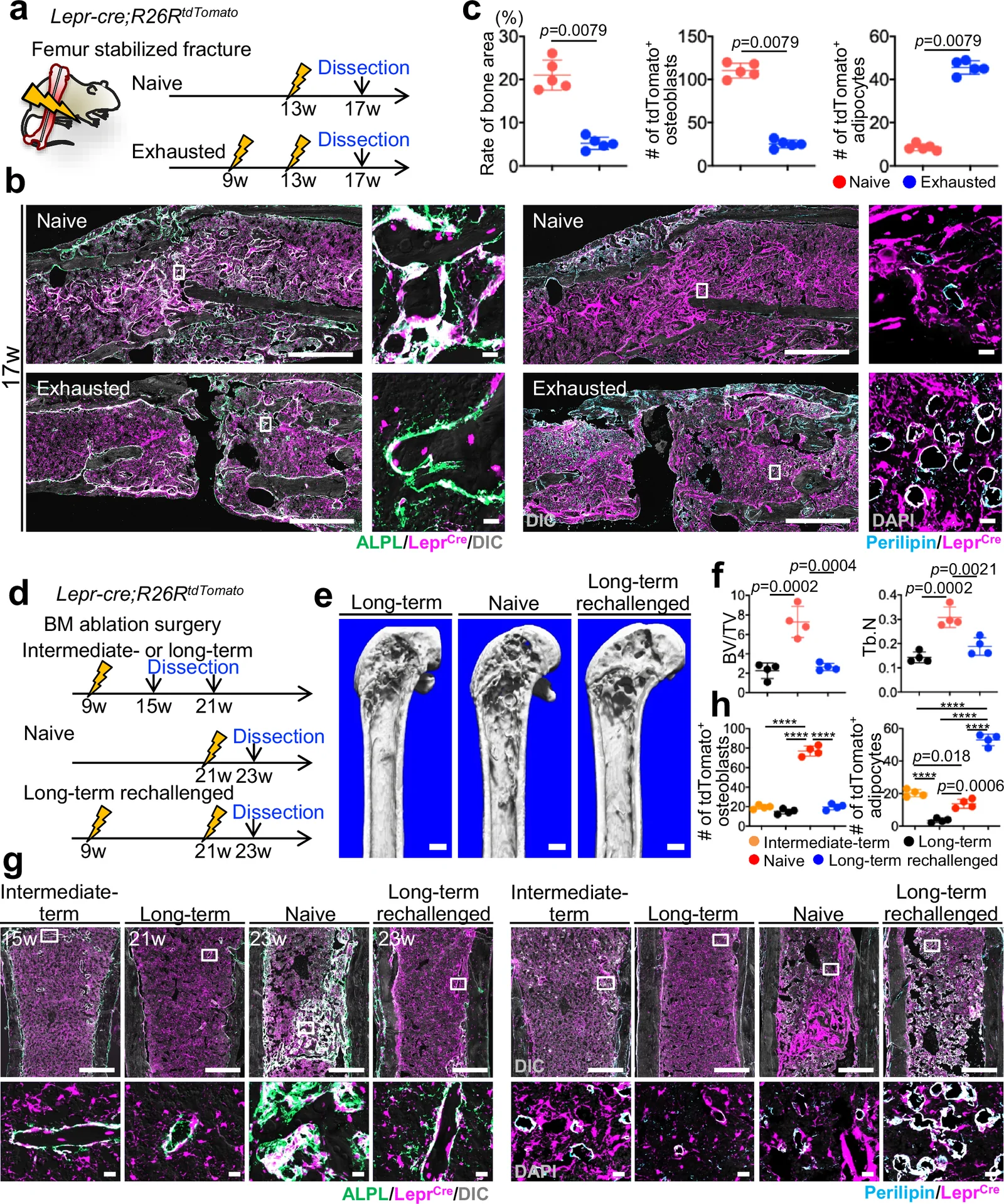
Stromal exhaustion occurs in fracture repair and persists after prolonged recovery. **a** Experimental design for stabilized femoral fracture in *Lepr-cre; R26R*^*tdTomato*^ mice. **b** Representative images of fracture callus regions from naive and exhausted mice at 17 weeks of age. Scale bars, 500 μm in low-magnification images and 20 μm in high-magnification images. **c** Quantification of the rate of bone area, tdTomato^+^ osteoblasts, and tdTomato^+^ adipocytes. *n* = 5 mice per group. **d** Experimental design for assessing persistent remodeling after long-term recovery and marrow rechallenge in *Lepr-cre; R26R*^*tdTomato*^ mice. **e** Representative micro-CT images of distal femurs from long-term, naive, and long-term rechallenged mice. Scale bars, 500 μm. **f** Quantification of trabecular BV/TV and Tb.N. *n* = 4 mice per group. **g** Representative images of distal femurs from intermediate-term, long-term, naive, and long-term rechallenged mice. Scale bar: upper panel, 500 μm; lower panel, 20 μm. **h** Quantification of tdTomato^+^ osteoblasts and tdTomato^+^ adipocytes. *n* = 4 mice per group. Exact P values are shown in the graphs; ****p < 0.0001. Two-tailed Mann-Whitney U test for (**c**). Two-tailed, One-way ANOVA followed by Tukey’s post hoc test for (**f, h**). Data are presented as mean ± S.D. Source data are provided as a Source Data file.

To determine whether stromal exhaustion also occurs during fracture repair, we next examined Lepr^Cre+^ lineage cells using a stabilized femoral fracture model (Fig. 3a). In this model, repeated fracture resulted in a reduced amount of newly formed bone and a decreased contribution of Lepr^Cre+^ lineage cells to osteoblasts at the repair site (Fig. 3b, c). In contrast, Lepr^Cre+^ lineage cells showed an increased contribution to marrow adipocytes after repeated fracture (Fig. 3b, c). These findings are consistent with the phenotype observed after repeated bone marrow ablation surgery and indicate that stromal exhaustion is not specific to the ablation model, but can also occur during fracture repair.

We further examined whether the four-week interval after the first injury was insufficient for recovery and whether stromal exhaustion reflected a transient delay rather than a sustained functional alteration. To address this, we performed a long-term rechallenge experiment, with a 12-week interval between the first and second bone marrow ablation surgeries (Fig. 3d). Micro-CT analysis revealed robust trabecular bone regeneration in the naive group, whereas the long-term rechallenged group showed markedly reduced trabecular bone formation after the second injury (Fig. 3e). Quantitative analysis confirmed significantly lower BV/TV and trabecular number in the long-term rechallenged group than in the naive group (Fig. 3f). We then examined the histological changes underlying this impaired regenerative response. After a single marrow ablation surgery at 9 weeks of age, marrow adipocytes were modestly increased by 15 weeks of age but had largely subsided by 21 weeks of age, by which time the marrow cavity appeared largely histologically restored, with re-established reticular stromal cells and limited residual adipocyte accumulation (Fig. 3g, h). Importantly, however, this apparent histological recovery did not indicate restoration of injury-induced osteogenic regenerative capacity. In the long-term rechallenged group, Lepr^Cre+^ lineage cells exhibited a markedly reduced osteogenic response and enhanced adipogenic differentiation after the second injury (Fig. 3g, h).

These findings indicate that, although injury-induced adipocyte accumulation can subside after a single injury, stromal cells do not regain their injury-induced osteogenic regenerative capacity. Thus, stromal exhaustion is not merely a transient delay in recovery or persistent adipocyte accumulation at baseline, but rather reflects a persistent functional alteration of injury-experienced marrow stromal cells.

### Inducible Cxcl12-creER lineage tracing validates stromal exhaustion in pre-existing reticular stromal cells

To further validate stromal exhaustion using a temporally controlled lineage-tracing approach, we utilized tamoxifen-inducible *Cxcl12-creER; R26R*^*tdTomato*^ mice to trace a subset of bone marrow reticular stromal cells, including adipogenic Cxcl12^+^ stromal cells (Supplementary Fig. 6a). In contrast to constitutive *Lepr-cre* labeling, *Cxcl12-creER* enables pulse labeling of Cxcl12⁺ stromal cells present at the time of tamoxifen administration, allowing their fate to be followed through subsequent injury and repair. By administering tamoxifen at 8 weeks of age before injury, we specifically traced pre-existing Cxcl12^+^ stromal cells during single and repeated injury. Consistent with findings in *Lepr-cre; R26R*^*tdTomato*^ mice, *Cxcl12-creER*–labeled (Cxcl12^CE^) cells showed robust osteogenic contribution in the naive group, whereas this response was markedly reduced in the exhausted group at both 2 and 4 weeks post-surgery (Supplementary Fig. 6b–d). Importantly, Cxcl12^CE^ cells persisted within the bone marrow after both single and repeated injury, indicating that pre-existing Cxcl12⁺ stromal cells were not eliminated by injury. Conversely, the contribution of Cxcl12^CE^ cells to marrow adipocytes was significantly increased after injury, particularly in the exhausted group (Supplementary Fig. 6e–g). These findings indicate that pre-existing Cxcl12⁺ reticular stromal cells survive repeated injury but undergo a functional shift from osteogenic toward adipogenic differentiation, further validating stromal exhaustion in a temporally defined CAR cell subset.

### Injury-induced regenerating osteoblasts/precursors transition back to the stromal state

**Fig. 4:**
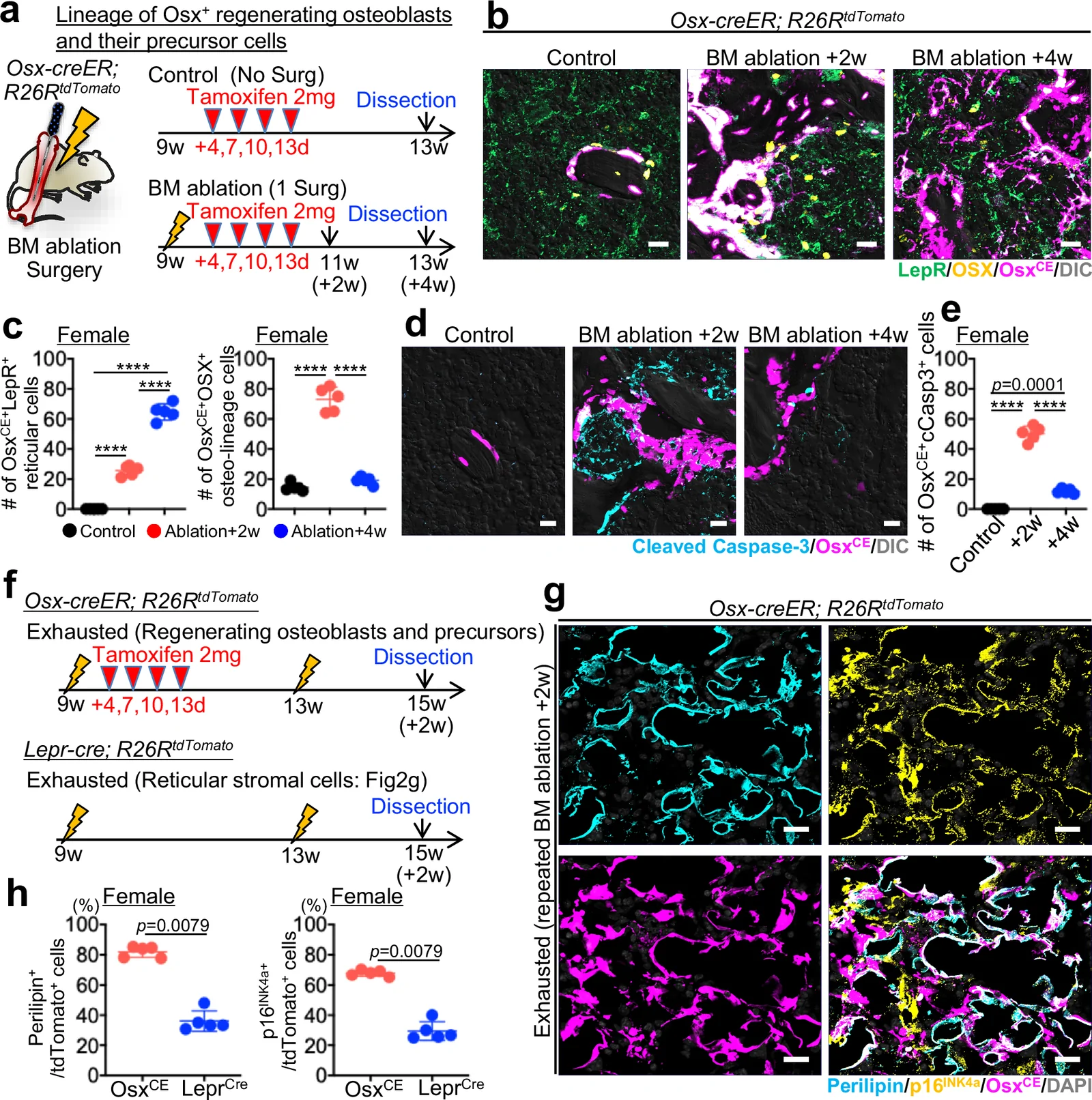
A subset of regenerating osteoblasts/precursors reverts to the reticular state and acquires features of exhaustion. **a** Experimental design for tracking the fate of regenerative osteoblasts using *Osx-creER; R26R*^*tdTomato*^ mice. Representative double immunofluorescence images for LepR and OSX proteins (**b**) and quantification of Osx^CE+^LepR^+^ stromal cells and Osx^CE+^OSX^+^ osteoblasts/osteocytes (**c**) in femurs at 2 and 4 weeks post-surgery compared to control. Scale bar: 20 μm. *n* = 5 mice per group. Immunofluorescence staining (**d**) and quantification (**e**) of Osx^CE+^Cleaved Caspase-3^+^ apoptotic cells in the femur. Scale bar: 20 μm. *n* = 5 mice per group. **f** Experimental design for tracking the fate of Osx^CE+^ and Lepr^Cre+^ exhausted stromal cells using *Osx-creER; R26R*^*tdTomato*^ and *Lepr-cre; R26R*^*tdTomato*^ mice. **g** Representative double immunofluorescence images staining for Perilipin and p16^INK4a+^ at 2 weeks post-surgery. Scale bar: 20 μm. **h** Quantification of the proportion of Perilipin^+^ adipocytes and p16^INK4a+^ senescent cells within the total Osx^CE^-tdTomato^+^ and Lepr^Cre^-tdTomato^+^ populations at 2 weeks post-surgery. *n* = 5 mice per group. Exact P values are shown in the graphs; ****p < 0.0001. Two-tailed, One-way ANOVA followed by Tukey’s post hoc test for (**c, e**). Two-tailed Mann-Whitney U test for (**h**). Data are presented as mean ± S.D. Source data are provided as a Source Data file.

We have elucidated that osteoblasts and their precursors are transformed from Lepr^+^Cxcl12^+^ reticular stromal cells in response to injury, and original stromal cells contribute to adipocyte formation following repeated injury. However, it remained unclear whether stromal cell-derived osteogenic cells revert to their original stromal state. To address this, we traced the fates of injury-driven regenerating osteoblasts and their immediate precursor cells using *Osterix (Osx)-creER; R26R*^*tdTomato*^ mice. Femur bone marrow ablation surgery was performed at 9 weeks of age, followed by tamoxifen injections at days 4, 7, 10, and 13 post-surgery to label Osx^+^ regenerating osteoblasts and their immediate precursor cells. Femurs were dissected at 2- and 4-weeks post-surgery (Fig. 4a). To define the initially labeled Osx^CE^⁺ population, we performed short-chase analyses during regeneration. When tamoxifen was injected at day 4 or day 13 after injury and femurs were dissected 2 days later, Osx^CE^⁺ cells predominantly overlapped with OSX⁺ osteogenic cells, whereas only a minimal fraction overlapped with LepR⁺ stromal cells. (Supplementary Fig. 7a–c). At 2-weeks post-surgery, OsxCE⁺ cells were still predominantly labeled as OSX⁺ osteogenic cells, but the fraction contributing to LepR⁺ stromal cells was slightly increased compared with the short-chase analysis (Fig. 4b, c). By 4-weeks post-surgery, however, a substantially greater proportion of Osx^CE^⁺ descendants contributed to the LepR⁺ stromal population, and the number of Osx^CE^⁺OSX⁺ cells decreased to the control level (Fig. 4b, c), suggesting that Osx^+^ regenerating osteogenic cells revert to the stromal state over time, rather than remaining permanently differentiated. To uncover the precise lineage of regenerating osteoblasts and their precursor cells, we examined whether apoptosis contributes to this process using Cleaved Caspase-3 immunohistostaining. At 2-weeks post-surgery, a significant number of Osx^CE^⁺ cells underwent apoptosis compared to the control (Fig. 4d, e), whereas this was not observed at 4-weeks. These findings suggest that regenerating osteogenic cells undergo multiple fates, including apoptosis, reversion to the stromal state, and persistence in the osteogenic lineage.

Next, we investigated whether Osx^CE+^ descendant stromal cells were in a more exhausted state. By comparing Osx^CE+^ regenerating osteoblast-derived stromal cells with Lepr^Cre+^ total stromal cells, we assessed the differences in adipogenic differentiation and cellular senescence 2-weeks following repeated injury (Fig. 4f). Interestingly, Osx^CE^⁺ descendant cells exhibited a greater propensity for adipogenic differentiation and higher levels of cellular senescence compared to Lepr^Cre+^ total stromal population (Figs. 4g, h and  2g). These findings suggest that reticular stromal cells reverted from regenerating osteoblasts are more susceptible to adipogenesis and senescence-associated features following injury.

Together, these results suggest that regenerating osteogenic cells exhibit multiple possible fates, including apoptosis, persistence within the osteogenic lineage, and potential return to a stromal-like state. Such stromal-like cells may become functionally altered and more susceptible to exhaustion, thereby limiting regenerative capacity after repeated injury.

### Stromal exhaustion is distinct from physiological aging

**Fig. 5:**
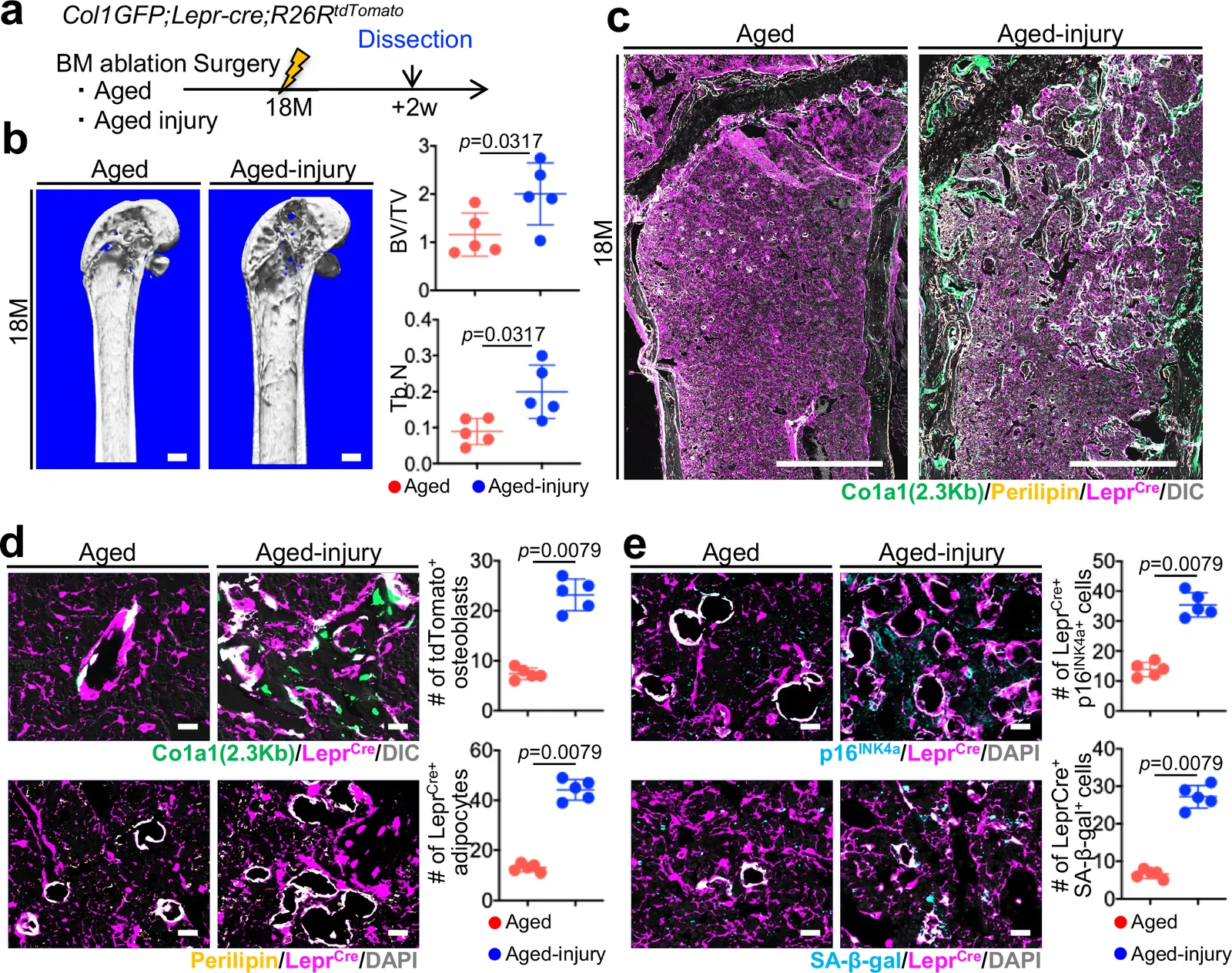
Lepr^+^ aged stromal cells retain injury-induced osteogenic potential. **a** Experimental design for bone marrow ablation in *Col1a1(2.3 kb)-GFP; Lepr-cre; R26R*^*tdTomato*^ aged (18-month-old) mice. Aged-injury mice were analyzed 2 weeks after bone marrow ablation surgery. **b** Representative micro-CT images of distal femurs and quantification of trabecular BV/TV and Tb.N. Scale bars, 500 μm. *n* = 5 mice per group. **c** Representative images of distal femurs. Scale bars, 500 μm. **d** Representative magnified images and quantification of tdTomato^+^ osteoblast-lineage cells and tdTomato^+^ adipocytes. Scale bars, 20 μm. *n* = 5 mice per group. **e** Representative magnified images and quantification of p16^INK4a+^tdTomato^+^ cells and SA-β-gal^+^tdTomato^+^ cells. Scale bars, 20 μm. *n* = 5 mice per group. Exact P values are shown in the graphs. Two-tailed Mann-Whitney U test. Data are presented as mean ± S.D. Source data are provided as a Source Data file.

To determine whether stromal exhaustion simply reflects physiological aging of bone marrow stromal cells, we examined the regenerative response of aged stromal cells after injury. We performed bone marrow ablation surgery in 18-month-old *Lepr-cre*; *R26R*^tdTomato^ mice and compared their response with that of no-surgery controls (Fig. 5a). Micro-CT analysis revealed that significant trabecular bone formation still occurred after injury (Fig. 5b). Histological analysis further showed that Lepr^Cre+^ lineage cells in aged mice retained the ability to contribute to osteoblasts after injury, whereas adipocyte accumulation was limited in aged mice under steady-state conditions and increased only modestly after injury (Fig. 5c, d). We also examined senescence-associated changes and found that the aged group showed only limited senescence-associated signals under steady-state conditions, although these signals increased after injury (Fig. 5e). Together, these results suggest that aged stromal cells retain injury-induced regenerative capacity, despite partial reductions in osteogenesis and increases in adipogenesis or senescence-associated features. Thus, stromal exhaustion is distinct from physiological aging and represents an injury-induced loss of regenerative capacity rather than a simple age-associated decline.

### Single-cell transcriptomic profiling reveals distinct stromal exhaustion and aging signatures

**Fig. 6:**
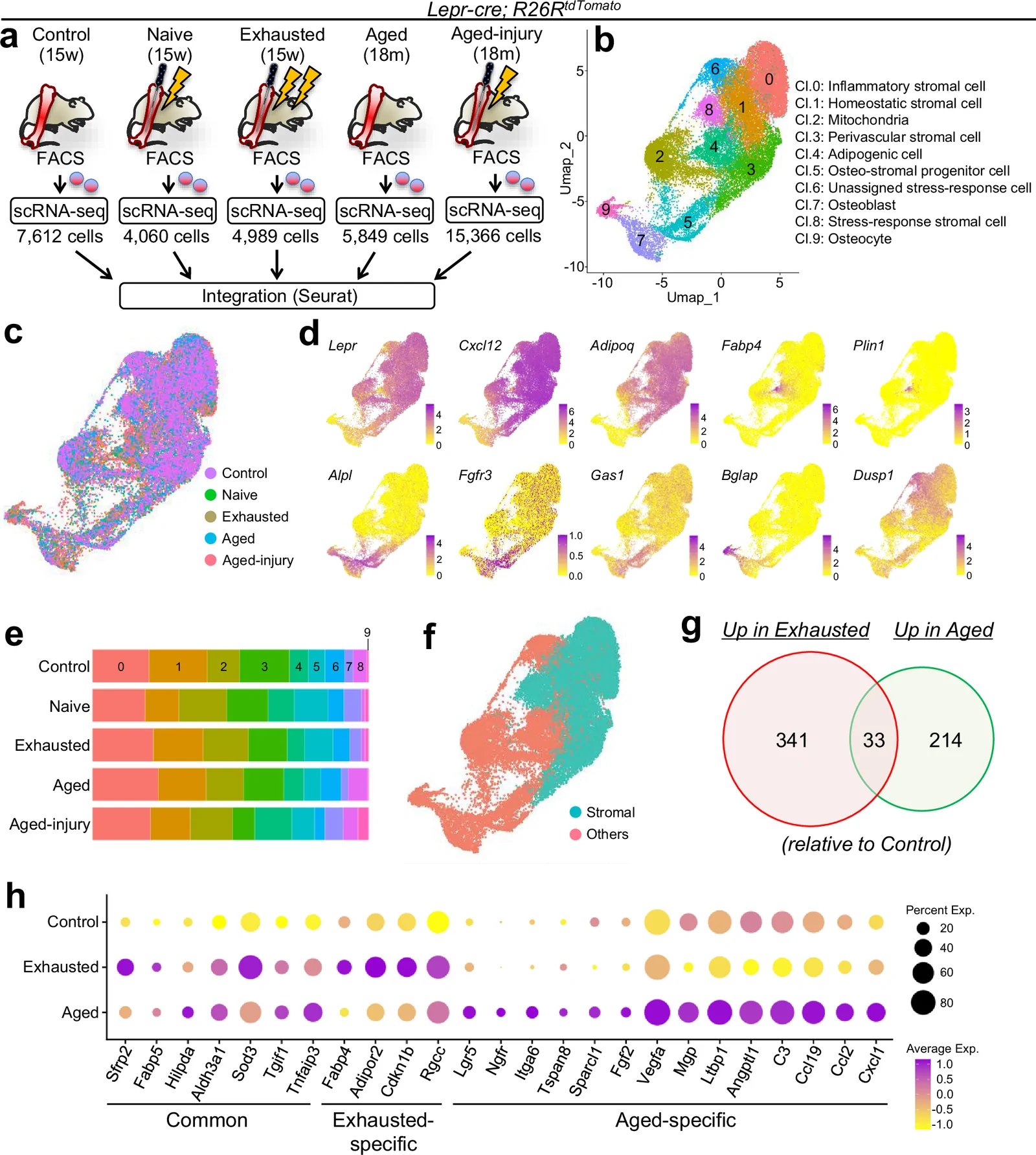
Single-cell transcriptomic profiling delineates distinct signatures of stromal exhaustion and aging. **a** Schematic of single-cell RNA-seq analysis using Seurat integration of Lepr^Cre^-tdTomato^+^ cells from five groups: control, naive, exhausted, aged (18-month-old), and aged-injury. **b** UMAP visualization of major sub-clusters (Clusters 0–9) of integrated datasets. **c** UMAP visualization of five datasets merged by Seurat. **d** Feature plots showing expression of canonical markers used for cluster annotation: *Lepr*, *Cxcl12*, *Adipoq* (stromal cell), *Fabp4*, *Plin1* (adipogenic cell), *Alpl, Fgfr3, Gas1* (osteo-stromal progenitor cell), *Bglap* (osteoblast/osteocyte), and *Dusp1* (stress-responsive cell). **e** Stacked bar plots showing the relative proportions of each cluster across five groups. **f** UMAP plot reclassified into two aggregated clusters (stromal and other mesenchymal) for focused analysis of whole stromal cells. **g** Venn diagram of genes upregulated in exhausted versus aged stromal cells (relative to control). **h** Representative dot plot showing selected differentially expressed genes in control, exhausted, and aged stroma: commonly upregulated in both exhausted and aged stroma, specific to exhausted stroma, and specific to aged stroma.

To define the transcriptomic features of exhausted stromal cells induced by repeated injury, we performed scRNA-seq analyses on fluorescence-activated cell sorting (FACS)-isolated Lepr^Cre^-tdTomato^+^ cells from five groups: uninjured controls at 15 weeks, naive at 15 weeks (surgery at 13 weeks of age), exhausted at 15 weeks (surgeries at 9 and 13 weeks of age), aged at 18 months (Fig. 6a), and aged-injury at 18 months (1 week post-surgery). These datasets were computationally integrated using Seurat, and a total of 37,876 cells were distributed to 12 transcriptionally distinct clusters (Supplementary Fig. 8a, b). We then extracted *tdTomato*^+^ skeletal cell clusters inside the bone, including clusters 0, 1, and 2 (Supplementary Fig. 8a), comprising a total of 28,626 cells, and subsequently re-clustered these cells into 10 clusters (Fig. 6b–e). Cluster annotation based on canonical markers identified four stromal subtypes (Cl. 0,1,3,8: *Lepr*, *Cxcl12*, *Adipoq*), two osteoblastic clusters (Cl. 7,9: *Bglap*, *Dmp1, Ifitm5*), one adipogenic cluster (Cl. 4: *Fabp4*, *Plin1*), osteo-stromal progenitor cluster (Cl.5: *Alpl, Fgfr3, Gas1*), stress-responsive cluster (Cl. 6,8: *Dusp1, Egr1*), and mitochondrial cluster (Cl. 2: *mt-Nd1, mt-Co2*) (Fig. 6d). The naïve, exhausted, and aged-injury groups, which underwent injury, exhibited an increased proportion of mitochondrial cluster (Cl. 2) (Control: 11.7%, Naive: 17.2%, Exhausted: 16.4%, Aged: 13.6%, Aged-injury: 15.3%) compared to the control group (Fig. 6e). The increase in the mitochondrial cluster may reflect elevated metabolic activity, cellular stress, or cellular exhaustion. Notably, the proportion of adipogenic cells did not substantially differ among groups in the scRNA-seq dataset. However, mature adipocytes are difficult to capture efficiently by single-cell RNA-seq because of their large size and fragility during tissue dissociation and processing. Therefore, adipocyte abundance, particularly in the exhausted group, may be underestimated in our scRNA-seq analysis despite clear histological evidence of adipocyte accumulation. These findings suggest that a subset of stromal cells may transition into a metabolically stressed or functionally altered state following injury, potentially contributing to stromal exhaustion.

To investigate the characteristics of exhausted stromal cells, we first analyzed gene expression differences between exhausted and aged stroma, relative to the control, to distinguish the effects of repeated injury from those of natural aging. We consolidated the 10 clusters into two broad categories—stromal and other mesenchymal—and re-isolated stromal cells for differential expression analysis (Fig. 6f). Interestingly, exhausted and aged stromal cells shared only 33 upregulated genes, with most differentially expressed genes being distinct between the two groups. This finding highlights the transcriptional divergence in cellular state between exhaustion and aging (Fig. 6g). Comparative transcriptomic analysis revealed that exhausted and aged stromal cells exhibit distinct molecular signatures. Although both populations share a subset of reactive stromal features, including genes associated with wound response, stress response, and extracellular matrix remodeling, exhausted stroma was uniquely characterized by enhanced adipogenic programming (*Fabp4*, *Adipor2*) and cell cycle regulation/arrest-associated genes (*Cdkn1b*, *Rgcc*), consistent with functional exhaustion. In contrast, aged stromal cells retained genes associated with stromal maintenance and niche-supportive functions, including *Lgr5*, *Itga6*, *Vegfa*, *Fgf2*, *Sparcl1*, and *Ltbp1* (Fig. 6h). While aging is generally associated with functional decline, these features suggest that aged stroma retains a degree of responsiveness and regenerative capacity compared to exhausted stroma, which is in a more advanced state of dysfunction associated with impaired lineage plasticity and enhanced adipogenic bias.

To further distinguish stromal exhaustion from physiological aging, we compared exhausted stroma with aged-injury stroma (Supplementary Fig. 9a, b). Pathway enrichment analysis revealed that aged-injury stroma retained injury-responsive regenerative programs, including Wnt signaling, TGF-β signaling, connective tissue development, and vasculature development. In contrast, exhausted stroma showed enrichment of inflammatory and stress-associated pathways, including TNF signaling, NF-κB/STAT3-related responses, and extracellular matrix remodeling. These findings indicate that aged stromal cells maintain coordinated injury-responsive regenerative programs, whereas exhausted stroma fails to properly reactivate these pathways after repeated injury, further supporting stromal exhaustion as a distinct injury-induced state rather than a simple extension of physiological aging.

### Repeated injury promotes stromal exhaustion and inhibits Wnt/β-catenin signaling

To uncover the molecular drivers of stromal exhaustion, we performed differential gene expression analysis across three groups: exhausted, naive, and uninjured control cells. First, we compared gene expression profiles between naive and uninjured control cells. Upregulated genes in naive stroma were associated with ECM organization, skeletal system development, and ossification, as identified by volcano plot and Gene Ontology analysis (Supplementary Fig. 9c, d). Key osteogenic transcription factors such as *Runx2*, *Sp7*, and *Smad1* were predicted to regulate the expression of genes upregulated in naive stroma (Supplementary Fig. 9e). These findings suggest that naive stroma is transcriptionally primed for regeneration with active ECM remodeling and enhanced osteogenic differentiation.

**Fig. 7:**
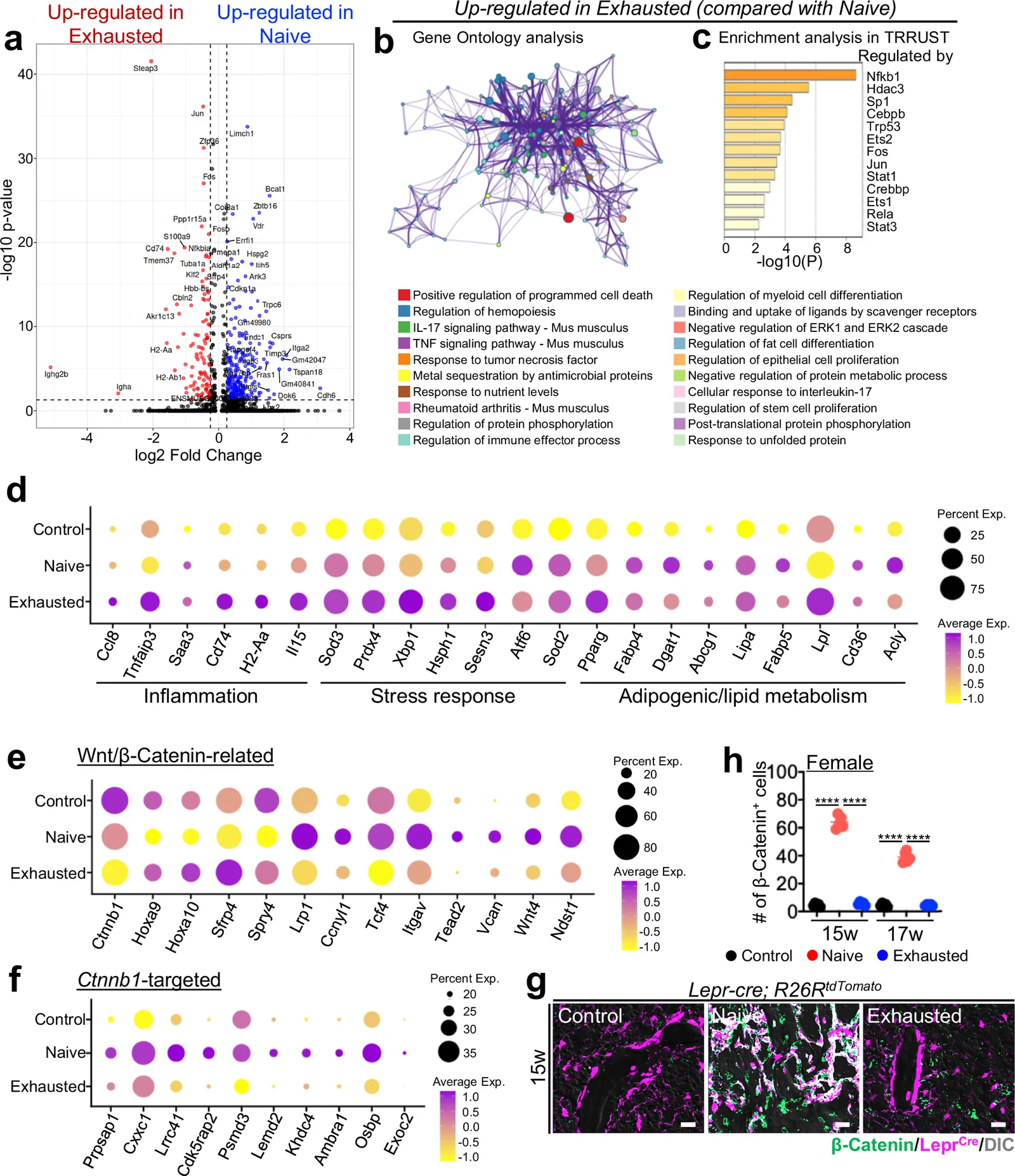
Repeated injury promotes stromal exhaustion and inhibits Wnt/β-catenin signaling. **a** Volcano plot of differential gene expression between exhausted and naive stromal cells. Upregulated genes in exhausted stroma (red) and naive stroma (blue). **b** Gene Ontology network of pathways enriched among genes upregulated in exhausted stroma. **c** Transcription-factor enrichment analysis for exhaustion-upregulated genes, based on the TRRUST database. **d** Dot plot visualization of representative genes enriched in control, naive, and exhausted stromal cells, grouped by functional categories: inflammation, stress response, and adipogenic/lipid metabolism. **e** Dot plot of canonical Wnt/β-catenin pathway-related genes in control, naive, and exhausted stromal cells. **f** Dot plot of representative potential target genes for *Ctnnb1* using Chip atlas in control, naive, and exhausted stromal cells. Representative immunofluorescence images of β-catenin (**g**) and quantification (**h**) in the femur among the control, naive, and exhausted groups at 15 weeks of age. Scale bar: 20 μm. *n* = 5 mice per group. ****p < 0.0001. Two-tailed, One-way ANOVA followed by Tukey’s post hoc test. Data are presented as mean ± S.D. Source data are provided as a Source Data file.

We next conducted differential gene expression analysis comparing the exhausted and naive groups. Exhausted stroma exhibited a distinct transcriptional profile, with many genes specifically upregulated compared with the naive group. In contrast, naive stroma showed enrichment for genes involved in tissue remodeling and regenerative activity (Fig. 7a). Gene Ontology analysis of genes upregulated in exhausted stroma revealed enrichment for inflammatory signaling, immune-related remodeling, cellular stress responses, and impaired cellular homeostasis. Terms such as IL-17 signaling, TNF signaling, response to tumor necrosis factor, regulation of myeloid cell differentiation, response to nutrient levels, response to unfolded protein, and regulation of fat cell differentiation suggest that exhausted stroma adopts a stress-responsive and inflammatory state with increased adipogenic bias (Fig. 7b). TRRUST-based transcription factor analysis^34^ supported the involvement of upstream regulators associated with stromal exhaustion, including those linked to inflammation (Nfkb1, Rela), cellular stress and senescence (Trp53, Hdac3), and adipogenic–metabolic regulation (Cebpb). These findings indicate that exhausted stromal cells adopt a transcriptional state marked by inflammatory activation, stress susceptibility, metabolic imbalance, and impaired lineage homeostasis (Fig. 7c). Supporting these findings, exhausted stromal cells overexpressed markers of inflammation (e.g., *Ccl8*, *Tnfaip3*, *Cd74*), cellular stress response (e.g., *Prdx4*, *Xbp1*, *Hsph1*), and adipogenic/lipid metabolism (e.g., *Pparg*, *Lipa*, *Lpl*) (Fig. 7d), indicating that exhausted stromal cells exhibit a state of chronic inflammation and heightened stress response, accompanied by disrupted metabolic homeostasis and thereby reflecting a truly exhausted cellular state.

We previously elucidated that the canonical Wnt/β-catenin signaling pathway activates the osteogenic lineage shift of stromal cells during bone regeneration^10^. We hypothesized that the loss of Wnt/β-catenin activity leads to stromal exhaustion. Exhausted stromal cells exhibit downregulation of *Ctnnb1* and other key components of the Wnt/β-catenin signaling pathway (e.g., *Lrp1*, *Ccnyl1*, *Tcf4*), along with upregulation of Wnt signaling antagonists (e.g., *Sfrp4*, *Spry4*). Notably, expression of the Wnt ligand *Wnt4* was downregulated in exhausted stroma (Fig. 7e). In addition, expression of several predicted *Ctnnb1* target genes identified by ChIP-Atlas^35^, including *Cxxc1*, *Cdk5rap2*, and *Psmd3*, were markedly reduced in exhausted stroma compared to naive stroma (Fig. 7f). Immunofluorescent staining validated that β-catenin protein was significantly increased in naive versus exhausted stroma at 2- and 4-weeks post-injury (Fig. 7g, h). These results suggest that suppression of Wnt/β-catenin activity may contribute to the establishment or maintenance of stromal exhaustion.

### Wnt/β-catenin signaling regulates injury-induced stromal exhaustion and its rescue

**Fig. 8:**
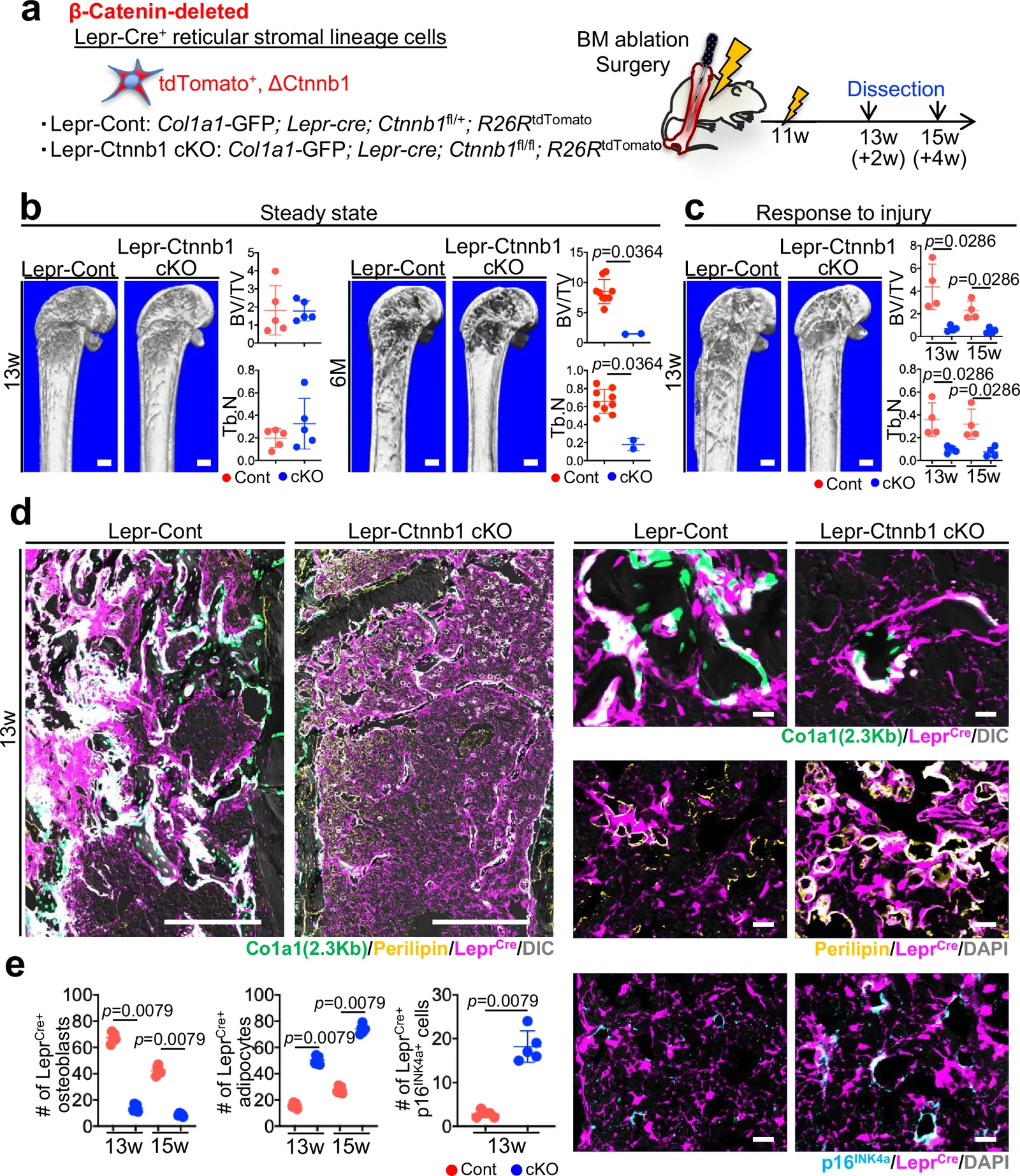
*Ctnnb1*-deficient bone marrow stromal cells enhance exhaustion in response to injury. **a** Experimental design for control (*Col1a1-GFP*; *Lepr-cre*; *Ctnnb1*^fl/+^; *R26R*^tdTomato^) and β-catenin conditional knockout (cKO: *Col1a1-GFP*; *Lepr-cre*; *Ctnnb1*^fl/fl^; *R26R*^tdTomato^) female mice. Bone marrow ablation surgery at 11 weeks, followed by analysis at 13 and 15 weeks of age. **b** Representative micro-CT images and quantitative analysis for BV/TV and Tb.N in distal femurs from Lepr-Control and Lepr-Ctnnb1 cKO mice under steady-state conditions at 13 weeks of age (left) and 6 months of age (right). Scale bars, 500 μm. *n* = 5 mice per group for 13 weeks; *n* = 9 (control) and *n* = 2 (cKO) for 6 months. **c** Representative micro-CT images of control and cKO distal femurs (left) at 13 weeks and quantitative analysis (right) at 13 and 15 weeks in response to injury. Scale bar: 500 μm. *n* = 4 mice per group. **d** Distal femur images of control and cKO with Col1a1-GFP (left, right upper), Perilipin (left, right center), and P16^INK4a^ (right lower) staining at 13 weeks. Scale bar: left panels, 500 μm; right panels, 20 μm. **e** Quantitative analysis of the number of Col1-GFP^+^ osteoblasts (left), Perilipin^+^ adipocytes (center), and p16^INK4a+^ cells (right) overlapped with Lepr^Cre^-tdTomato^+^ cells. *n* = 5 mice per group. Exact P values are shown in the graphs. Two-tailed, Mann–Whitney U test. Data are presented as mean ± S.D. Source data are provided as a Source Data file.

Lastly, to further elucidate the functional role of Wnt/β-catenin signaling in reticular stromal cells during bone regeneration, we conditionally deleted *Ctnnb1* in Lepr^+^ lineage cells. We analyzed *Col1a1*-GFP; *Lepr-cre*; *Ctnnb1*^fl/+^; *R26R*^tdTomato^ (Control) and *Col1a1*-GFP; *Lepr-cre*; *Ctnnb1*^fl/fl^; *R26R*^tdTomato^ (Lepr-Ctnnb1 cKO) mice under uninjured conditions at 13 weeks of age, and under injured conditions at 13 and 15 weeks of age following surgery performed at 11 weeks of age (Fig. 8a). Micro-CT analysis of femurs revealed no significant differences in trabecular BV/TV or Tb. N between control and Lepr-Ctnnb1 cKO mice under uninjured conditions at 13 weeks of age (Fig. 8b). In contrast, Lepr-Ctnnb1 cKO mice showed reduced trabecular bone mass at 6 months of age (Fig. 8b), suggesting that β-catenin signaling in Lepr⁺ stromal lineage cells becomes increasingly important for bone homeostasis as their contribution to the osteoblast lineage increases with age. However, following injury, Lepr-Ctnnb1 cKO mice exhibited reductions in both parameters (Fig. 8c), indicating that Wnt/β-catenin activity in stromal descendants is dispensable for maintaining skeletal homeostasis but essential for regeneration. Histological analysis revealed Lepr^Cre^-tdTomato^+^ cells in control mice gave rise to numerous Col1a1-GFP^+^ osteoblasts throughout regeneration, whereas Lepr^Cre^-ΔCtnnb1-tdTomato^+^ cells contributed to few osteoblasts (Fig. 8d, e). In contrast, Lepr^Cre^-ΔCtnnb1-tdTomato^+^ adipocytes were increased in Lepr-Ctnnb1 cKO mice even after one injury (Fig. 8d, e), suggesting that reticular stromal cells preferentially differentiate into adipocytes during regeneration and highlighting the role of β-catenin in maintaining the balance between osteogenesis and adipogenesis.

p16^INK4a^ staining revealed a greater number of senescent stromal cells in Lepr-Ctnnb1 cKO mice compared to controls, indicating that *Ctnnb1* deletion promotes cellular senescence (Fig. 8d, e). Thus, loss of *Ctnnb1* enhances adipogenic differentiation and cellular senescence in bone marrow stromal cells, potentially contributing to impaired regeneration following repeated injury.

**Fig. 9:**
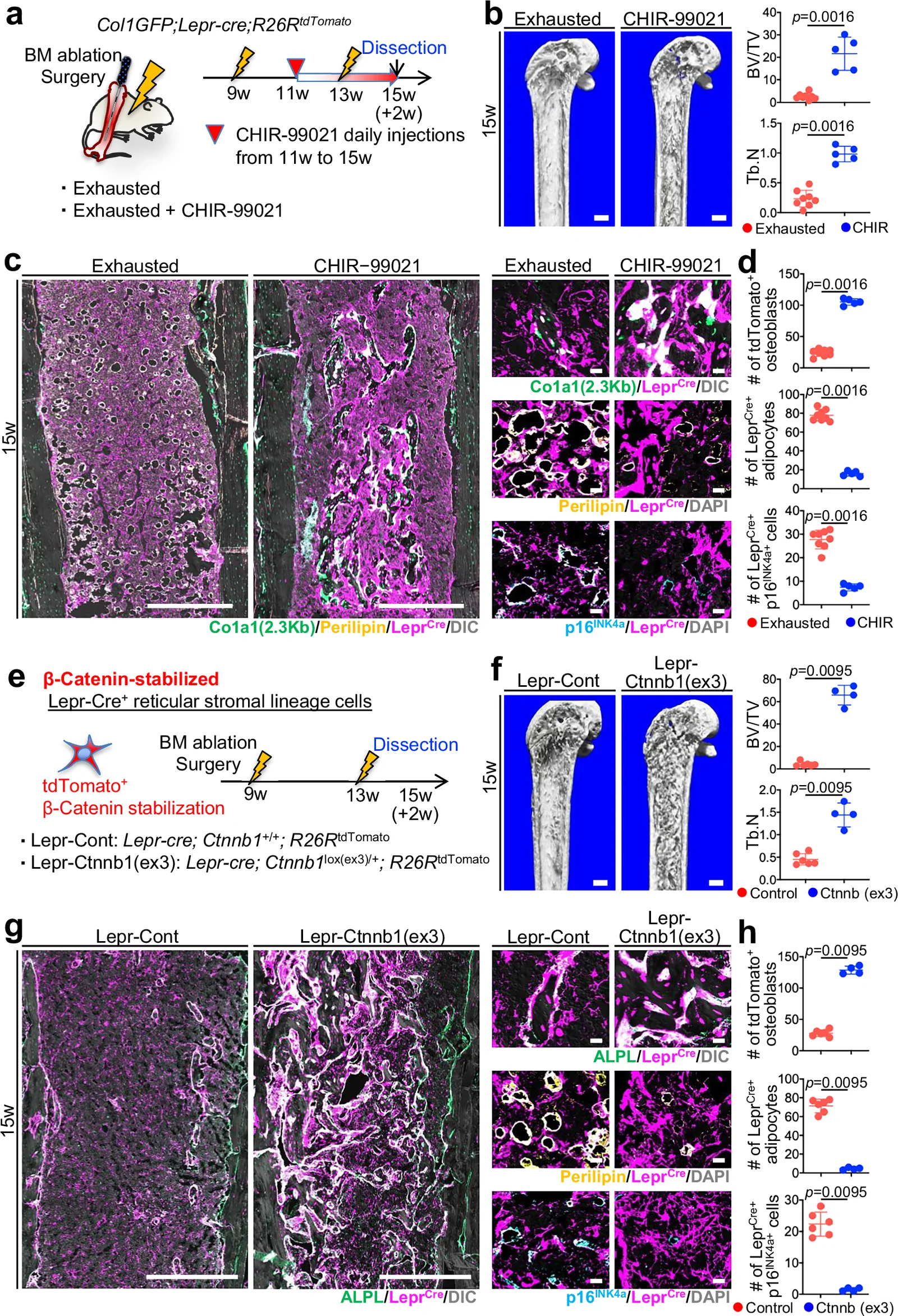
Pharmacological or genetic activation of β-catenin restores osteogenesis and suppresses adipogenic conversion. **a** Experimental design for pharmacological activation of Wnt/β-catenin signaling in exhausted *Col1a1(2.3 kb)-GFP; Lepr-cre; R26R*^*tdTomato*^ mice. CHIR-99021 was administered daily from 11 to 15 weeks of age, prior to analysis. **b** Representative micro-CT images and quantification of trabecular bone parameters in distal femurs at 15 weeks of age. Scale bars, 500 μm. *n* = 8 (exhausted) and *n* = 5 (CHIR-99021-treated) mice. **c** Representative images of exhausted and CHIR-99021-treated mice with Col1a1-GFP (left, right upper), Perilipin (left, right center), and P16^INK4a^ (right lower) staining at 15 weeks. Scale bars, 500 μm (left) and 20 μm (right). **d** Quantification of tdTomato^+^ osteoblasts, adipocytes, and p16^INK4a+^ cells at 15 weeks of age. *n* = 8 (exhausted) and *n* = 5 (CHIR-99021-treated) mice. **e** Experimental design for genetic stabilization of β-catenin in Lepr^Cre+^ cells. Control (*Lepr-cre*; *Ctnnb1*^+/+^; *R26R*^tdTomato^) and β-catenin stabilized (Ctnnb1(ex3): *Lepr-cre*; *Ctnnb1*^lox(ex3)/+^; *R26R*^tdTomato^) female mice. Surgeries were performed at 9 and 13 weeks of age and analyzed at 15 weeks. **f** Representative micro-CT images and quantification of trabecular bone parameters. Scale bars, 500 μm. *n* = 6 (control) and *n* = 4 (Ctnnb1(ex3)) mice. **g** Representative images of distal femurs of control and Ctnnb1(ex3) with ALPL (left, right upper), Perilipin (right center), and P16^INK4a^ (right lower) staining at 15 weeks. Scale bars, 500 μm (left) and 20 μm (right). **h** Quantification of tdTomato^+^ osteoblasts, adipocytes and p16^INK4a+^ cells. *n* = 6 (control) and *n* = 4 (Ctnnb1(ex3)) mice. Exact P values are shown in the graphs. Two-tailed, Mann–Whitney U test. Data are presented as mean ± S.D. Source data are provided as a Source Data file.

To determine whether activation of Wnt/β-catenin signaling could reverse the exhausted phenotype, we next performed pharmacological and genetic rescue experiments. First, we administered daily injections of CHIR-99021, a GSK-3 inhibitor that activates Wnt/β-catenin signaling, from 11 to 15 weeks of age, during the period of the second bone marrow ablation surgery in the repeated-injury model (Fig. 9a). Micro-CT analysis revealed that CHIR-99021 treatment significantly increased trabecular bone regeneration after repeated injury, as shown by increased BV/TV and Tb.N compared with untreated exhausted mice (Fig. 9b). Histological analysis showed that CHIR-99021 restored the contribution of Lepr^Cre^ ⁺ stromal lineage cells to Col1a1-GFP⁺ osteoblasts while reducing the accumulation of Lepr^Cre^⁺ adipocytes and p16^INK4a^-positive cells in the bone marrow (Fig. 9c, d). These findings indicate that pharmacological activation of Wnt/β-catenin signaling partially rescues the impaired osteogenic response and suppresses adipogenic conversion in exhausted stroma.

We further examined whether genetic activation of β-catenin in Lepr⁺ stromal lineage cells could rescue stromal exhaustion. For this purpose, we used *Lepr-cre*; *Ctnnb1*^lox(ex3)/+^; *R26R*^tdTomato^ (Lepr-Ctnnb1(ex3)) mice, in which β-catenin is constitutively stabilized in Lepr^Cre^⁺ cells (Fig. 9e). Following repeated injury, Lepr-Ctnnb1(ex3) mice showed increased bone mass compared with exhausted controls (Fig. 9f). Histological analysis further revealed that β-catenin stabilization preserved osteogenic differentiation of Lepr^Cre^⁺ stromal lineage cells and markedly suppressed their adipogenic conversion, with a concomitant reduction in p16^INK4a^ expression in the exhausted marrow environment (Fig. 9g, h).

In summary, our findings demonstrate that β-catenin plays a critical role during bone regeneration by maintaining osteogenic differentiation and suppressing adipogenesis and cellular senescence in bone marrow reticular stromal cells. Conditional deletion of *Ctnnb1* in Lepr^+^ cells during regeneration impairs osteoblast formation, promotes adipogenesis, and accelerates senescence, ultimately disrupting bone regeneration and marrow homeostasis. Conversely, pharmacological activation or genetic stabilization of β-catenin restored osteogenesis and suppressed adipogenic conversion after repeated injury. Together, these loss- and gain-of-function approaches identify impaired Wnt/β-catenin reactivation as a key mechanism underlying stromal exhaustion.

## Discussion

**Fig. 10:**
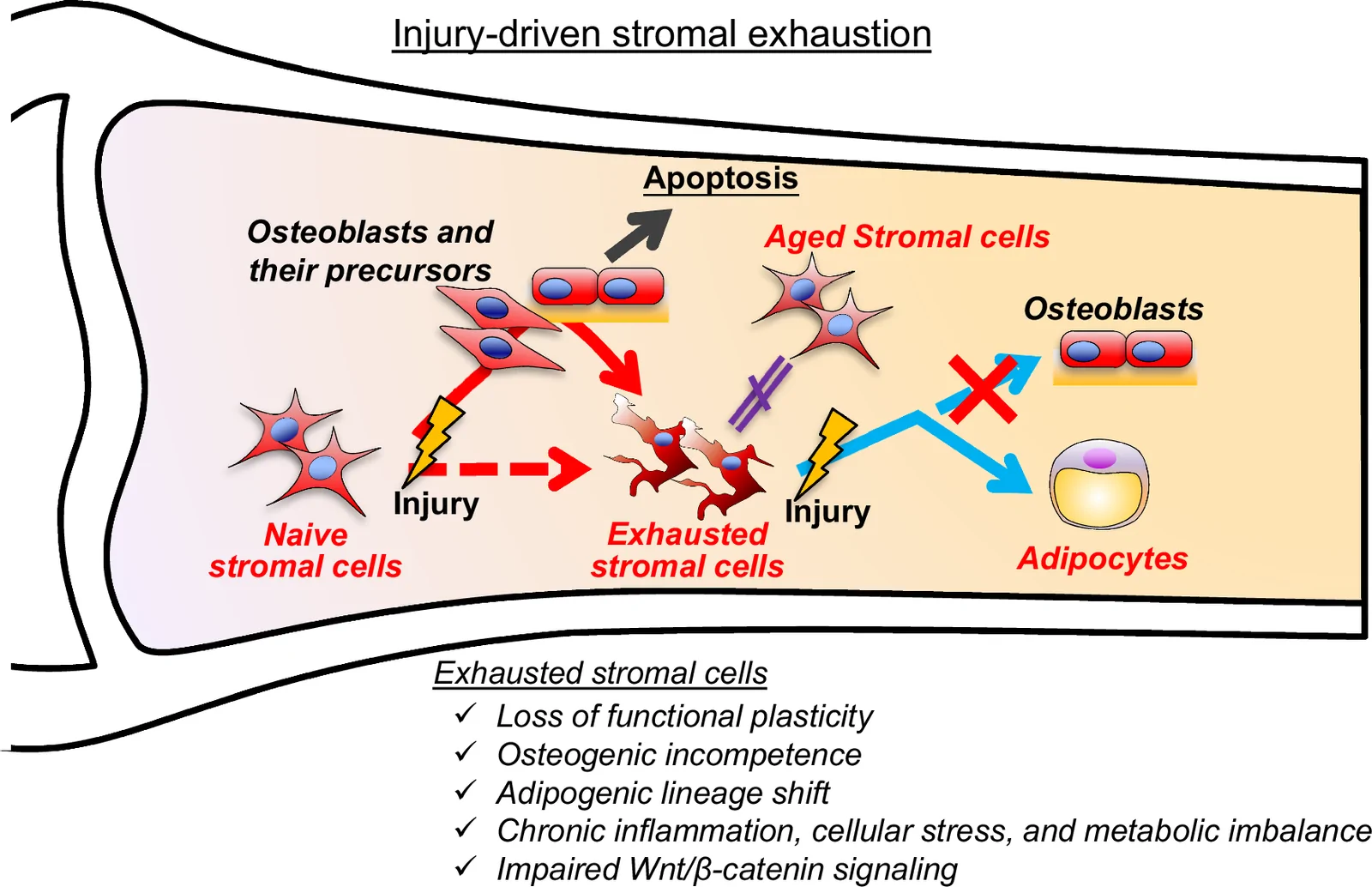
Dynamics and key features of exhausted reticular stromal cells driven by injury. Lepr^+^Cxcl12^+^ naive reticular stromal cells have a highly bone regenerative capability in response to injury, differentiating into osteoblasts and reverting to the stromal state. However, injury drives stromal exhaustion instead of complete restoration. This dysfunctional state shows the loss of functional plasticity, osteogenic incompetence, and adipogenic lineage shift, induced by suppression of Wnt/β-catenin signaling, as supported by β-catenin deletion-induced adipogenesis and partial rescue by pharmacological or genetic activation of Wnt/β-catenin signaling, particularly under the conditions of chronic inflammation, cellular stress, and metabolic imbalance. Importantly, aged stromal cells show a profile distinct from exhausted stromal cells, retaining injury-induced regenerative capability rather than undergoing an adipogenic lineage shift.

Here, we characterized a previously underappreciated state of stromal exhaustion induced by mechanical injury (Fig. 10). Lepr^+^Cxcl12^+^ bone marrow reticular stromal cells, which ordinarily support hematopoiesis and contribute to osteoblast formation during regeneration, exhibit substantial plasticity under stress. Although these stromal cells initially differentiate into osteoblasts and their precursor cells upon injury, the reverted stroma-like cells are functionally distinct from the original ones. They exhibit features of exhaustion, including impaired clonogenicity, diminished osteogenic capacity, and increased adipogenic characteristics, particularly under conditions of chronic inflammation, cellular stress, and metabolic imbalance. Although bone marrow adipocytes can exhibit reversibility under certain conditions, such as after irradiation^36^, our findings suggest that repeated mechanical injury may induce a distinct, less reversible form of stromal dysfunction.

Injury-driven stromal exhaustion is orchestrated primarily through suppression of the Wnt/β-catenin signaling pathway, a critical regulator of stromal identity and osteogenic potential^10^. Loss of Wnt/β-catenin activity not only promotes adipogenic shift. Our transcriptomic and lineage-tracing analyses reveal that regenerating osteoblasts and their precursors can revert into the stromal cell pool in a qualitatively altered state. These reverted stromal cells, although morphologically similar to their original counterparts, harbor transcriptional signatures of exhaustion, which contribute to the accumulation of dysfunctional stromal cells within the marrow niche. This observation expands the classical view of progenitor exhaustion from a simple depletion of cell numbers to a more nuanced model involving qualitative deterioration and subsequent functional disturbance of the niche. The enrichment of IL-17 signaling and Toll-like receptor pathways in exhausted stroma further suggests that the bone marrow immune microenvironment may contribute to stromal exhaustion. Repeated injury may generate a sustained inflammatory microenvironment that interferes with regenerative stromal responses, including Wnt/β-catenin–associated osteogenic reactivation, and instead promotes maladaptive remodeling.

Both repeated injury and natural aging lead to stromal dysfunction and marrow adiposity; however, our analyses reveal that these processes give rise to fundamentally distinct cellular states. Injury-induced exhaustion is characterized by a pronounced loss of regenerative and supportive functions, with stromal cells exhibiting features of adipogenic commitment. In contrast, aging-associated stromal cells, despite reduced osteogenic capacity, retain key properties such as stem/progenitor potential, vascular support and tissue maintenance, suggesting a more preserved and structurally intact niche environment. Consistent with previous reports describing functional decline of aged skeletal stem cells^37^, aging-associated stromal populations in our study also showed reduced osteogenic capacity. However, our data suggest that injury-driven exhaustion of bone marrow reticular stromal cells represents a distinct state from physiological skeletal cell aging, characterized by a more profound failure of injury-induced regenerative reactivation and enhanced adipogenic conversion after repeated injury. Thus, while both conditions converge on impaired osteogenesis and niche decline, the underlying mechanisms and degrees of reversibility differ substantially. These distinctions have important therapeutic implications, as exhaustion may respond to interventions targeting acute stress pathways, whereas age-related dysfunction may require strategies that preserve or enhance endogenous maintenance programs.

This exhaustion model may reflect an evolutionarily conserved limitation in tissue regeneration. Repeated amputation studies in axolotls^6^, a species known for its remarkable regenerative capacity, show a decline in regenerative potential over successive injuries. This suggests that functional exhaustion of progenitor populations is a fundamental biological constraint, even in organisms with robust regenerative abilities. Our findings align with this paradigm, proposing that injury-driven stromal exhaustion represents a limiting factor in skeletal regeneration.

Clinically, fracture history is a well-established predictor of subsequent fractures and the development of osteoporosis, particularly among older individuals^38,39^. This elevated risk is often attributed to pre-existing skeletal fragility and undiagnosed osteoporosis, especially in the context of low-energy or fragility fractures^5^. However, accumulating evidence indicates that increased fracture risk and progressive bone loss can also occur following trauma-related fractures in otherwise healthy individuals^5,40,41^, suggesting the contribution of additional, injury-induced mechanisms. Here, we propose a distinct pathogenic pathway in which bone fractures act as triggers for stromal exhaustion within the bone marrow niche. This process compromises the regenerative function of stromal cells and shifts the microenvironment toward adipogenic and osteoporotic phenotypes. Multiple studies have shown that adipogenic bone marrow has a reduced capacity for regeneration and contributes to increased fracture risk^42,43^. Such a functional decline may constitute an acquired and self-sustaining form of skeletal aging, independent of baseline bone mineral density. In addition to acquired conditions, injury-driven stromal exhaustion may also play a role in congenital disorders such as osteogenesis imperfecta (OI), in which patients experience lifelong recurrent fractures. Although OI is primarily caused by mutations in genes encoding type I collagen, repeated cycles of fracture and repair may impose chronic stress on the bone marrow niche, ultimately leading to progressive stromal dysfunction. This process could further impair bone regeneration and contribute to the osteoporosis-like phenotype frequently observed in OI^44^. This paradigm also applies to treatment-induced marrow injury, such as that caused by chemotherapy or radiotherapy. In such cases, damage to mesenchymal stromal cells induces premature senescence and adipogenic infiltration within the marrow cavity, ultimately delaying hematopoietic recovery and impairing immune reconstitution^45,46^. Similarly, in bone marrow failure syndromes such as myelodysplastic syndromes and aplastic anemia, mesenchymal stromal cells exhibit impaired proliferative capacity and reduced osteogenic support, accompanied by a shift toward adipogenic differentiation and increased inflammatory activation, all of which contribute to hematopoietic dysfunction^47–49^. Recent findings have demonstrated that stromal cells derived from patients with myelodysplastic syndromes and acute myeloid leukemia show a marked loss of osteogenic potential and bias toward adipogenesis^50^, suggesting that stromal exhaustion may constitute a central mechanism underlying the formation of pathological bone marrow niche formation.

Therapeutically, our rescue experiments identify Wnt/β-catenin signaling as a validated target for reversing key features of stromal exhaustion. Suppression of this pathway drives adipogenic bias and loss of osteogenic function in Lepr^+^ stromal cells, whereas pharmacological activation with CHIR-99021 or genetic stabilization of β-catenin restores osteogenic regeneration and suppresses adipogenic conversion after repeated injury. These findings suggest that restoring canonical Wnt signaling may re-establish regenerative stromal function within the exhausted marrow niche. Potential translational approaches include sclerostin-neutralizing antibodies (e.g., romosozumab), already approved for osteoporosis^51^, pharmacological activation via GSK3β inhibitors, or development of Wnt ligand mimetics. Furthermore, precision delivery systems such as nanoparticles or ligand-conjugated agents targeting Lepr^+^ or Cxcl12^+^ stromal subsets^52^ offer the potential for cell-specific modulation with minimal systemic toxicity. However, efficient delivery modalities for these therapies remain a therapeutic obstacle.

In conclusion, we identify injury-driven stromal exhaustion as a critical barrier to effective skeletal regeneration. This dysfunctional state, characterized by impaired osteogenesis and adipogenic lineage shift, reflects a loss of functional plasticity in bone marrow stromal cells. It is driven by suppression of Wnt/β-catenin signaling and exacerbated under conditions of chronic inflammation, cellular stress, and metabolic imbalance. Importantly, the reversibility of key exhausted features through Wnt/β-catenin activation suggests that stromal exhaustion is a therapeutically targetable form of regenerative failure. Stromal exhaustion provides a unifying framework for understanding impaired bone repair in diverse pathological contexts and opens new avenues for targeted regenerative therapies. Future investigations should focus on delineating the molecular checkpoints of exhaustion, defining the extent and durability of its reversibility, and developing interventions that restore the full regenerative potential of the bone marrow stromal niche.

## Methods

### Mouse strains

*Cxcl12-creER*^10^ and *Osx-creER*^53^ mice have been described previously. *Rosa26-CAG-loxP-stop-loxP-tdTomato* (Ai14: *R26R-tdTomato*, JAX007914), *Col1a1(2.3 kb)-GFP* (JAX013134), *Lepr-cre* (JAX008320), *Ctnnb-floxed* (JAX004152), and *C57BL/6 J* (JAX000664) mice were acquired from the Jackson laboratory. *BALB/c* (BALB/cCrSlc) mice were from Japan SLC. All experiments were performed in accordance with the protocols approved by the Animal Care and Use Committee of Nagasaki University (#2204261788 and #2404301942). *p16-creER* mice^54^ were provided by Dr. Makoto Nakanishi. *Ctnnb1*^lox(ex3)/+^ mice^55^ were provided by Dr. Makoto M. Taketo. All mice were housed in specific pathogen-free conditions and analyzed in a mixed or C57BL/6 J background. Mice were housed in static microisolator cages with ad libitum access to water and food. Animal rooms were climate-controlled to provide temperatures of 22–23 °C and 40–65% of humidity on a 12 h light/dark cycle. For all breeding experiments, *cre* and *creER* transgenes were maintained in male breeders to avoid spontaneous germline recombination. Mice were identified by micro-tattooing or ear tags. Tail biopsies of mice were lysed by a HotShot protocol (incubating the tail sample at 95 °C for one hour in an alkaline lysis reagent followed by neutralization) and used for PCR-based genotyping (DreamTaq Green PCR Master Mix, Thermo Fisher K1082). Mice were also genotyped fluorescently (Handy light, OptoCode LEDGFP-3WOF and LED530-3WRF) whenever possible. Mice were euthanized by overdose of carbon dioxide or decapitation under inhalation anesthesia in a drop jar (Isoflurane, Fujifilm 099-06571).

### Tamoxifen and induction of *cre-loxP* recombination

Tamoxifen (CAY 13258) was completely dissolved in 100% ethanol. Subsequently, a proper volume of sunflower seed oil (Wako DLJ6115) was added to the tamoxifen-ethanol mixture and rigorously mixed. The tamoxifen-ethanol-oil mixture was incubated at 50 °C in a chemical hood until the ethanol evaporated completely. The tamoxifen-oil mixture was stored at room temperature until use. Tamoxifen was injected at a dose of 2 mg into mice intraperitoneally using a 26-1/2-gauge needle (Terumo NN-2613S).

### Bone marrow ablation surgery

Mice were anesthetized with intraperitoneal injection of a mixture of medetomidine (0.3 mg/kg body weight), midazolam (4 mg/kg body weight), and butorphanol (5 mg/kg body weight). The right femurs were operated on, while the left femurs were untreated and used as an internal control. After skin incision, the cruciate ligaments of the knee were carefully separated using a dental excavator, and a hole was made in the intercondylar region of the femur using a 26-1/2-gauge needle (NN-2613S). The endodontic instruments (K-file, #35, 40, 45, and 50) (GC, Tokyo, Japan) were used in a stepwise manner to remove a cylindrical area of the marrow space. The surgical field was irrigated with saline, and the incision line was sutured.

### Stabilized femoral fracture

Under anesthesia, surgery was performed on the right femur, while the contralateral femur was used as an internal control. After a skin incision, the femoral shaft was carefully exposed by blunt dissection of the surrounding muscles. A stainless-steel intramedullary pin was inserted into the femoral medullary cavity to stabilize the femur. After pin insertion, a standardized transverse fracture was generated at the femoral shaft. The fracture position and fixation stability were confirmed intraoperatively. The surgical field was irrigated with saline, and the incision was closed with sutures.

### Pharmacological activation of Wnt/β-catenin signaling

CHIR-99021 (Selleckchem) was dissolved in DMSO and diluted in vehicle immediately before use, with a final DMSO concentration of 5–10% for in vivo administration. Mice received intraperitoneal injections of CHIR-99021 at a dose of 0.3 mg per mouse in a total volume of 200 µL per mouse, according to the experimental schedule indicated in the figures. Vehicle-treated mice received the same injection volume with the same final DMSO concentration.

### Three-dimensional micro-computed tomography analysis

Mice were anaesthetised by intraperitoneal injection. Whole animals were imaged in vivo on a Rigaku R_mCT micro-CT system (Rigaku Corporation, Tokyo, Japan) using the following parameters: 90 kV voltage, 100 µA current, 2 min exposure time and 20 µm/pixel resolution. Dissected femurs were then scanned ex vivo on a SkyScan 1272 system (Bruker, Billerica, MA, USA) at 60 kV, 166 µA, 19 min exposure time and 20 µm/pixel resolution. Raw projections were reconstructed into transverse slices and imported into TRI‑3D BON software (Ratoc Systems, Inc., Osaka, Japan; version R9.02.00.0-H-64) for three-dimensional morphometric analysis. Trabecular bone within a 3.6 mm segment immediately distal to the growth plate (towards the diaphysis) was analysed, and bone volume fraction (BV/TV) and trabecular number (Tb.N) were quantified.

### Histology and immunohistochemistry

Samples were fixed in 4% paraformaldehyde overnight at 4 °C, then decalcified in 15% EDTA for a proper period, typically ranging from 7 to 14 days. Subsequently, samples were cryoprotected in 30% sucrose/PBS solutions and then in 30% sucrose/PBS: OCT (1:1) solutions, each at least overnight at 4 °C. Samples were embedded in an OCT compound (Tissue-Tek, Sakura) and transferred on a sheet of dry ice to solidify the compound. Samples were cryosectioned at 7 µm using a cryostat (Leica CM1850) and adhered to positively charged glass slides (Matsunami MAS). Sections were postfixed in 4% paraformaldehyde for 10 min at room temperature. For immunostaining, sections were permeabilized with 0.25% TritonX/TBS for 10 min, blocked with 3% BSA/TBS for 30 min and incubated with rabbit anti-Perilipin A/B polyclonal antibody (1:200, Sigma, P1873), goat anti-ALPL polyclonal antibody (1:200, R&D systems, AF2910), goat anti-LepR polyclonal antibody (1:200, R&D systems, AF497), rat anti-CDKN2A/p16^INK4a^ monoclonal antibody (1:200, Abcam, ab241543), rabbit anti-Cathepsin K monoclonal antibody (1:200, Abcam, ab300569), rabbit anti-Cleaved Caspase-3 polyclonal antibody (1:200, Cell signaling, 9661), rabbit anti-Osterix polyclonal antibody (1:200, Abcam, ab22552), and rabbit anti-beta Catenin polyclonal antibody (1:200, Abcam, ab16051) overnight at 4^o^C, and subsequently with Alexa Fluor 633-conjugated goat anti-rabbit IgG (A21071), Alexa Fluor 633-conjugated goat anti-rat IgG (A21094), Alexa Fluor 488-conjugated donkey anti-goat IgG (A11055) (1:400, Invitrogen) or Alexa Fluor 488-conjugated donkey anti-rabbit IgG (A11034) (1:400, Invitrogen) for 2 hours at room temperature. Sections were further incubated with DAPI (4’,6-diamidino-2-phenylindole, 5 µg/ml, CAY 14285) to stain nuclei prior to imaging. For SA-β-gal staining, samples were incubated with SPiDER-βGal working solution diluted 1:2000 in citrate–phosphate buffer prepared from citric acid and Na₂HPO₄ at 37 °C for 1 h, washed with TBS, and imaged using 488-nm excitation.

### Imaging

Images were captured by an automated inverted fluorescence microscope with a structured illumination system (Zeiss Axio Observer 7 with ApoTome 3 system) and Zen 3 Pro (Blue Edition) software. A multicolor LED light system was used (Zeiss Colibri 7 RGB-UV) (385/475/555/630 nm). The filter settings used were: Filter Set 90 HE LED (Ex. 385/30, 469/38, 555/30, 631/33, Em. 425/30 + 514/30 + 592/25 + 709/100 nm) and Set 38 HE (Ex. 470/40, Em. 525/50 nm). The objectives used were: Fluar 2.5x/0.12, EC Plan-Neofluar 5x/0.16 Ph1, Plan-Apochromat 10x/0.45, Plan-Apochromat 20x/0.8, Plan-Apochromat 40x/0.95 Korr, Plan-Apochromat 63x/1.40. Images were typically tile-scanned with a motorized stage, Z-stacked, and reconstructed by a maximum intensity projection (MIP) function. Differential interference contrast (DIC) was used for objectives higher than 10x. Representative images of at least four independent biological samples are shown in the figures. Cell quantification on histological sections was manually performed by an independent investigator. To ensure accuracy, all counts were conducted in duplicate as technical replicates. For general cell quantification (excluding the specific adipocyte populations described below), cells were counted under 40× objective magnification using a systematic random sampling approach. Ten non-overlapping fields per sample were analyzed, covering at least 80% of the region of interest. Only cells with intact nuclei and clearly defined cellular boundaries were included. Specialized quantification protocols were applied for adipocytes in *Lepr-cre; R26R*^*tdTomato*^ and *Cxcl12-creER; R26R*^*t*dTomato^ mice (Fig. 2c and Supplementary Figs. 5a; 6g). These adipocytes were counted at 10x magnification through entire bone marrow cavities located below the growth plate. The counting zone was defined as the area between the chondro-osseous junction and the distal metaphyseal margin.

### Cell dissociation

Soft tissues and epiphyses were carefully removed from dissected femurs. After removing distal epiphyseal growth plates and cutting off proximal ends, femurs were cut roughly and incubated with 2 Wunsch units of Liberase^TM^ (Roche 5401127001) and 1 mg of Pronase (Roche 10165921001) in 2 ml Ca^2+^, Mg^2+^-free Hank’s Balanced Salt Solution (HBSS, Wako ACH7023) at 37 °C for 60 min. On a shaking incubator (Thermomixer C, Eppendorf). After cell dissociation, cells were mechanically triturated using an 18-gauge needle with a 2.5 ml Luer-Lok syringe (TERUMO SS-02LZ) and a pestle with a mortar (Coors Tek), and subsequently filtered through a 70 µm cell strainer (AS ONE 3-6649-02) into a 50 ml tube on ice to prepare a single-cell suspension. These steps were repeated three times, and dissociated cells were collected in the same tube. Cells were pelleted by centrifugation at 300 × *g* for 10 min and resuspended in an appropriate medium for subsequent purposes. For cell culture experiments, cells were resuspended in 10 ml culture medium and counted on a hemocytometer.

### Colony-forming assay

Nucleated bone marrow cells were plated into tissue culture 6-well plates (VIOLAMO 080723A001) at a density of <10^5^ cells/cm^2^, and cultured in low-glucose DMEM with GlutaMAX supplement (WAKO 04129775) and 10% FBS (Serana 48040123) containing penicillin-streptomycin (Sigma P0781) for 21–28 days. Cell cultures were maintained at 37 °C in a 5% CO_2_ incubator. Representative images of at least four independent biological samples are shown in the figures. For CFU-Fs, cells were fixed with 70% Ethanol for 5 min and stained with 0.5% Crystal Violet in 70% Ethanol.

### Cell sorting

Female mice from the same litter were used for cell sorting. Dissociated cells were stained by standard protocols with the following antibodies (1:500, Invitrogen/eBioscience). Allophycocyanin (APC)-conjugated CD31 (390, endothelial/platelet, 17-0311-82), CD45 (30F-11, hematopoietic, 17-0451-82), Ter119 (TER-119, erythrocytes, 17-5921-82). Cells were sorted on a three-laser BD FACS Aria II (BD Biosciences) (Ex.375/488/633 nm) high-speed cell sorter with a 100 µm nozzle, using the following gating hierarchy: Forward/side scatter (FSC-A/SSC-A) to exclude debris; Single cells: FSC-W vs FSC-H and SSC-W vs SSC-H; Lineage-negative: CD31^-^CD45^-^Ter119^-^; tdTomato^+^ (Supplementary Fig. 10). Acquired raw data were further analyzed on FlowJo software (TreeStar). Approximately 30,000 tdTomato^+^ cells were directly sorted into ice-cold DPBS/10% FBS, pelleted by centrifugation at 300 × g for 10 min, and resuspended in DPBS/1% FBS to a final concentration of ~100 cells/μl.

### Quantitative reverse-transcription PCR (qRT-PCR)

FACS-isolated CD31^-^CD45^-^Ter119^-^ cells were collected into ice-cold DPBS with 1 % BSA and pelleted by centrifugation at 300 × *g* for 10 min. Total RNA was extracted using the Direct-zol^TM^ RNA MicroPrep Kit (Zymo R2061-A) with on-column DNase I digestion. To further eliminate genomic DNA, a second treatment was performed using the DNA-free^TM^ DNA Removal Kit (Toyobo, FSQ-301). Extracted RNA was quantified and assessed for purity (A260/A280 and A260/A230) using a NanoDrop spectrophotometer, ensuring high-quality input (ratios ~2.0). First-strand cDNA was synthesized from 500 ng RNA using the ReverTra Ace® qPCR RT Master Mix (Toyobo FSQ-201) following the manufacturer’s protocol. qPCR was performed on a LightCycler® 480 (Roche) using THUNDERBIRD^TM^ SYBR® qPCR Mix (Toyobo QPS-201) with the following primers: *Cdkn2a* (Fw: 5′-GAGCGGGGACATCAAGACAT-3′, Rv: 5′-TAGCTCTGCTCTTGGGATTGG-3′), *Cdkn1a* (Fw: 5′-CTTGTCGCTGTCTTGCACTC-3′, Rv: 5′-GAGGGCTAAGGCCGAAGATG-3′) and *Actb* (Fw: 5′-GGCTGTATTCCCCTCCATCG-3′, Rv: 5′-CCAGTTGGTAACAATGCCATGT-3′). Actb was selected as the internal reference after confirming its expression stability across naive and exhausted groups using the geNorm algorithm. All reactions were run in technical triplicate, including no-template and no-RT negative controls. Cycling conditions: 95 °C for 5 min; 45 cycles of 95 °C for 10 s, 60 °C for 30 s; followed immediately by a melt-curve analysis to verify specificity. Relative gene expression was calculated using the ΔΔCt method, normalizing target gene Ct to Actb and expressing fold‑changes relative to the control group.

### Single cell RNA-seq analysis

Sorted cells were loaded onto the BD Rhapsody HT Xpress (BD Biosciences) following the manufacturer’s recommended protocols. cDNA or gDNA libraries were sequenced by Illumina NovaSeq X Plus. The sequencing data were first pre-processed using the BD software Seven Bridges. For alignment purposes, we generated and used a custom genome fasta and index file by including the sequences of *EGFP* and *tdTomato-WPRE* in the mouse genome (GRCm39).

### Computational analysis for scRNA-seq

Single-cell RNAseq datasets were acquired in-house and deposited in the National Center for Biotechnology Information (NCBI) Gene Expression Omnibus (GEO) under accession number GSE302366 (Figs. 6, 7 and Supplementary Figs. 8, 9) or downloaded from GEO under accession numbers GSE136970 and GSE136979 (Supplementary Fig. 1). Downstream analysis was performed using the Seurat R package (v.5.2.1)^56^. We filtered out cells with less than 1000 detected genes per cell and those with > 20 % mitochondrial reads (GSE302366) or > 15 % mitochondrial reads (GSE136970 and GSE136979). We also filtered out cells with > 4000 genes (GSE302366) or > 6000 genes (GSE136970 and GSE136979). The downstream processing comprised: (i) log-normalization with NormalizeData (normalization.method = “LogNormalize”); (ii) selection of highly variable genes with FindVariableFeatures (selection.method = “vst”); (iii) dataset integration using FindIntegrationAnchors followed by IntegrateData; (iv) scaling with ScaleData; (v) dimensionality reduction with RunPCA (PCA) and RunUMAP (UMAP); and (vi) unsupervised clustering with FindNeighbors and FindClusters. For Dataset 1, clusters 0, 1, and 2 (28,626 cells) were isolated and re-clustered, yielding 10 sub-clusters. These were manually curated into two cell-type categories—Stromal and Others. We also performed differential expression analysis between experimental groups for each cell-type using FindMarkers and conducted over-representation analysis of cell-type and group-specific up-regulated genes using Metascape^57^. For example, up-regulated genes in exhausted stromal compared with naive stromal (Fig. 7a) are defined as those with avg_log2FC < −0.5 and -log10(p_val_adj) > 2.

### Statistical analysis

Results are presented as mean values ± S.D. Statistical evaluation was conducted using the two-tailed Mann-Whitney *U*-test or two-tailed One-way ANOVA followed by Tukey’s post hoc test, using GraphPad Prism software. A *P* value of <0.05 was considered significant. No statistical method was used to predetermine sample size. Sample size was determined on the basis of previous literature and our previous experience to give sufficient standard deviations of the mean so as not to miss a biologically important difference between groups. The experiments were not randomized. All of the available mice of the desired genotypes were used for experiments. The investigators were not blinded during experiments and outcome assessment. One femur from each mouse was arbitrarily chosen for histological analysis. Genotypes were not particularly highlighted during quantification.

### Reporting summary

Further information on research design is available in the Nature Portfolio Reporting Summary linked to this article.

## Supplementary information

Supplementary Information

Reporting Summary

Transparent Peer Review file

## Source data

Source Data

## Data Availability

Source data are provided with this paper. The single-cell RNA-seq data presented herein have been deposited in the National Center for Biotechnology Information (NCBI)’s Gene Expression Omnibus (GEO), and are accessible through GEO Series accession number, SuperSeries GSE302366, including GSM9102740-GSM9102743 and GSM9744609. We processed scRNA-seq data generated in this study using the BD Rhapsody WTA Analysis Pipeline v2.3 on Seven Bridges Genomics, employing the GRCm39 (Release M32) mouse reference genome for alignment and transcript annotation. Previously published scRNA-seq datasets reanalyzed in this study are available under GEO accession numbers GSE136970 and GSE136979. Any additional data generated and/or analyzed during the current study are available from the corresponding author upon reasonable request. Source data are provided with this paper.
