# Supplementary material for: Injury-driven stromal exhaustion disrupts intrinsic regenerative capability
Source: Nat Commun. 2026 Aug 19;17:8259. doi: 10.1038/s41467-026-76108-z (PMC13490406; doi:10.1038/s41467-026-76108-z)
Supplement: Supplementary file 1 [file 41467_2026_76108_MOESM1_ESM.pdf]

Supplementary Information

Injury-driven stromal exhaustion disrupts intrinsic regenerative capability

Wu et al.

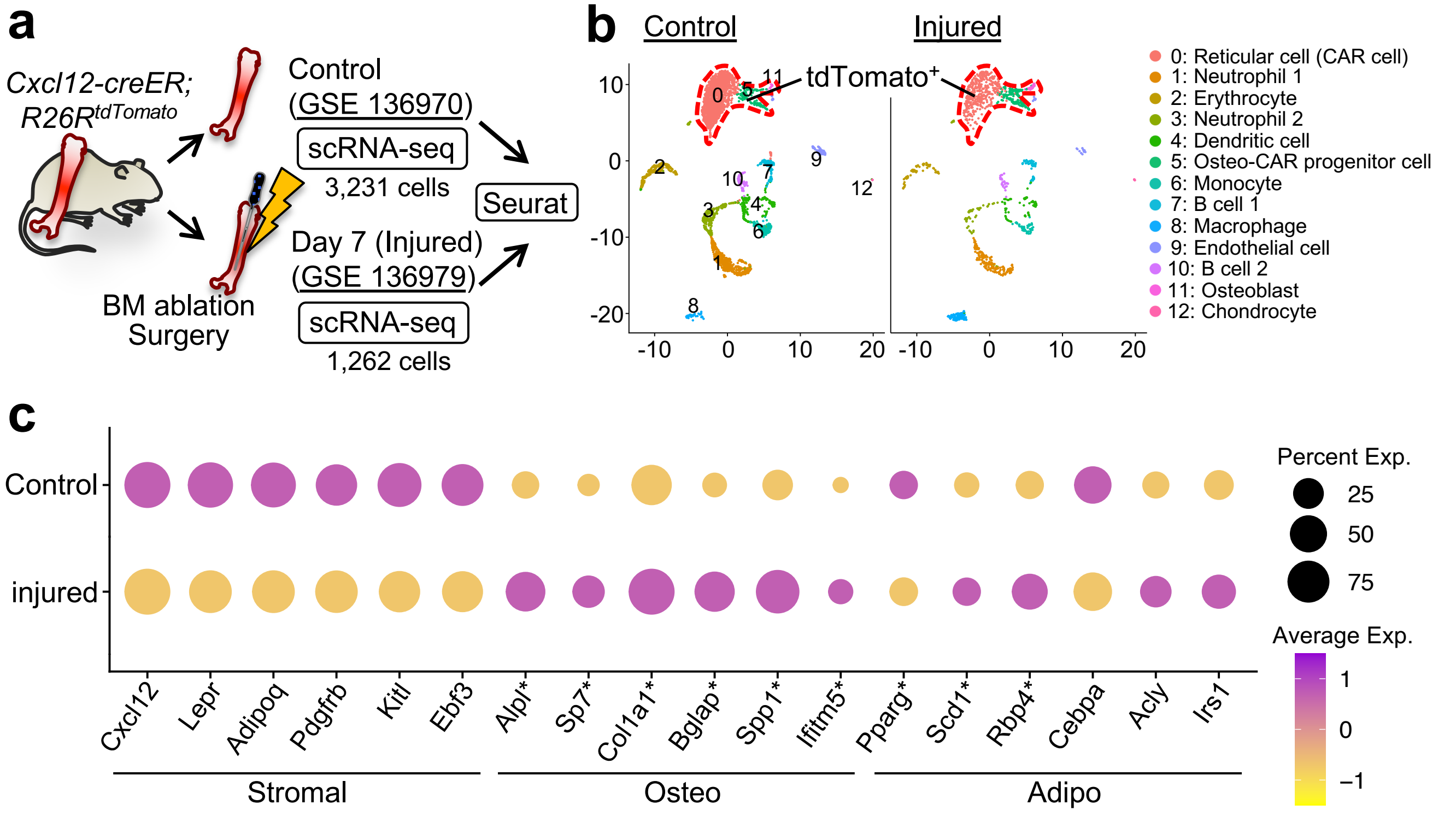

Supplementary Figure 1. Bone marrow stromal cells induce both osteogenic and adipogenic profiles upon injury

(a) Schematic overview of the scRNA-seq workflow. tdTomato$^+$ cells were FACS-isolated from femurs of *Cxcl12-creER; R26R^{tdTomato}* mice with or without bone marrow ablation surgery (datasets GSE136970 and GSE136979), and analyzed using the Seurat integration pipeline.
(b) UMAP visualization of transcriptionally distinct clusters (0–12), with tdTomato$^+$ populations highlighted (red dashed outline).
(c) Dot plot visualization of the expression of representative stromal, osteogenic, and adipogenic marker genes in control and injured conditions.

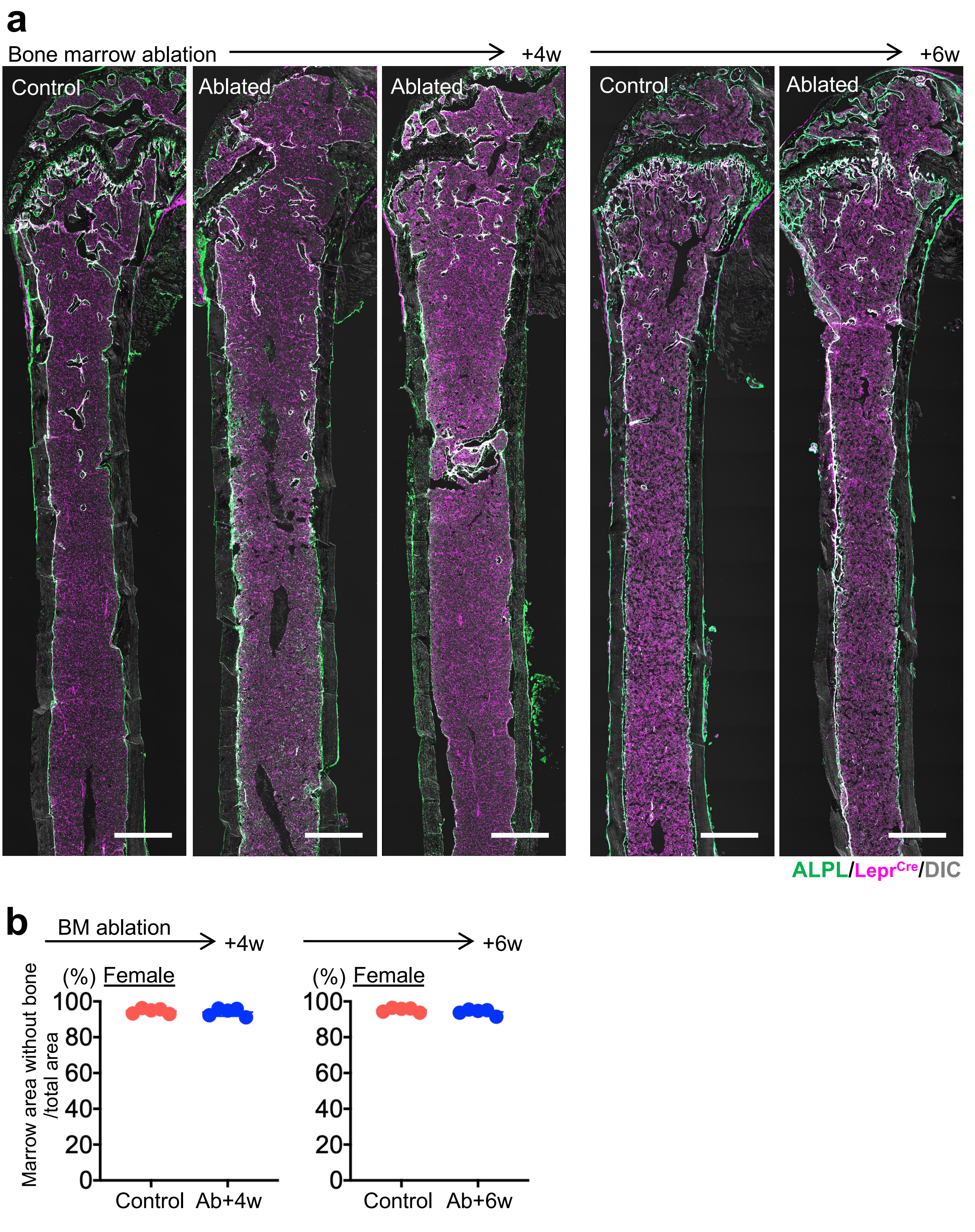

Supplementary Figure 2. Healing process of bone marrow ablation surgery

(a) *Lepr-cre; R26R*^{tdTomato} femurs with ALPL staining in control and injured groups at 4 (left) and 6 (right) weeks post-surgery. Scale bar: 500 µm.
(b) Quantitative analysis of the no-bone area over total bone marrow area at 4 (left) and 6 (right) weeks post-surgery. $n = 5$ mice per group.
Two-tailed, Mann-Whitney U test. Data are presented as mean ± S.D. Source data are provided as a Source Data file.

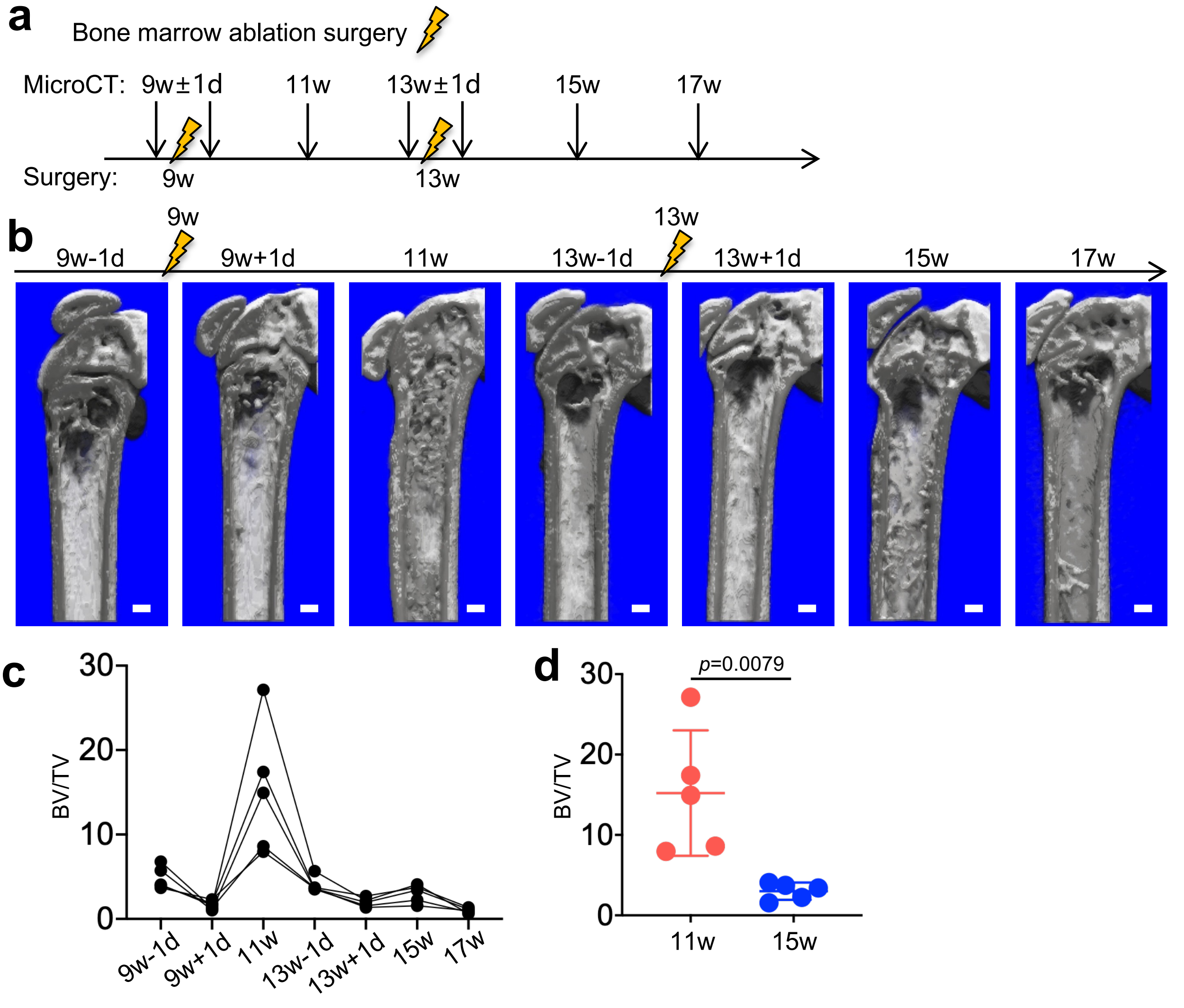

Supplementary Figure 3. Longitudinal *in vivo* micro-CT analysis across repeated bone marrow ablation

(a) Experimental design for longitudinal micro-CT analysis across repeated bone marrow ablation. Surgeries were performed at 9 and 13 weeks of age, and distal femurs were analyzed by *in vivo* micro-CT at the indicated time points.
(b) Representative micro-CT images of the same distal femoral region during healing. Scale bars, 500 μm.
(c) Quantification of BV/TV across the indicated time points. *n* = 5 mice.
(d) Quantification of BV/TV at 11 and 15 weeks of age. *n* = 5 mice per group.
Exact P values are shown in the graphs. Two-tailed, Mann–Whitney U test. Data are presented as mean ± S.D. Source data are provided as a Source Data file.

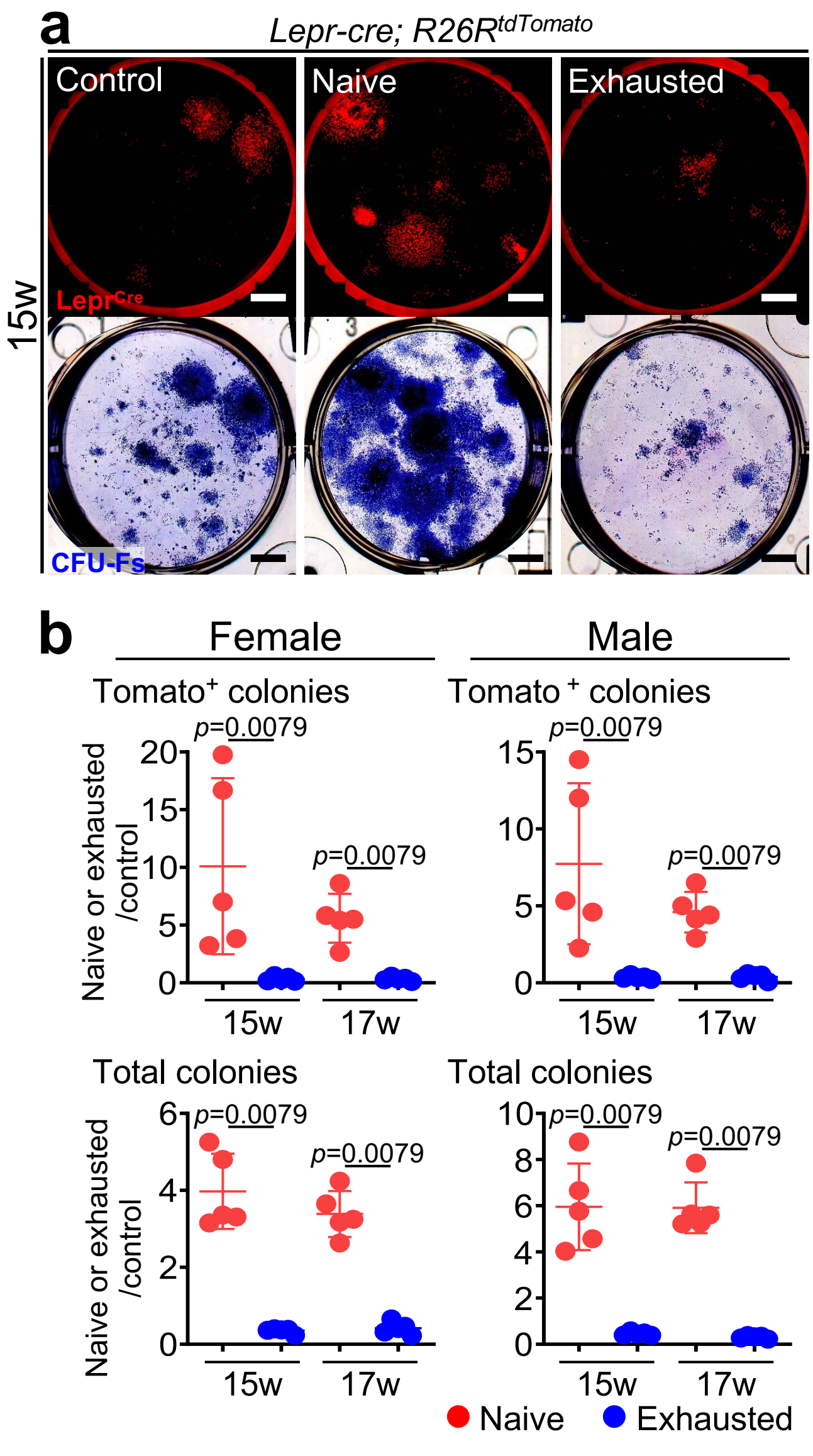

Supplementary Figure 4. *ex vivo* CFU-F assay reveals impaired proliferative capability of exhausted stromal cells

(a) Representative images of $Lepr^{Cre}$-$tdTomato^{+}$ colonies (upper) and total CFU-Fs in control, naive, and exhausted groups. Scale bar: 5 mm.

(b) Quantification of $tdTomato^{+}$ colonies and total CFU-Fs in naive and exhausted groups over control. $n = 5$ mice per each group.

Exact P values are shown in the graphs. Two-tailed, Mann–Whitney U test. Data are presented as mean ± S.D. Source data are provided as a Source Data file.

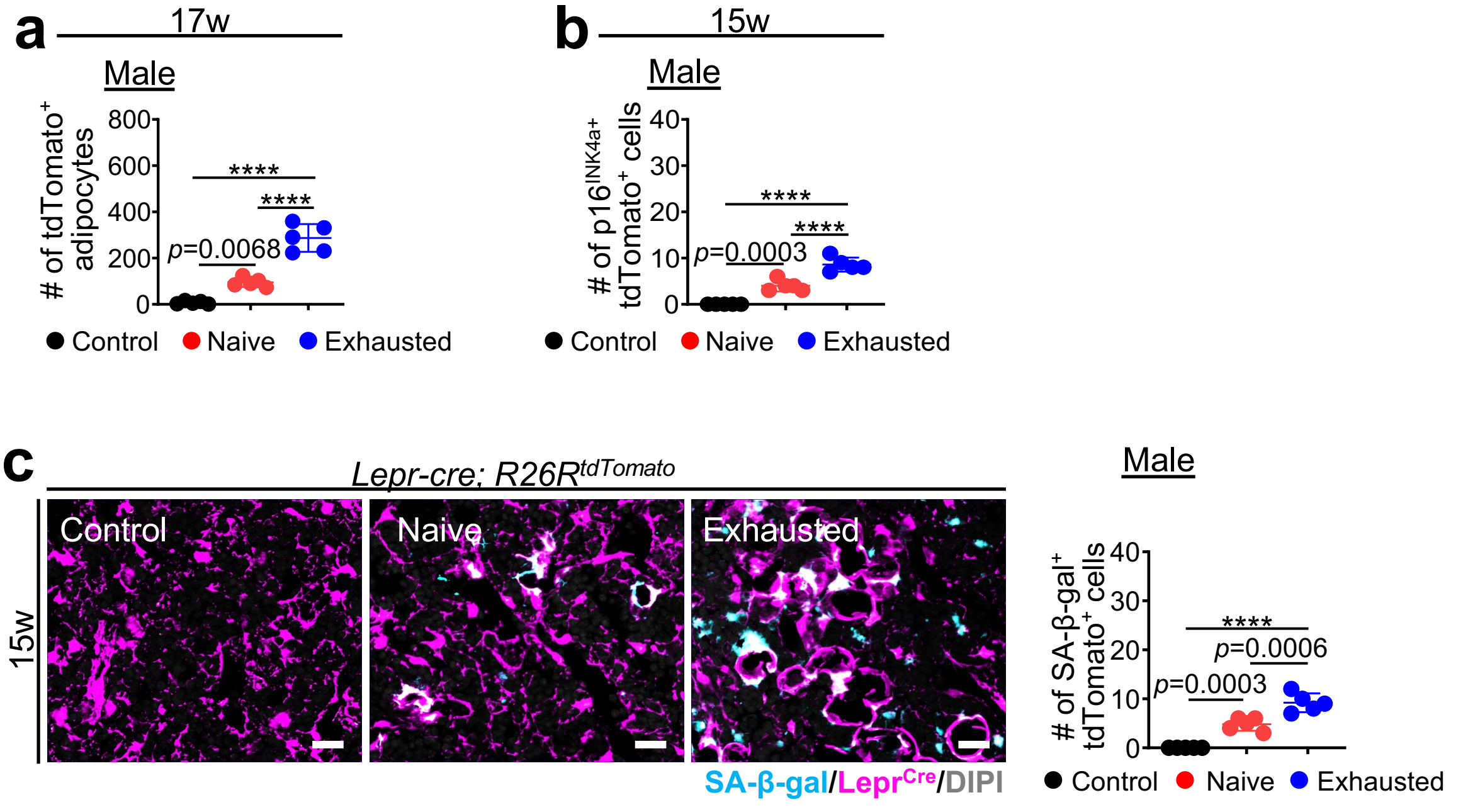

Supplementary Figure 5. Stromal exhaustion promotes adipogenic differentiation in male mice

(a) Quantification of tdTomato^{+} adipocytes in distal femurs from male *Lepr-cre; R26R^{tdTomato}* mice at 17 weeks of age. *n* = 5 mice per group.
(b) Quantification of p16^{INK4a+}tdTomato^{+} cells in male *Lepr-cre; R26R^{tdTomato}* mice at 15 weeks of age. *n* = 5 mice per group.
(c) Representative images and quantification of SA-β-gal^{+}tdTomato^{+} cells in distal femurs from male *Lepr-cre; R26R^{tdTomato}* mice at 15 weeks of age. Scale bars, 20 μm. *n* = 5 mice per group.
Exact P values are shown in the graphs; ****$p < 0.0001$. Two-tailed, One-way ANOVA followed by Tukey's post hoc test. Data are presented as mean ± S.D. Source data are provided as a Source Data file.

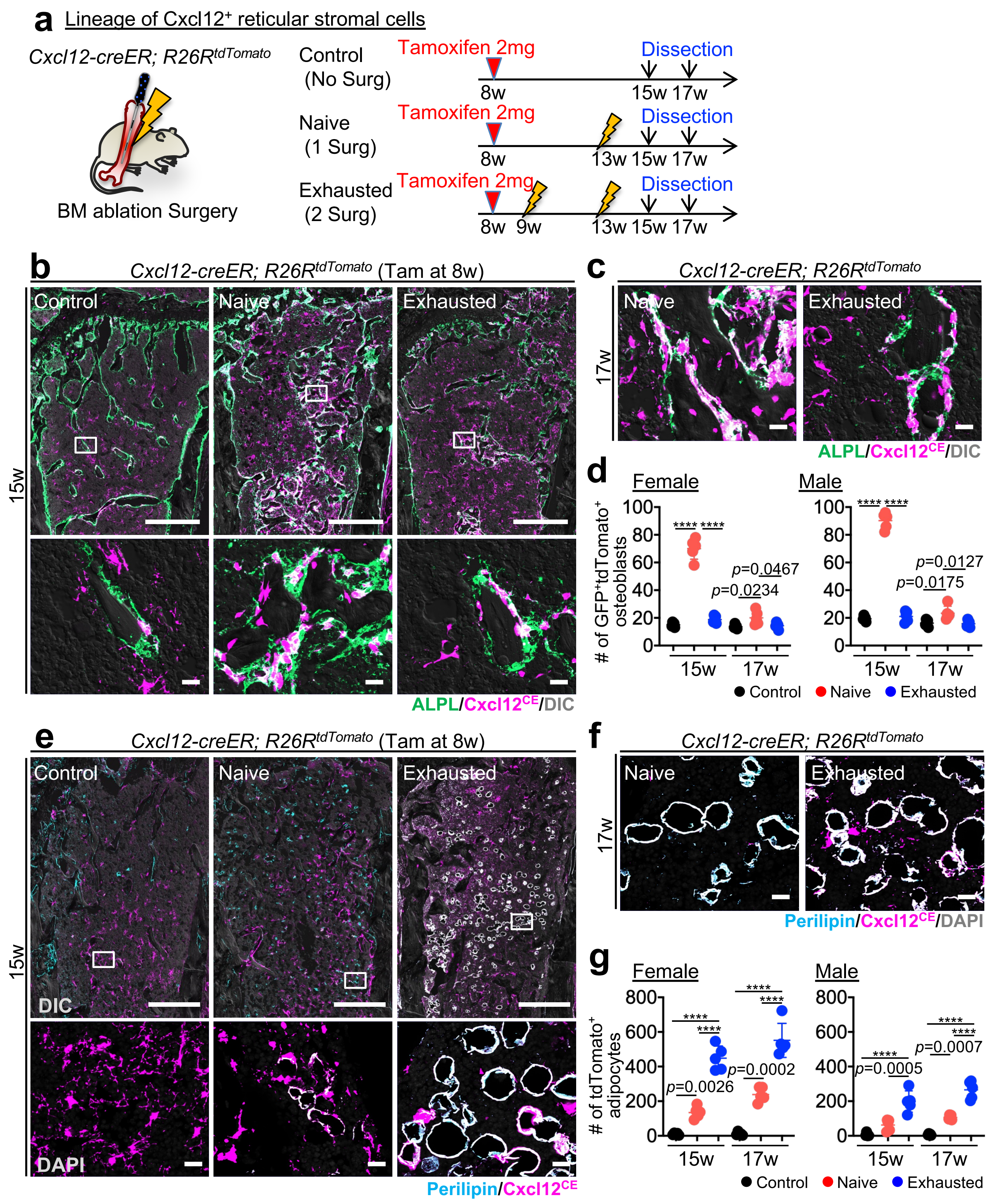

Supplementary Figure 6. Cxcl12-creER+ exhausted stromal cells lose osteogenic capacity and instead differentiate into adipocytes

(a) Experimental design illustrating the timeline of bone marrow ablation surgeries and analysis in *Cxcl12-creER; R26R^{tdTomato}* mice.

(b,c) *Cxcl12-creER; R26R^{tdTomato}* femurs with ALPL staining in control, healthy, and exhausted groups at 15 (b) and 17 (c) weeks of age. Tamoxifen injection at 8 weeks. Scale bar: 500 µm (upper left) and 20 µm (lower left and right).

(d) Quantitative analysis of the number of Cxcl12^{CE}-tdTomato^{+} osteoblasts at 15 and 17 weeks of age. *n* = 5 mice per each group.

(e,f) *Cxcl12-creER; R26R^{tdTomato}* femurs with Perilipin staining in control, healthy, and exhausted groups at 15 (e) and 17 (f) weeks of age. Tamoxifen injection at 8 weeks. Scale bar: 500 µm (upper left) and 20 µm (lower left and right).

(g) Quantitative analysis of the number of Cxcl12^{CE}-tdTomato^{+} adipocytes at 15 and 17 weeks of age. *n* = 5 mice per each group. Exact P values are shown in the graphs; ****$p < 0.0001$. Two-tailed, One-way ANOVA followed by Tukey's post hoc test. Data are presented as mean ± S.D. Source data are provided as a Source Data file.

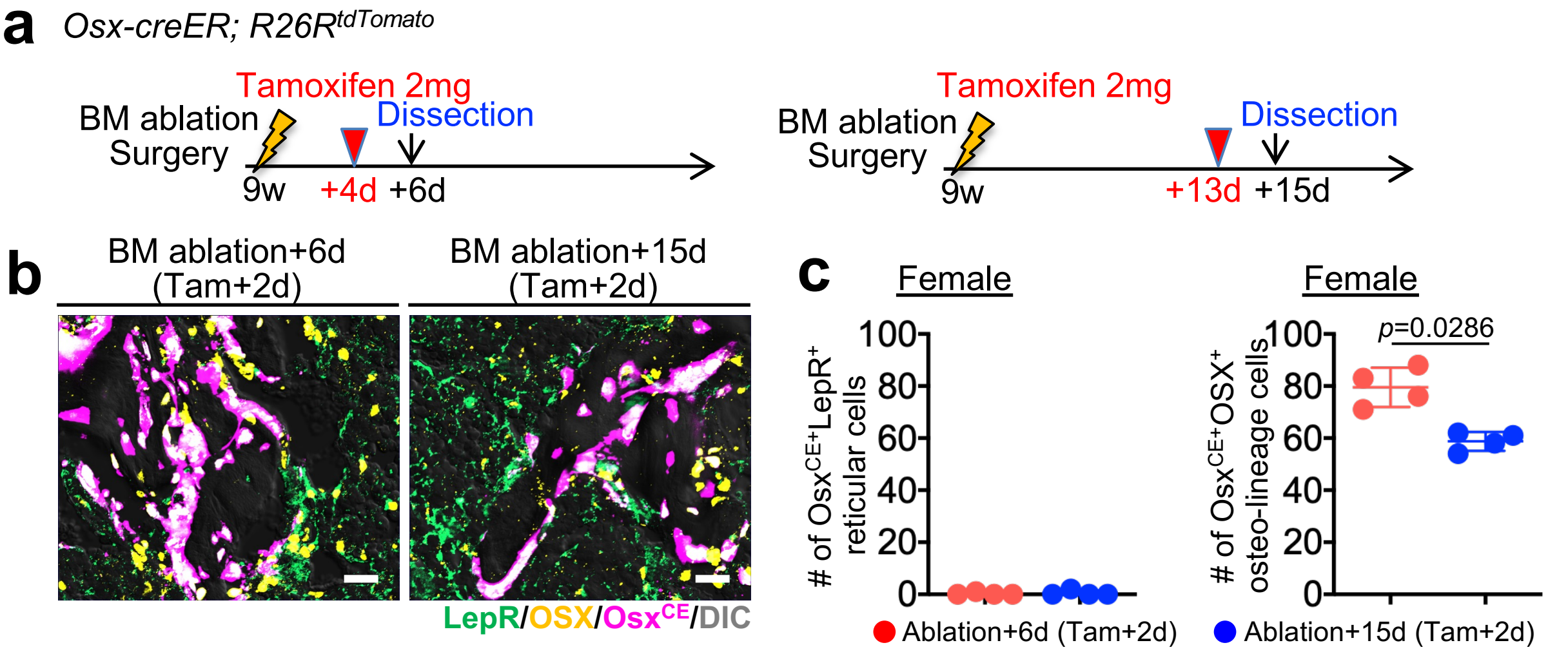

Supplementary Figure 7. Short-term lineage-traced Osx^{CE+} cells show minimal overlap with LepR^{+} stromal cells

(a) Experimental design for short-term lineage tracing in *Osx-creER; R26R^{tdTomato}* mice after bone marrow ablation. Tamoxifen was administered 4 or 13 days after surgery, and femurs were analyzed 2 days later, at 6 or 15 days after surgery, respectively.
(b) Representative images of femurs from mice analyzed 2 days after tamoxifen administration at day 4 or day 13 after bone marrow ablation. Scale bars, 20 µm.
(c) Quantification of tdTomato^{+}LepR^{+} reticular stromal-like cells and tdTomato^{+}OSX^{+} osteoblast-lineage cells in female mice. $n = 4$ mice per group.
Exact P values are shown in the graphs. Two-tailed, Mann–Whitney U test. Data are presented as mean ± S.D. Source data are provided as a Source Data file.

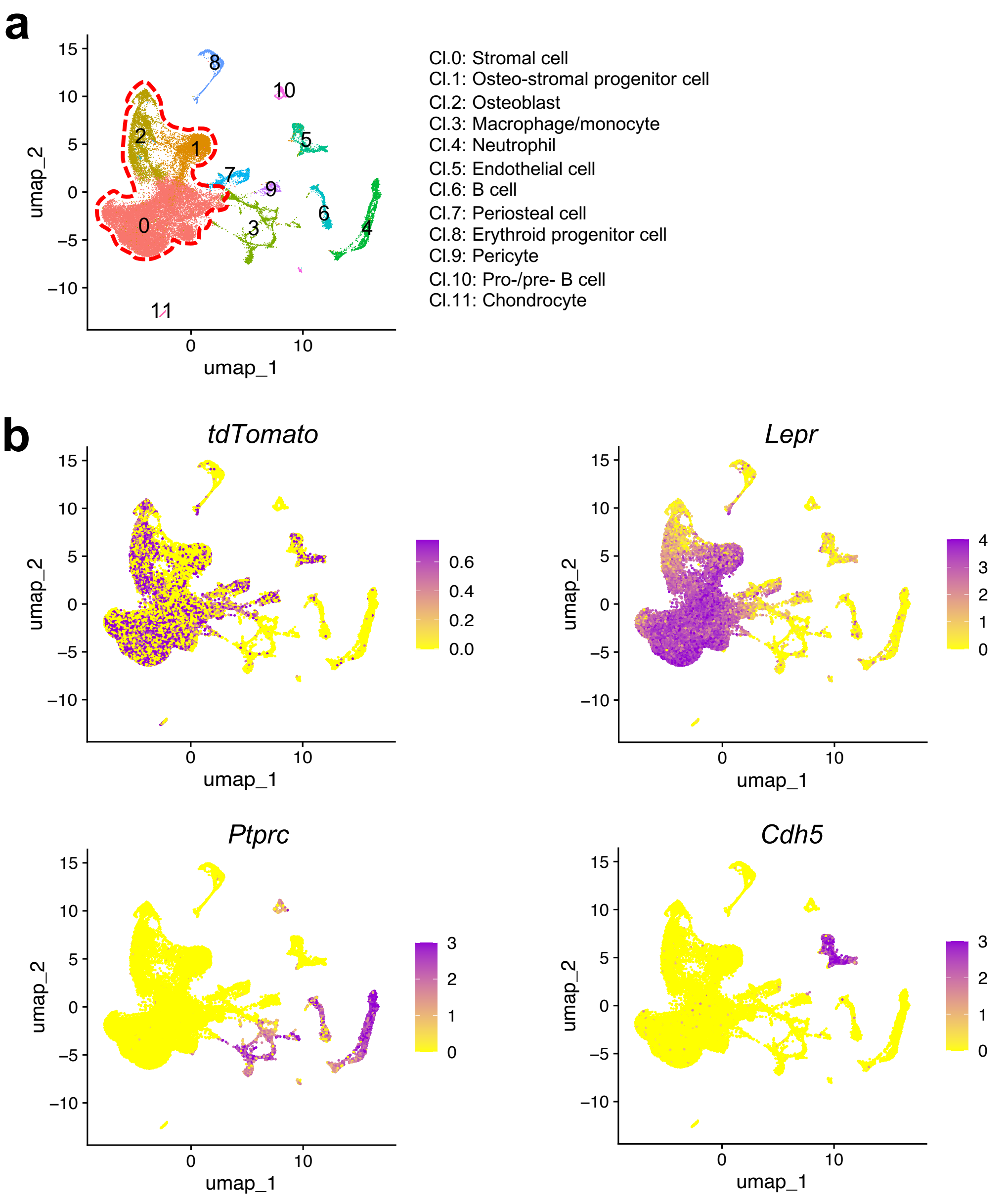

Supplementary Figure 8. Single-cell transcriptomic profiling

(a) UMAP visualization of major clusters (Clusters 0–11) of integrated datasets, with tdTomato^{+} skeletal cell populations inside the bone highlighted (red dashed outline).
(b) Feature plots showing expression of canonical markers used for cluster annotation: *tdTomato, Lepr* (Lepr^{Cre}-tdTomato^{+} lineage cell), *Ptprc* (hematopoietic cell), *Cdh5* (vascular cell).

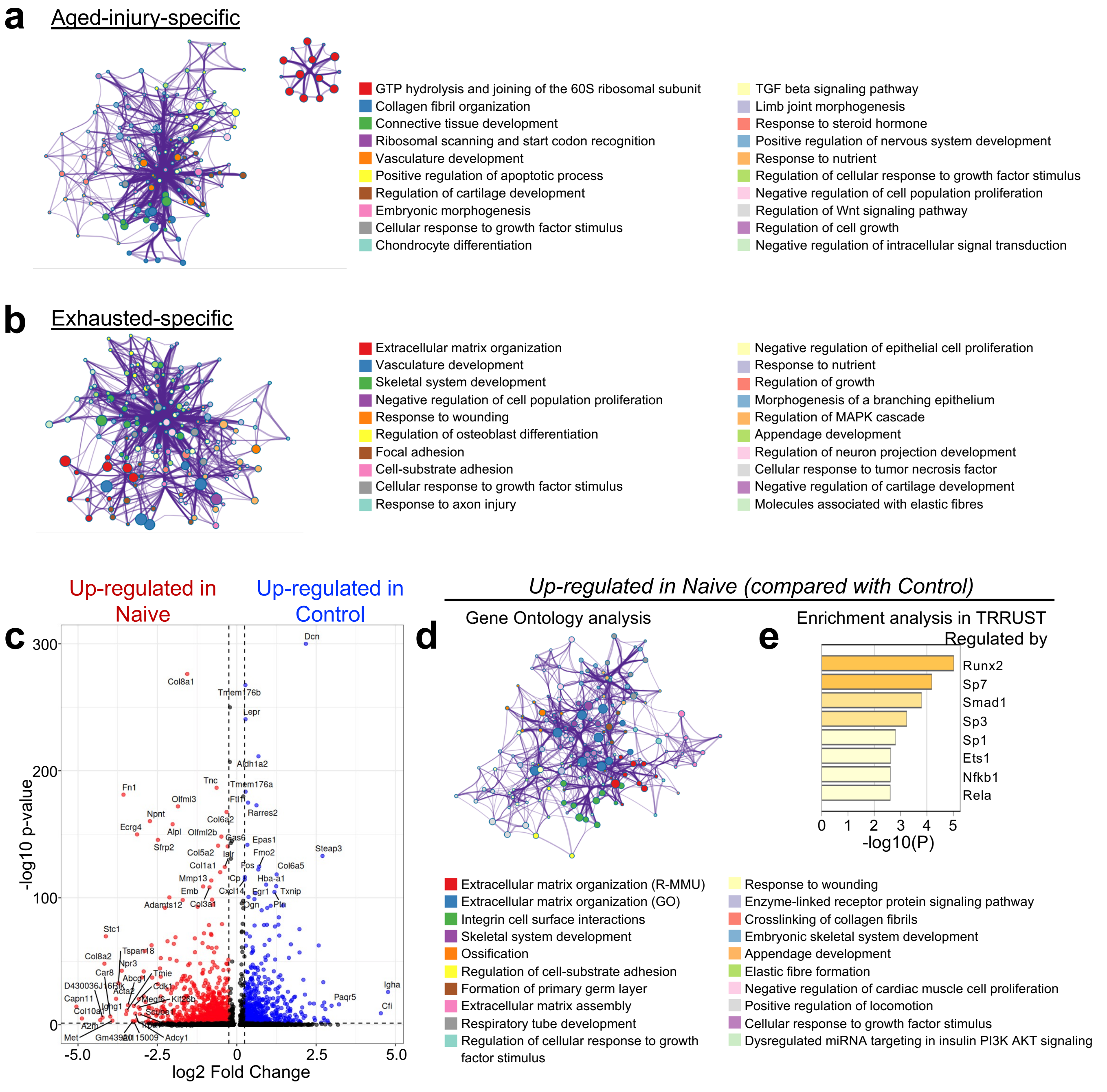

Supplementary Figure 9. Comparative pathway features of stromal cell states

(a) Gene-ontology network of pathways enriched among genes upregulated in aged-injury stroma.
(b) Gene-ontology network of pathways enriched among genes upregulated in exhausted stroma.
(c) Volcano plot of differential gene expressions between naive and control stromal cells. Upregulated genes in naive stroma (red) and control stroma (blue).
(d) Gene-ontology network of pathways enriched among genes upregulated in naive stroma.
(e) Transcription-factor enrichment analysis for naive-upregulated genes, based on the TRRUST database.

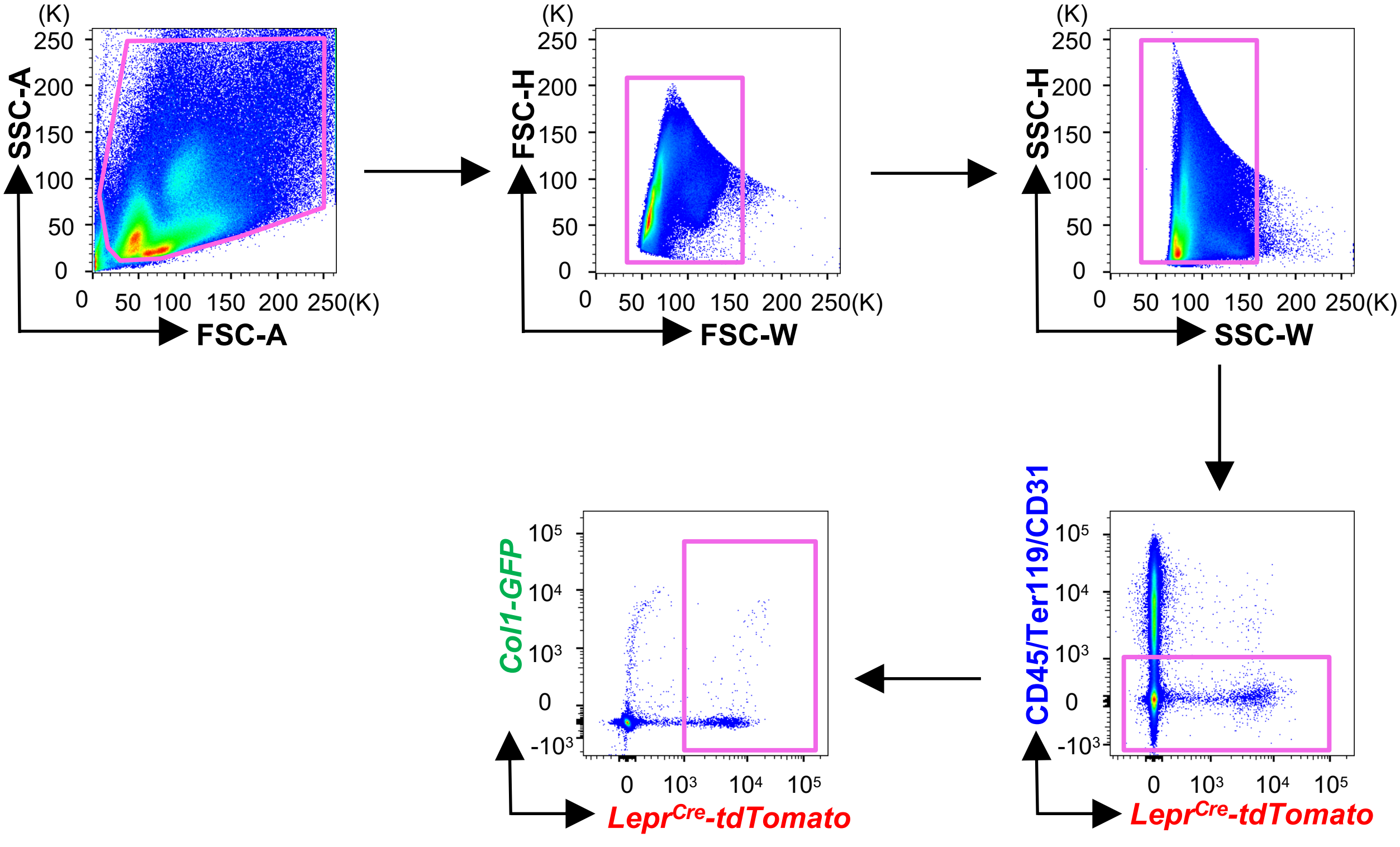

Supplementary Figure 10. Gating strategy for sorting Lepr^{Cre}-tdTomato^{+} cells

Cell sorting strategy of Lepr^{Cre}-tdTomato^{+} cells isolated from *Col1a1(2.3kb)-GFP*; *Lepr-cre*; *R26R*^{tdTomato} femurs. Isolated Lepr^{Cre}-tdTomato^{+} cells in control, naive, exhausted, aged, and aged-injury groups were used for scRNA-seq analysis.
