# Supplementary material for: Injury-driven stromal exhaustion disrupts intrinsic regenerative capability
Source: Nat Commun. 2026 Aug 19;17:8259. doi: 10.1038/s41467-026-76108-z (PMC13490406; doi:10.1038/s41467-026-76108-z)
Supplement: Supplementary file 2 [file 41467_2026_76108_MOESM2_ESM.pdf]

nature portfolio

Corresponding author(s): Yuki Matsushita

Last updated by author(s): Jun 23, 2026

Reporting Summary

Nature Portfolio wishes to improve the reproducibility of the work that we publish. This form provides structure for consistency and transparency in reporting. For further information on Nature Portfolio policies, see our Editorial Policies and the Editorial Policy Checklist.

Statistics

For all statistical analyses, confirm that the following items are present in the figure legend, table legend, main text, or Methods section.

n/a	Confirmed	
☐	☒	The exact sample size (*n*) for each experimental group/condition, given as a discrete number and unit of measurement
☐	☒	A statement on whether measurements were taken from distinct samples or whether the same sample was measured repeatedly
☐	☒	The statistical test(s) used AND whether they are one- or two-sided<br>*Only common tests should be described solely by name; describe more complex techniques in the Methods section.*
☐	☒	A description of all covariates tested
☐	☒	A description of any assumptions or corrections, such as tests of normality and adjustment for multiple comparisons
☐	☒	A full description of the statistical parameters including central tendency (e.g. means) or other basic estimates (e.g. regression coefficient) AND variation (e.g. standard deviation) or associated estimates of uncertainty (e.g. confidence intervals)
☐	☒	For null hypothesis testing, the test statistic (e.g. *F*, *t*, *r*) with confidence intervals, effect sizes, degrees of freedom and *P* value noted<br>*Give P values as exact values whenever suitable.*
☒	☐	For Bayesian analysis, information on the choice of priors and Markov chain Monte Carlo settings
☒	☐	For hierarchical and complex designs, identification of the appropriate level for tests and full reporting of outcomes
☒	☐	Estimates of effect sizes (e.g. Cohen's *d*, Pearson's *r*), indicating how they were calculated

Our web collection on statistics for biologists contains articles on many of the points above.

Software and code

Policy information about availability of computer code

Data collection	ZEN 3 pro (blue edition, ZEISS), Rigaku R_mCT micro-CT system, Bruker SkyScan 1272 micro-CT system, BD FACSAria II cell sorter (BD Biosciences), LightCycler 480 software (Roche), BD Rhapsody WTA Analysis Pipeline v2.3 on Seven Bridges, NovaSeq X Plus software (Illumina)
Data analysis	TRI-3D BON v.R9.02.00.0-H-64 (Ratoc Systems), ZEN 3 pro (blue edition, ZEISS), FlowJo (TreeStar), GraphPad Prism, BD Rhapsody WTA Analysis Pipeline v2.3 on Seven Bridges, Seurat v5.2.1, Metascape, TRRUST, ChIP-Atlas

For manuscripts utilizing custom algorithms or software that are central to the research but not yet described in published literature, software must be made available to editors and reviewers. We strongly encourage code deposition in a community repository (e.g. GitHub). See the Nature Portfolio guidelines for submitting code & software for further information.

Data

Policy information about availability of data

All manuscripts must include a data availability statement. This statement should provide the following information, where applicable:

- Accession codes, unique identifiers, or web links for publicly available datasets
- A description of any restrictions on data availability
- For clinical datasets or third party data, please ensure that the statement adheres to our policy

Source data are provided with this paper. The single-cell RNA-seq data generated and analyzed in this study have been deposited in the NCBI Gene Expression Omnibus (GEO) under SuperSeries accession number GSE302366, including GSM9102740-GSM9102743 and GSM9744609. Public scRNA-seq datasets GSE136970

and GSE136979 were also reanalyzed. The scRNA-seq data were processed using the BD Rhapsody WTA Analysis Pipeline v2.3 on Seven Bridges Genomics with the mouse reference genome GRCm39 (Release M32). Other source data for figures are available from the Lead Contact upon reasonable request.

Research involving human participants, their data, or biological material

Policy information about studies with human participants or human data. See also policy information about sex, gender (identity/presentation), and sexual orientation and race, ethnicity and racism.

Reporting on sex and gender	This study did not involve human participants or human data; therefore, sex and gender reporting for human participants is not relevant to this study.
Reporting on race, ethnicity, or other socially relevant groupings	This study did not involve human participants or human data; therefore, race, ethnicity, or other socially relevant groupings were not collected or analyzed.
Population characteristics	This study did not involve human participants, human data, or human biological material.
Recruitment	No human participants were recruited for this study.
Ethics oversight	This study did not involve human participants, human data, or human biological material; human research ethics approval was therefore not required.

Note that full information on the approval of the study protocol must also be provided in the manuscript.

Field-specific reporting

Please select the one below that is the best fit for your research. If you are not sure, read the appropriate sections before making your selection.

☒ Life sciences ☐ Behavioural & social sciences ☐ Ecological, evolutionary & environmental sciences

For a reference copy of the document with all sections, see nature.com/documents/nr-reporting-summary-flat.pdf

Life sciences study design

All studies must disclose on these points even when the disclosure is negative.

Sample size	No statistical method was used to predetermine sample size. We chose the numbers of mice based on the similar research in the field (NatCommun 11:332, 2020, NatCommun 13:4166, 2022). These sample size give good standard errors of the mean and good statistics to make it unlikely that we miss a biologically important difference between groups.
Data exclusions	Some of the data were excluded from the study because of the pre-established criteria such as problems or failures in identifying correct genotypes or birth dates, and issues unrelated to the intervention of the study such as spontaneous malnutrition. In any case, we consistently used littermate controls with corresponding genotypes in analysis.
Replication	For most quantitative experiments, at least four independent biological samples were examined, unless otherwise stated in the figure legends. Representative imaging data were obtained from at least four independent biological samples unless otherwise indicated. All attempts at replication were successful.
Randomization	The experiments were not randomized. We used all the available mice of the desired genotypes. Mice were allocated to particular groups based on results of PCR-genotyping typically performed around one week after birth. Covariates were controlled by considering multiple factors, such as genotypes and general phenotypical data (i.e. body weight). On principle, we did not observe any particular difference among groups.
Blinding	The investigators were not blinded to allocation during experiments and outcome assessment because it was impossible due to following reasons: samples were allocated to particular groups before experiments were initiated based on genotyping results, and given unique identifiers highlighting groups throughout experiments i.e. housing in cages,tissue collections, sample preparation and data acquisition. However, we did not pay particular attention to groups when we were measuring and counting.

Reporting for specific materials, systems and methods

We require information from authors about some types of materials, experimental systems and methods used in many studies. Here, indicate whether each material, system or method listed is relevant to your study. If you are not sure if a list item applies to your research, read the appropriate section before selecting a response.

Materials & experimental systems

n/a	Involved in the study
	☒ Antibodies
☒	Eukaryotic cell lines
☒	Palaeontology and archaeology
	☒ Animals and other organisms
☒	Clinical data
☒	Dual use research of concern
☒	Plants

Methods

n/a	Involved in the study
☒	ChIP-seq
	☒ Flow cytometry
☒	MRI-based neuroimaging

Antibodies

Antibodies used

Sigma
Rabbit anti-Perilipin A/B polyclonal antibody (Cat# P1873, 1:200)

R&D Systems
Goat anti-ALPL polyclonal antibody (Cat# AF2910, 1:200)
Goat anti-LepR polyclonal antibody (Cat# AF497, 1:200)

Abcam
Rat anti-CDKN2A/p16INK4a monoclonal antibody (Cat# ab241543, 1:200)
Rabbit anti-Cathepsin K monoclonal antibody (Cat# ab300569, 1:200)
Rabbit anti-Osterix polyclonal antibody (Cat# ab22552, 1:200)
Rabbit anti-beta-Catenin polyclonal antibody (Cat# ab16051, 1:200)

Cell Signaling Technology
Rabbit anti-Cleaved Caspase-3 polyclonal antibody (Cat# 9661, 1:200)

Thermo Fisher/Invitrogen
Alexa Fluor 633 goat anti-rabbit IgG (Cat# A21071, 1:400)
Alexa Fluor 633 goat anti-rat IgG (Cat# A21094, 1:400)
Alexa Fluor 488 donkey anti-goat IgG (Cat# A11055, 1:400)
Alexa Fluor 488 donkey anti-rabbit IgG (Cat# A11034, 1:400)

Invitrogen/eBioscience
APC-conjugated anti-CD31 antibody, clone 390 (Cat# 17-0311-82, 1:500)
APC-conjugated anti-CD45 antibody, clone 30-F11 (Cat# 17-0451-82, 1:500)
APC-conjugated anti-Ter119 antibody, clone TER-119 (Cat# 17-5921-82, 1:500)

Validation

All antibodies used here are commercially available. More detailed information about these antibodies is available on the manufacturers' websites. Briefly, antibodies from Sigma, R&D Systems, Abcam, Cell Signaling Technology, Thermo Fisher/Invitrogen, and Invitrogen/eBioscience were used according to the manufacturers' recommended applications and dilutions. Antibody specificity was supported by the expected staining patterns in mouse femur sections and concordance with genetic lineage reporters or established cell-state markers, including Perilipin for marrow adipocytes, ALPL/OSX/Col1a1-GFP for osteogenic cells, LepR/LeprCre-tdTomato for reticular stromal lineage cells, Cathepsin K for osteoclasts, Cleaved Caspase-3 for apoptotic cells, β-Catenin for Wnt/β-catenin signaling, and p16INK4a/SA-β-gal for senescence-associated cells. Flow cytometry gates were defined by FSC/SSC, singlet discrimination, APC-lineage negative selection (CD31/CD45/Ter119), and tdTomato reporter positivity; the gating strategy is shown in Supplementary Fig. 10

Animals and other research organisms

Policy information about studies involving animals; ARRIVE guidelines recommended for reporting animal research, and Sex and Gender in Research

Laboratory animals

Genetically modified Mus musculus strains were used. Mouse lines included Cxcl12-creER, Osx-creER, p16-creER, Rosa26-CAG-loxP-stop-loxP-tdTomato (Ai14; R26R-tdTomato, JAX007914), Col1a1(2.3 kb)-GFP (JAX013134), Lepr-cre (JAX008320), Ctnnb1-floxed (JAX004152), Ctnnb1lox(ex3)/+, C57BL/6J (JAX000664), and BALB/cCrSlc mice. Cxcl12-creER and Osx-creER mice were previously described; p16-creER mice were provided by Makoto Nakanishi; Ctnnb1lox(ex3)/+ mice were provided by Makoto M. Taketo. Mice were analyzed in mixed or C57BL/6J backgrounds. Animals were housed under specific pathogen-free conditions in static microisolator cages with ad libitum food and water, 22-23 degrees C, 40-65% humidity, and a 12 h light/dark cycle. Most injury-regeneration experiments used mice between 9 and 17 weeks of age; aged cohorts used 18-month-old mice, and some steady-state analyses included 6-month-old mice.

Wild animals

No wild animals were used in this study.

Reporting on sex

Both male and female mice were used where indicated, and sex-specific quantification was shown in relevant experiments. Female mice from the same litter were used for cell sorting/scRNA-seq and for some genetic analyses where stated in the figure legends. No

sex-based exclusion was applied except when the experimental design or availability of appropriate littermate/genotype groups required a single sex.

Field-collected samples	No field-collected samples were used in this study.
Ethics oversight	All animal experiments were performed in accordance with protocols approved by the Animal Care and Use Committee of Nagasaki University (#2204261788 and #2404301942).

Note that full information on the approval of the study protocol must also be provided in the manuscript.

Plants

Seed stocks	No plants were used in this study.
Novel plant genotypes	No plants were used in this study.
Authentication	No plants were used in this study.

Flow Cytometry

Plots

Confirm that:

☒ The axis labels state the marker and fluorochrome used (e.g. CD4-FITC).

☒ The axis scales are clearly visible. Include numbers along axes only for bottom left plot of group (a 'group' is an analysis of identical markers).

☒ All plots are contour plots with outliers or pseudocolor plots.

☒ A numerical value for number of cells or percentage (with statistics) is provided.

Methodology

Sample preparation	Soft tissues and epiphyses were removed from dissected femurs. After removing distal epiphyseal growth plates and cutting proximal ends, femurs were cut and digested with 2 Wunsch units of Liberase TM and 1 mg Pronase in 2 ml Ca2+/Mg2+-free HBSS at 37 degrees C for 60 min with shaking. Cells were mechanically triturated with an 18-gauge needle and pestle/mortar, filtered through a 70 um cell strainer into tubes on ice, and collected as single-cell suspensions. Dissociated cells were stained with APC-conjugated antibodies against CD31, CD45 and Ter119 before sorting. Approximately 30,000 tdTomato+ cells were sorted into ice-cold DPBS/10% FBS and resuspended for downstream scRNA-seq or molecular analyses.
Instrument	Three-laser BD FACSAria II high-speed cell sorter (BD Biosciences).
Software	FlowJo software (TreeStar) was used for flow cytometry data analysis.
Cell population abundance	post-sort purity was not determined.
Gating strategy	Cells were first gated by FSC-A/SSC-A to exclude debris, followed by singlet discrimination using FSC-W/FSC-H and SSC-W/SSC-H. Lineage-negative cells were selected as CD31–CD45–Ter119–, and tdTomato+ cells were then gated for sorting. The full gating strategy is provided in Supplementary Fig. 10.

☒ Tick this box to confirm that a figure exemplifying the gating strategy is provided in the Supplementary Information.
