# Supplementary material for: Injury-driven stromal exhaustion disrupts intrinsic regenerative capability
Source: Nat Commun. 2026 Aug 19;17:8259. doi: 10.1038/s41467-026-76108-z (PMC13490406; doi:10.1038/s41467-026-76108-z)
Supplement: Supplementary file 3 [file 41467_2026_76108_MOESM3_ESM.pdf]

nature portfolio

Peer Review File

Injury-driven stromal exhaustion disrupts intrinsic regenerative capability

Corresponding Author: Professor Yuki Matsushita

Parts of this Peer Review File have been redacted as indicated to remove third-party material.

This file contains all reviewer reports in order by version, followed by all author rebuttals in order by version.

Version 0:

Reviewer comments:

Reviewer #1

(Remarks to the Author)
In this manuscript, the authors demonstrate that Cxcl12^{+} LepR^{+} bone marrow skeletal stem cells become dysfunctional following repeated injury, introducing a novel concept of “stromal exhaustion.”
This concept is important for understanding the pathogenesis of various skeletal disorders, including fractures, and may have broader implications for the field of stem cell biology.
The experiments are well-controlled, the results are clear, and the conclusions appear sound. This manuscript offers a conceptual advance that potentially meets the high criteria for publication in Nature Communications. However, there remains room for improvement.

1. The authors concluded that the “exhausted” state is distinct from the “aged” state without providing any functional validation. Since the concept of “aged skeletal stem cells” has already been reported (Ambrosi et al., Nature 2021), a more detailed comparative analysis including functional assays between “exhausted skeletal stem cells” and “aged skeletal stem cells” is required to substantiate the novel concept of stromal exhaustion.

2. Although the authors proposed impaired β-catenin signaling as the molecular mechanism responsible for stromal cell exhaustion, this hypothesis lacks sufficient experimental validation. The authors should examine whether pharmacological or genetic activation of β-catenin signaling can reverse the exhausted phenotype of Cxcl12^{+} LepR^{+} bone marrow skeletal stem cells.

3. Given that WNT/β-catenin signaling is essential for osteogenesis (Day et al., Dev Cell 2005, Hill et al., Dev Cell 2005, Chen et al., JBMR 2013), and LepR^{+} cells serve as a source of osteoblasts, it is somewhat surprising to this reviewer that Lepr-Ctnnb1 cKO mice exhibit normal bone volume under steady-state conditions (Fig.7b). The authors should explain why the deletion of β-catenin in LepR^{+} cells did not affect bone metabolism.

4. It is interesting that inflammatory pathways, including IL-17 signaling and the Toll-like receptor cascade, are enriched in exhausted stromal cells. The authors should discuss the possibility that the bone marrow immune system is involved in injury-induced stromal exhaustion.

Reviewer #2

(Remarks to the Author)
In this manuscript Wu et al. examine the outcomes and cellular basis of the response to repeated injury in the skeletal system, ultimately establishing that impaired healing after repeated injury correlates with the presence of an "exhausted" state in a specific populations of Cxcl12 abundant reticular cells (CAR cells). Overall, there is a high degree of enthusiasm for this study, as concepts of progenitor exhaustion after repeated insults and trained immunity have emerged as important areas of study in other organ systems yet have largely not been similarly establishing in the bone field. Thus, this manuscript is felt to represent an important conceptual advance. Overall, the study is felt to be sound in approach, with most of the comments below representing requests for clarification or to adjust the terminology used. The only major comments are that

this study would likely benefit from a bit more data to fully flesh out the relationship of this exhaustion state to sensenece.
Major points
1. The senescence field in bone now typically holds that a single marker is insufficient to confidently determine that cells are senescent. A combination of numerous markers is now required as in work by Khosla and colleagues. If the authors want to make senescence claims regarding the exhausted CAR cells, a similar combination (p21 in addition to the p16 here, SA beta gal, evidence of senescence associated secretory phenotypes (SASP) ect...). However, this reviewer wonders if it is correct or desirable to effectively equate the exhaustion phenotype with the senescence phenotype that has now become widely studied in bone. It appears likely from both first principles and perhaps also the data here showing that the exhaustion state is not equivalent to aging that exhaustion is a distinct phenomenon from senescence. In this respect, demonstrating the differences between exhaustion and senescence is perhaps more important for the impact of the manuscript than emphasizing the similarities. However, this is just speculation on the part of this reviewer, and the data may ultimately indicate that exhaustion is highly similar to senescence. Ultimately, the relationship of the exhaustion phenotype to senescence should be better fleshed out with additional data.
2. In Fig 7, 13 weeks may be a bit young to see a phenotype at steady state from gene deletion in CAR cells, as past literature generally shows that CAR cell phenotypes have an onset after 4-5 months of age due to CAR cells emerging postnatally and having a gradually increasing contribution to the osteoblast pool, with excellent examples of these dynamics being described in the authors' past work. Ideally, this would be clarified with additional later timepoints, but it would also be acceptable to just robustly qualify the negative baseline data in the text without additional experimental data.

Minor points
1. The authors' use of the term "stem cell" to refer to CXCL12 abundant reticular (CAR) cells is unexpected and perhaps not the most accurate choice of terminology, especially as it is the authors' own work (especially Matsushita et al. Nat Comms 2023, Fig 4, but also Mizuhashi et al. Nature 2018 and even the Osx-based lineage tracing data here and also in Ono et al. Nat Cell Bio 2014) that provides clear and direct evidence of populations that are upstream of the postnatal generation of CAR cells. Given that perhaps the most direct definition of a skeletal stem cell is a self-renewing population that sits at the apex of their differentiation hierarchy, this would preclude the use of stem cell terminology to refer to CAR cells. From this perspective, it is recommended to avoid stem cell terminology in this manuscript to avoid further confusion on this point in the literature. Simialrly, "loss of stemness" should be removed from the text of Fig 8, as none of the assays performed here are appropriate for assessing stemness, as serial transplantation or similar assays would be required.
2. Related to the point above, the choice in the introduction to present FGFR3+ endosteal cells and CAR cells as parallel populations is similarly unexpected, given that the authors' prior work demonstrate that FGFR3 cells are upstream of CAR cell production. This would appear to indicate that describing these two populations in a hierarchical manner is more appropriate than describing them as parallel.
3. Figure 1 feels somewhat disconnected from the rest of the manuscript, and it feels like the manuscript properly starts with Fig 2 as the main topic of exhaustion is addressed. Conversely, perhaps the data in Ext Fig 3 is very important to the main points of the manuscript and could be considered for placement in the main text figures. However, this is just an optional suggestion for consideration.
4. The point made in Fig 1e that CAR cells are bipotent for the production of osteoblasts and adipocytes is likely controversial given competing models that propose that distinct sets of CAR cells are responsible for adipogenesis versus osteogenesis. Perhaps it is better to not endorse the bipotency model given that this is not an issue directly addressed by the present manuscript. This point is also made in lines 203 and 204--the studies cited in that line to support the bipotency claim are excellent studies but do not substantively address whether it is a single cell type, especially a single clonal lineage of CAR cells, that produces both osteoblasts and adipocytes. This text should also be revised and cartoon model figures should be considered from the perspective that it is unclear if CAR cells represent a single population that is clonally bipotent to generate both osteoblasts and adipocytes as opposed to being a pool of multiple lineage committed progenitors with distinct subsets that predominantly mediate osteogenesis versus adipogenesis.
5. "Healthy" is probably not the best term to describe the comparator to the exhausted groups, as this appears to imply that the exhaustion state is a non-physiologic or "unhealthy" state. Exhaustion may represent an adaptive response in some cases. Perhaps "naive" or similar terminology would be more precise.
6. Line 177--it is not appropriate to term an in vitro CFU assay as informative regarding self-renewal. The CFU assay cannot discriminate proliferation occurring in concert with differentiation from true self-renewal.
7. On what basis is cluster 7, line 287 termed periosteal? Pdpn is not sufficient to identify periosteal populations.
8. Line 349--are Fos and Nfkbia really markers of chronic inflammation? These genes have clear baseline physiological functions outside of inflammatory contexts.
9. The results in Fig 2 would be easiest to interpret if it could be assumed that all of trabecular bone in the region of interest analyzed is newly formed after injury. A time=0 datapoint showing to what degree the trabecular bone in the region analyzed by uCT would be helpful. It is also requested that non-normalized versions of the uCT data in Fig 2c (the raw values, without dividing by the control group) be provided, especially for BV/TV.
10. While the trajectory analysis in Fig 5 is useful, some qualification that trajectory analysis is inherently speculative should be made in the text. The authors may optionally consider briefly covering in the discussion the lineage tracing data that corroborates the model proposed based on trajectory analysis.

Reviewer #3

(Remarks to the Author)
The manuscript by Wu et al. explores the intriguing concept of stromal exhaustion in response to repeated skeletal injury as a potential root cause for impaired skeletal health and fragility. The authors conclude that mechanical injury impairs reticular cell function in the bone marrow preventing reversal to stromal cell states needed for injury responses. This is a well presented and written manuscript that uses male and female mice. However, the main claims of reversal of stroma-like cells, senescence, and exhaustion are not convincingly supported by provided data. Please refer to my specific comments below:
- ll.87 – 91: The authors mention endosteal and BM resident cells but not periosteal cells. For example, cited study by the Morisson group concluded contributions of periosteal cells. Please update that statement.
- Figure 1b: Please annotate clusters. Please also provide individual plots for control and injured clustering to better assess overlap and differences.
- Figure 1d: Please provide dotplots for a broader set of markers. It seems quite selective as is. Is this for all cells or just some clusters?
- Extended data Fig.2: It appears the contribution of Lepr+ cells to total CFU-F is quite low. Are those Lepr- cells growth plate or periosteal cell "contaminations"?
- Figure 2d suggests that there is more Lepr cell activity in periosteal areas in healthy group. Why would periosteal cells increase CFU-F in response to marrow ablation? Is the procedure consistent and highly reproducible?
- Figure 2 & Extended Data Fig.3: Overall, the authors mostly compare healthy with exhausted groups. It seems the exhausted phenotype more closely resembles the control group. It is unclear why this would suggest an impaired state. Do the increased BMAT fraction subside if bones were analyzed and compared at 20wks or longer after injury? It could be that the exhausted group might need additional times to recover. Adipocytes are known to fill the initial void left by injury/ablation but can resolve. Do the authors observe the same after multiple bi-cortical fractures?
- Figure 3 and Extended Data Fig 3e-g: It appears from images that the majority of BMAT is not Cxcl12-dTomato derived. Please provide individual fluorescence channels. Are those CAR cell derived adipocytes generated from SSC-generated stromal cells after Tamoxifen induction? Is that why images look like there is a significant difference in adipocyte number between Control and Healthy groups but the Fig.3c quantifications on tomato+ adipocytes does not show any difference (at least female looks significant)?
- p16INK4a by itself (and the expression of Cdkn2a and Cdkn1a) is not sufficient to claim senescent state (https://www.cell.com/cell/fulltext/S0092-8674(24)00640-8). Upregulation of these markers could just reflect an "emergency" state activated for proper resolution of injury. Additional assays should be performed, e.g., IHC for other markers and beta-Gal stain for stromal cells.
- Figure 4: The experimental setup does not allow for conclusions about reversion to stromal state as this can only be done if individually cells/clones were traced. Stromal fraction likely increases rather than OSX+ cells reverting. Reduction of OSX cells might also be driven by apoptosis. The conclusion is therefore not warranted and should be revised or demonstrated with additional experiments.
- Fig4G suggests that mainly Adipocytes are labeled by p16INK4a, but not other LepR cells, which would not align with the authors conclusion that stromal cells become senescent.
- Fig 5: it is unlikely that the 10X scRNAseq assays can capture mature adipocytes (due to size and processing as discussed by authors). Cluster should be renamed into adipogenic or adipocytic in Fig.5c. Similarly, Clusters 5 and 6 might contain Osteo-CAR cells (CXCL12 low) and should be identified as such. Pie charts for differences in cluster abundance (individually) should be shown in Fig 5e.
- RNA velocity/Cell Rank data is speculative and does not account for different lineages within entire cell pool analyzed. What is the "Skeletal Progenitor" cluster – shouldn't stromal cells also be considered skeletal progenitors if they generate osteo cells? Similarly, one could interpret results as if only periosteal cell contribute to adipogenic cells which does not align with the authors conclusions.
- Fig 5i: Please include all four groups in dot plot analysis for transparency. What about changes in hematopoiesis support which is crucial for regeneration after ablation procedures? The common marker expression between exhaustion and aged groups are also quite limited and selective.
- Fig.6: Again, Control group should be included here. Are these changes also seen in exhausted compared to untreated control? In 6g authors show control group data, which seems to resemble exhausted group. If these differences are just seen when compared to healthy, this could also imply that the one time ablation triggers a strong response, but not necessarily a "better" response. Would the same be observed if second injury/ablation was done 2-4 weeks later?
- Furthermore in Figure 6, the average expression is displayed as one color (not range as indicated in scale bar), which might be due to the fact that there are just two groups compared (on or off) making it hard to gauge how different expression differences are.
- Fig.7: Given the widely known role of Wnt signaling in osteogenesis it is not surprising or new that ablation of Ctnnb1 would have the observed effect. The connection to potential stromal exhaustion remains weak. This model also does not allow distinction between changes at stromal vs more mature osteoblast level in response to Ctnnb1 ablation. There is also no data inquiring which distinct source (location) of stromal progenitors are affected. LepR expression is extremely broad, and the investigation of more defined subsets (multi marker) would have provided more novelty.
- It is unclear where the "metabolic imbalance" in the title is derived from

Version 1:

Reviewer comments:

Reviewer #1

(Remarks to the Author)
I appreciate the authors' thoughtful responses to my previous comments. The addition of new experimental data has substantially strengthened the manuscript and provides further support for the proposed hypothesis and overall conceptual framework.
I have no further comments and congratulate the authors on this excellent study.

Reviewer #2

(Remarks to the Author)
The authors have robustly addressed all of the comments on the initial manuscript. The revised manuscript is felt to represent an important conceptual advance for the field by establishing cellular exhaustion as a new state of cellular dysfunction impacting bone formation and repair.

Reviewer #3

(Remarks to the Author)
The authors did an excellent job addressing all reviewer comments.

Open Access This Peer Review File is licensed under a Creative Commons Attribution 4.0 International License, which permits use, sharing, adaptation, distribution and reproduction in any medium or format, as long as you give appropriate credit to the original author(s) and the source, provide a link to the Creative Commons license, and indicate if changes were made.
In cases where reviewers are anonymous, credit should be given to 'Anonymous Referee' and the source.
The images or other third party material in this Peer Review File are included in the article's Creative Commons license, unless indicated otherwise in a credit line to the material. If material is not included in the article's Creative Commons license and your intended use is not permitted by statutory regulation or exceeds the permitted use, you will need to obtain permission directly from the copyright holder.
To view a copy of this license, visit https://creativecommons.org/licenses/by/4.0/

Response to Reviewer #1

Thank you very much for your constructive comments and critiques.

0. <Reviewer>

In this manuscript, the authors demonstrate that $Cxcl12^{+}$ $LepR^{+}$ bone marrow skeletal stem cells become dysfunctional following repeated injury, introducing a novel concept of "stromal exhaustion." This concept is important for understanding the pathogenesis of various skeletal disorders, including fractures, and may have broader implications for the field of stem cell biology.
The experiments are well-controlled, the results are clear, and the conclusions appear sound. This manuscript offers a conceptual advance that potentially meets the high criteria for publication in Nature Communications. However, there remains room for improvement.

<Response>

Thank you very much for your positive assessment on our study.

1. <Reviewer>

The authors concluded that the "exhausted" state is distinct from the "aged" state without providing any functional validation. Since the concept of "aged skeletal stem cells" has already been reported (Ambrosi et al., Nature 2021), a more detailed comparative analysis including functional assays between "exhausted skeletal stem cells" and "aged skeletal stem cells" is required to substantiate the novel concept of stromal exhaustion.

<Response>

Thank you for this important comment. We agree that directly distinguishing stromal exhaustion from physiological aging is essential to substantiate the novelty of our concept. We also acknowledge that age-related changes in skeletal stem cells have been well described, including in the study by Ambrosi et al. (Nature 2021), and that this provides important context for interpreting our findings. At the same time, our study focuses on bone marrow reticular stromal cells (CAR cells), which represent a distinct stromal population rather than skeletal stem cells broadly defined by cell surface markers and ex vivo function.

To address this point, we performed bone marrow ablation surgery in 18-month-old mice, generating an aged-injury group, and analyzed the regenerative response histologically. Although regenerative capacity was modestly reduced in aged mice, an injury-induced regenerative response was still clearly preserved. Importantly, new bone formation was observed after injury, and $Lepr\text{-}cre^{+}$ stromal cells retained the ability to differentiate into osteoblasts. These data have been included as revised **Figure 6**.

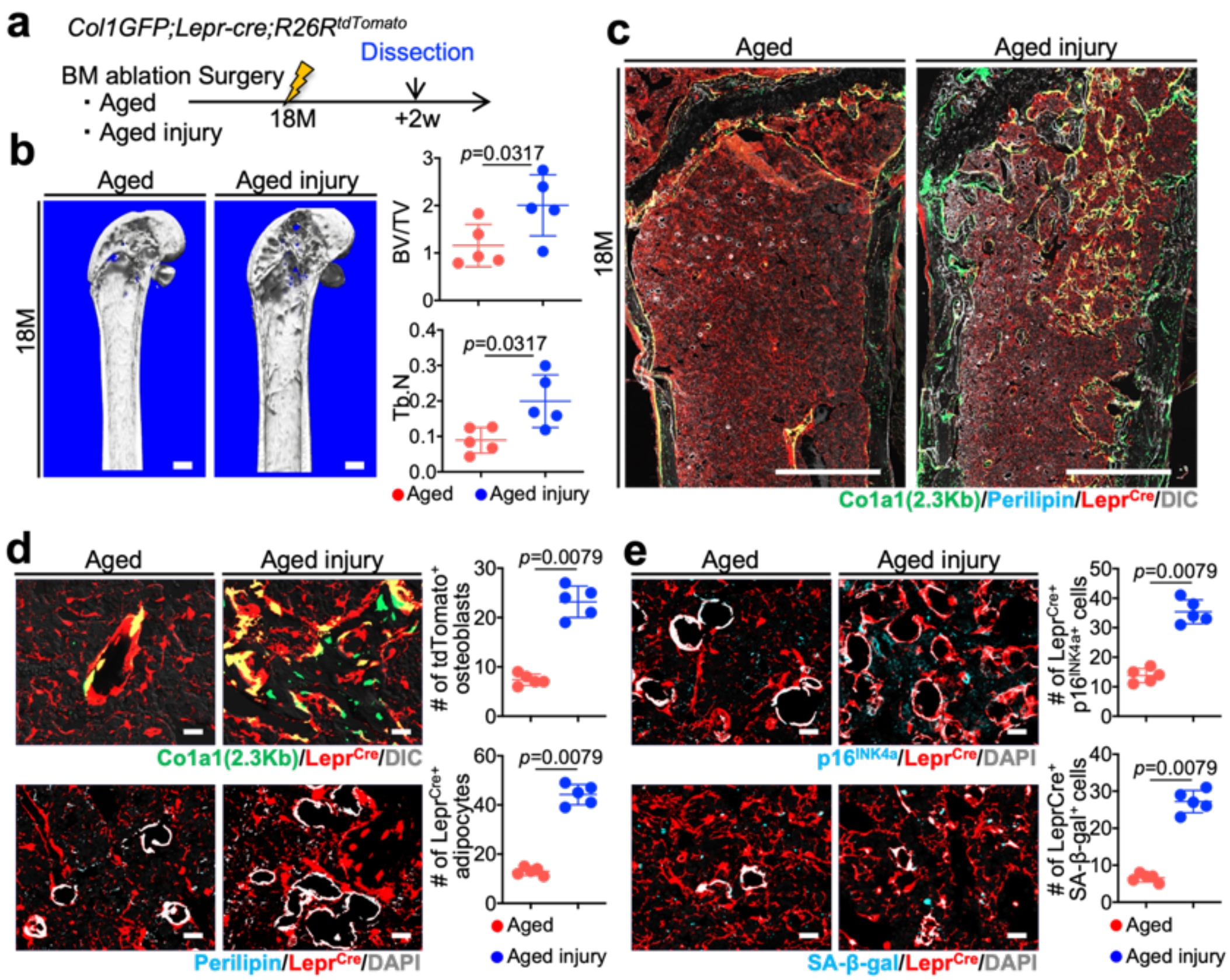

To further investigate this phenomenon at the molecular level, we performed scRNA-seq analysis of stromal populations in the aged-injury condition. Pathway enrichment analysis revealed that aged-injury stroma was characterized by activation of regenerative programs, including Wnt signaling, TGF-β signaling, chondrocyte differentiation, connective tissue development, and vasculature development. In contrast, exhausted stroma showed enrichment of inflammatory and stress-associated pathways, including TNF signaling, NF-κB/STAT3-related responses, and extracellular matrix remodeling. These results indicate that aged stromal cells maintain coordinated injury-responsive regenerative programs, whereas exhausted stroma fails to properly reactivate these pathways after repeated injury. These data have been included as revised **Extended Data figures 8a,b**.

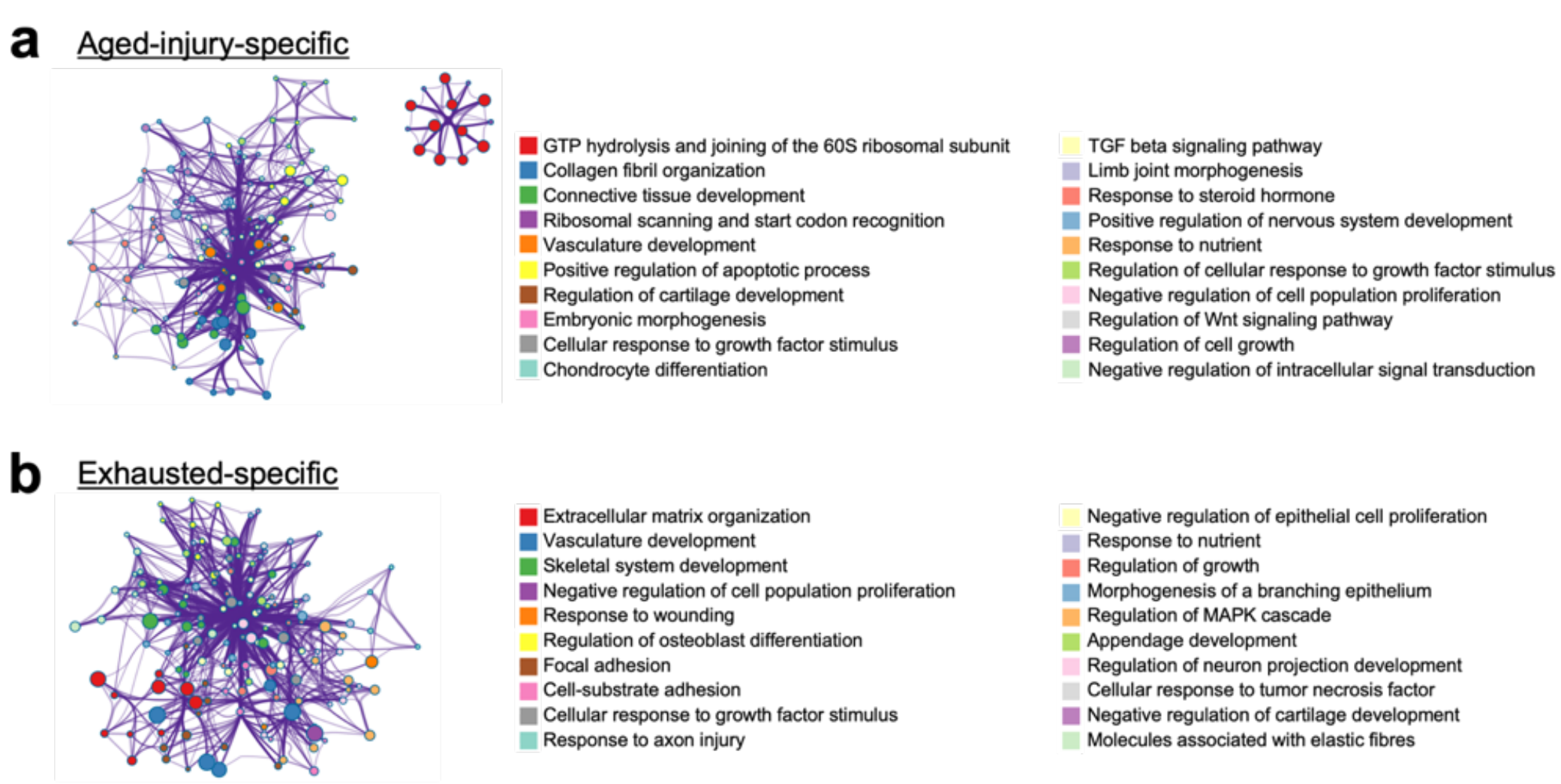

Together, these results demonstrate that stromal exhaustion is not simply an extension of physiological aging, but rather represents a distinct state characterized by loss of regenerative competence. Thus, our findings functionally and molecularly distinguish exhausted stromal cells from aged stromal populations. Importantly, while prior studies have defined age-associated dysfunction in skeletal stem cells, our data indicate that stromal exhaustion represents a distinct pathological state of CAR cells after repeated injury, rather than a direct extension of skeletal stem cell aging.

The following sentences have been added to the main text:

Line 328-344:

7. Stromal exhaustion is distinct from physiological aging

To determine whether stromal exhaustion simply reflects physiological aging of bone marrow stromal cells, we examined the regenerative response of aged stromal cells after injury. We performed bone marrow ablation surgery in 18-month-old Lepr-cre; R26RtdTomato mice and compared their response with that of no-surgery controls (Fig. 6a). Micro-CT analysis revealed that significant trabecular bone formation still occurred after injury (Fig. 6b). Histological analysis further showed that Lepr-cre lineage cells in aged mice retained the ability to contribute to osteoblasts after injury, whereas adipocyte accumulation was limited in aged mice under steady-state conditions and increased only modestly after injury (Fig. 6c,d). We also examined senescence-associated changes and found that the aged group showed only limited senescence-associated signals under steady-state conditions, although these signals increased after injury (Fig. 6e). Together, these results suggest that aged stromal cells retain injury-induced regenerative capacity, despite partial reductions in osteogenesis and increases in adipogenesis or senescence-associated features. Thus, stromal exhaustion is distinct from physiological aging and represents an injury-induced loss of regenerative capacity rather than a simple age-associated decline.

Line 392-401:

To further distinguish stromal exhaustion from physiological aging, we compared exhausted stroma with aged-injury stroma (Extended Data Fig. 8a,b). Pathway enrichment analysis revealed that aged-injury stroma retained injury-responsive regenerative programs, including Wnt signaling, TGF-β signaling, connective tissue development, and vasculature development. In contrast, exhausted stroma showed enrichment of inflammatory and stress-associated pathways, including TNF signaling, NF-κB/STAT3-related responses, and extracellular matrix remodeling. These findings indicate that aged stromal cells maintain coordinated injury-responsive regenerative programs, whereas exhausted stroma fails to properly reactivate these pathways after repeated injury, further supporting stromal exhaustion as a distinct injury-induced state rather than a simple extension of physiological aging.

Line 538-543:

Consistent with previous reports describing functional decline of aged skeletal stem cells, aging-associated stromal populations in our study also showed reduced osteogenic capacity. However, our data suggest that injury-driven exhaustion of bone marrow reticular stromal cells represents a distinct state from physiological

skeletal cell aging, characterized by a more profound failure of injury-induced regenerative reactivation and enhanced adipogenic conversion after repeated injury.

2. <Reviewer>

Although the authors proposed impaired β-catenin signaling as the molecular mechanism responsible for stromal cell exhaustion, this hypothesis lacks sufficient experimental validation. The authors should examine whether pharmacological or genetic activation of β-catenin signaling can reverse the exhausted phenotype of Cxcl12^{+} LepR^{+} bone marrow skeletal stem cells.

<Response>

Thank you for this important comment. We agree that functional validation of β-catenin signaling is essential to support its role in stromal exhaustion. To address this point, we performed both pharmacological and genetic rescue experiments to determine whether activation of Wnt/β-catenin signaling could rescue key features of the exhausted phenotype. First, we administered CHIR-99021, a GSK-3 inhibitor that activates Wnt/β-catenin signaling, by daily injection starting two weeks before the second bone marrow ablation surgery and continuing until necropsy. CHIR-99021 treatment significantly restored osteogenesis and reduced adipogenic differentiation in the bone marrow following repeated injury. These data have been included as revised **Figures 10a-d**.

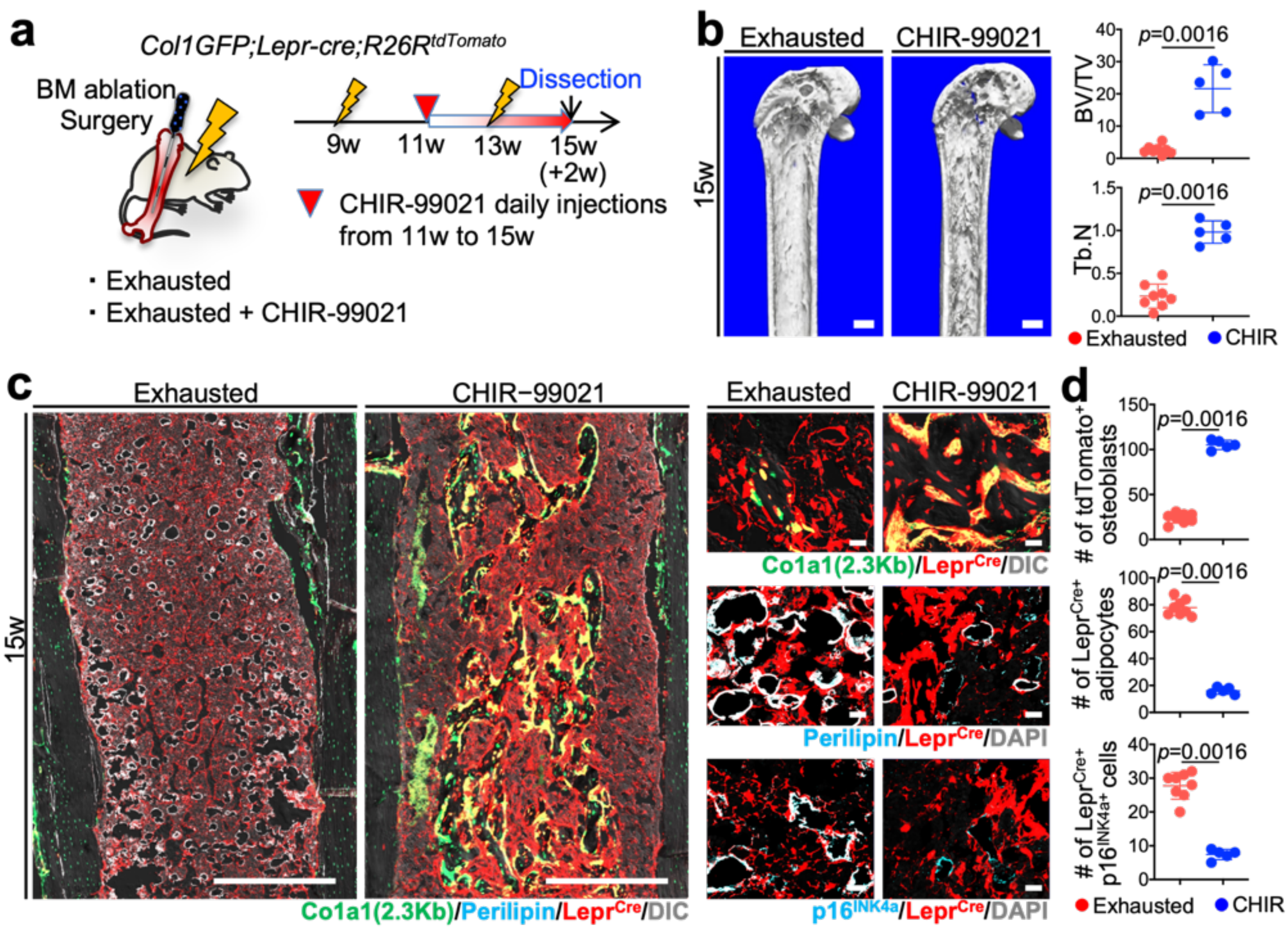

In addition, we genetically activated Wnt/β-catenin signaling using *Lepr-cre*; *Ctnnb1*$^{lox(ex3)/+}$; *R26R*^{tdTomato} mice, in which β-catenin is constitutively stabilized in Lepr-cre^{+} stromal lineage cells. Notably, stabilization of β-catenin markedly suppressed the adipogenic conversion of stromal cells after repeated injury and preserved their osteogenic differentiation potential. These data have been included as revised **Figures 10e-h**.

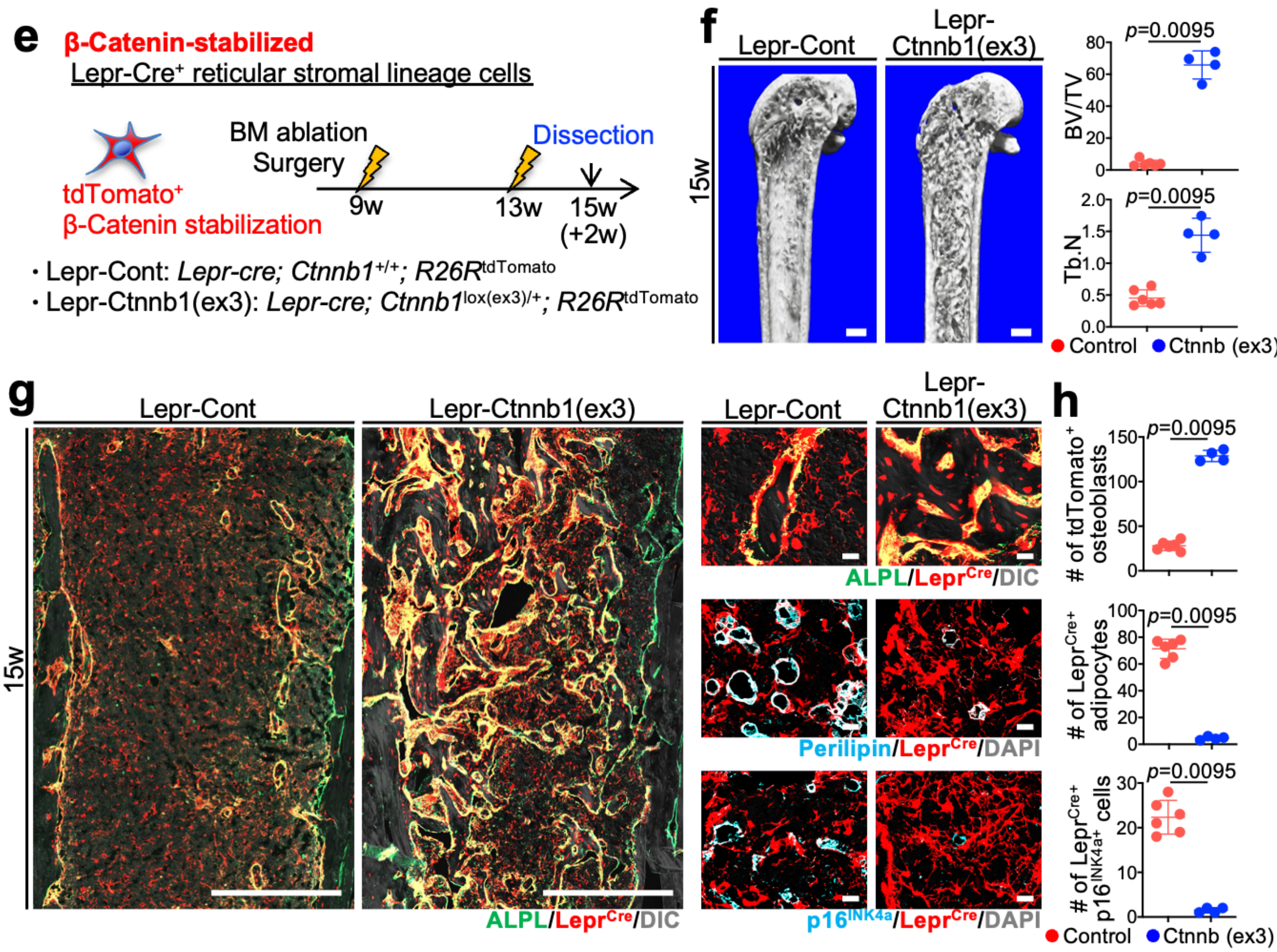

Together, these complementary pharmacological and genetic approaches demonstrate that activation of Wnt/β-catenin signaling is sufficient to rescue key aspects of the exhausted phenotype, supporting a mechanistic role for impaired β-catenin reactivation in stromal exhaustion.

The following sentences have been added to the main text:

Line 113-115

Conversely, both pharmacological and genetic activation of Wnt/β-catenin signaling restores osteogenic regeneration and suppresses adipogenic conversion, supporting its causal role in preventing stromal exhaustion.

Line 474-493

To determine whether activation of Wnt/β-catenin signaling could reverse the exhausted phenotype, we next performed pharmacological and genetic rescue experiments.

First, we administered daily injections of CHIR-99021, a GSK-3 inhibitor that activates Wnt/β-catenin signaling, from 11 to 15 weeks of age, during the period of the second bone marrow ablation surgery in the repeated-injury model (Fig. 10a). Micro-CT analysis revealed that CHIR-99021 treatment significantly increased trabecular bone regeneration after repeated injury, as shown by increased BV/TV and Tb.N compared with untreated exhausted mice (Fig. 10b). Histological analysis showed that CHIR-99021 restored the contribution of Lepr^{Cre+} stromal lineage cells to Col1a1-GFP^{+} osteoblasts while reducing the accumulation of Lepr^{Cre+} adipocytes and p16INK4a-positive cells in the bone marrow (Fig. 10c,d). These findings indicate that pharmacological activation of Wnt/β-catenin signaling partially rescues the impaired osteogenic response and suppresses adipogenic conversion in exhausted stroma.

Second, we further examined whether genetic activation of β-catenin in Lepr^{+} stromal lineage cells could rescue stromal exhaustion. For this purpose, we used Lepr-cre; Ctnnb1$^{lox(ex3)/+}$; R26R^{tdTomato} (Lepr-Ctnnb1(ex3)) mice, in which β-catenin is constitutively stabilized in Lepr^{Cre+} cells (Fig. 10e). Following repeated injury, Lepr-Ctnnb1(ex3) mice showed increased bone mass compared with exhausted controls (Fig. 10f). Histological analysis further revealed that β-catenin stabilization preserved osteogenic differentiation of Lepr^{Cre+} stromal lineage cells and markedly suppressed their adipogenic conversion, with a concomitant reduction in p16^{INK4a} expression in the exhausted marrow environment (Fig. 10g,h).

Line 498-502

Conversely, both pharmacological activation and genetic stabilization of β-catenin restored osteogenesis and suppressed adipogenic conversion after repeated injury. Together, these loss- and gain-of-function approaches identify impaired Wnt/β-catenin reactivation as a key mechanism underlying stromal exhaustion.

Line 586-592

Therapeutically, ~~our findings highlight actionable strategies to reverse stromal exhaustion, with the Wnt/β-catenin pathway emerging as a possible target~~ our rescue experiments identify Wnt/β-catenin signaling as a validated target for reversing key features of stromal exhaustion. Suppression of this pathway drives adipogenic bias and loss of osteogenic function in Lepr+ stromal cells, whereas pharmacological activation with CHIR-99021 or genetic stabilization of β-catenin restores osteogenic regeneration and suppresses adipogenic conversion after repeated injury. ~~suggesting~~ These findings suggest that restoring canonical Wnt signaling may ~~rejuvenate the niche~~ re-establish regenerative stromal function within the exhausted marrow niche. Potential translational approaches include sclerostin-neutralizing antibodies (e.g., romosozumab), already approved for osteoporosis, pharmacological activation via GSK3β inhibitors, or development of Wnt ligand mimetics.

Line 602-604

Importantly, the reversibility of key exhausted features through Wnt/β-catenin activation suggests that stromal exhaustion is a therapeutically targetable form of regenerative failure.

3. <Reviewer>

Given that WNT/β-catenin signaling is essential for osteogenesis (Day et al., Dev Cell 2005, Hill et al., Dev Cell 2005, Chen et al., JBMR 2013), and LepR⁺ cells serve as a source of osteoblasts, it is somewhat surprising to this reviewer that Lepr-Ctnnb1 cKO mice exhibit normal bone volume under steady-state conditions (Fig.7b). The authors should explain why the deletion of β-catenin in LepR⁺ cells did not affect bone metabolism.

<Response>

Thank you for this important comment. We agree that Wnt/β-catenin signaling is well known to play a critical role in osteogenesis. The reviewer raises an important question as to why deletion of β-catenin in Lepr⁺ stromal cells did not lead to a reduction in bone mass under steady-state conditions in young-adult mice. One important explanation relates to the timing of Lepr⁺ lineage contribution to osteoblasts. Previous studies have shown that Lepr-cre⁺ stromal lineage cells contribute relatively little to osteoblasts and osteocytes in young-adult bone, and their contribution gradually increases with age (Zhou et al., Cell Stem Cell 2014). Consistent with this model, although we did not observe a clear bone phenotype in young mice, in the revised study, we additionally analyzed *Lepr-cre*; *Ctnnb1*$^{fl/fl}$ mice at 6 months of age and observed a reduction in bone mass. This result suggests that Wnt/β-catenin signaling in Lepr-cre⁺ stromal lineage cells becomes increasingly important for bone homeostasis as their contribution to the osteoblast lineage increases with age. These data have been included as revised **Figure 9b**.

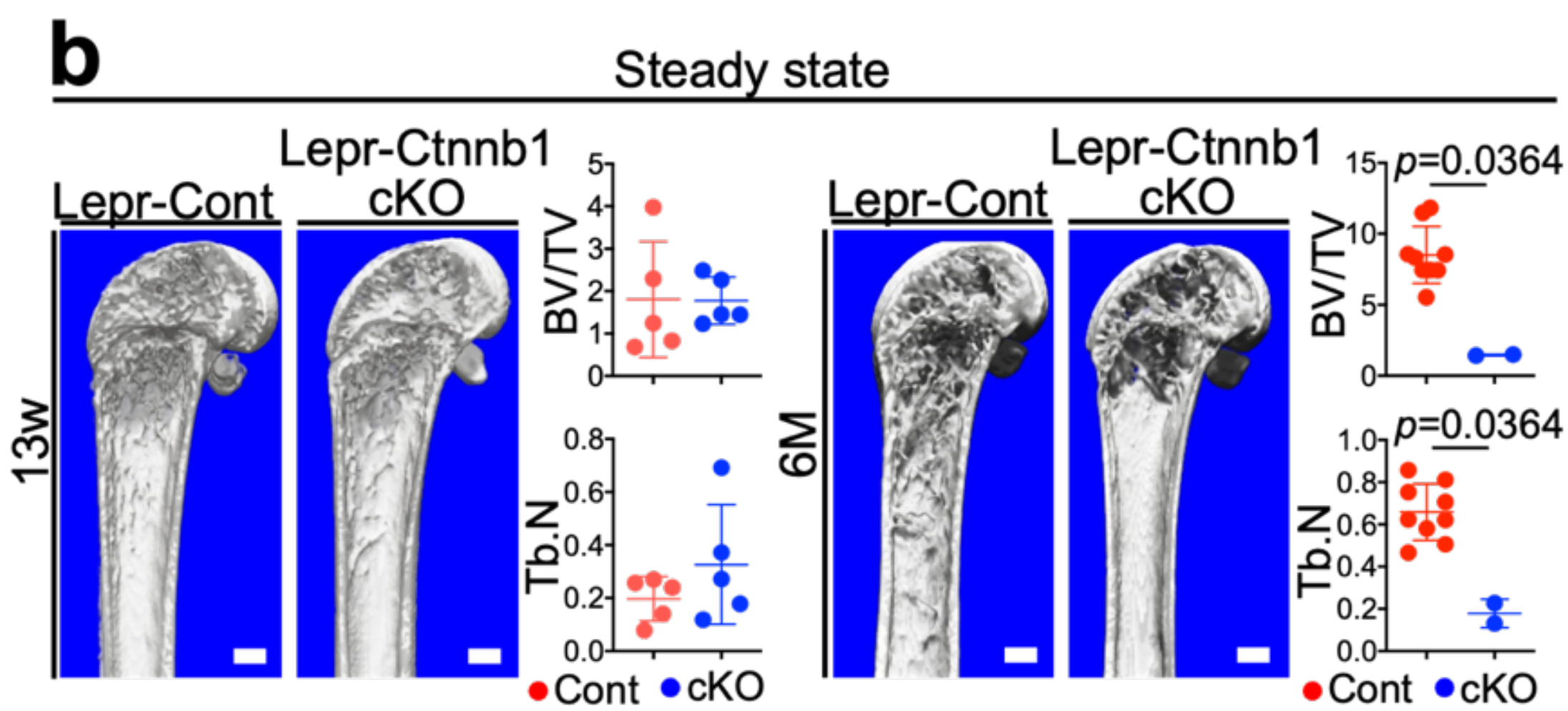

In addition, our previous work demonstrated that Cxcl12⁺ reticular stromal cells can dedifferentiate into skeletal stem cell–like cells during bone regeneration (Matsushita et al., Nat Commun 2020), in which Wnt/β-catenin signaling becomes activated and contributes to osteogenic regeneration. This suggests that Wnt signaling in Lepr⁺Cxcl12⁺ stromal cells may play a particularly important role during injury-induced regenerative activation, rather than during steady-state bone maintenance. Furthermore, our other study showed that Fgfr3⁺ endosteal skeletal stem cells and their downstream Gas1⁺ osteo-stromal progenitors give rise to Lepr⁺Cxcl12⁺ reticular stromal cells and osteoblasts during bone growth (Matsushita et al., Nat

Commun 2023). Consistent with this hierarchical model, we additionally performed *Ctnnb1* deletion in upstream skeletal stem and progenitor populations using *Fgfr3-creER* and *Gas1-creER* mouse lines. This resulted in significant bone loss, indicating that Wnt/β-catenin signaling plays an essential role in these earlier skeletal lineages in young adults. Although these data are not central to the main conclusions of the revised manuscript, they are provided **below** to address the reviewer's question.

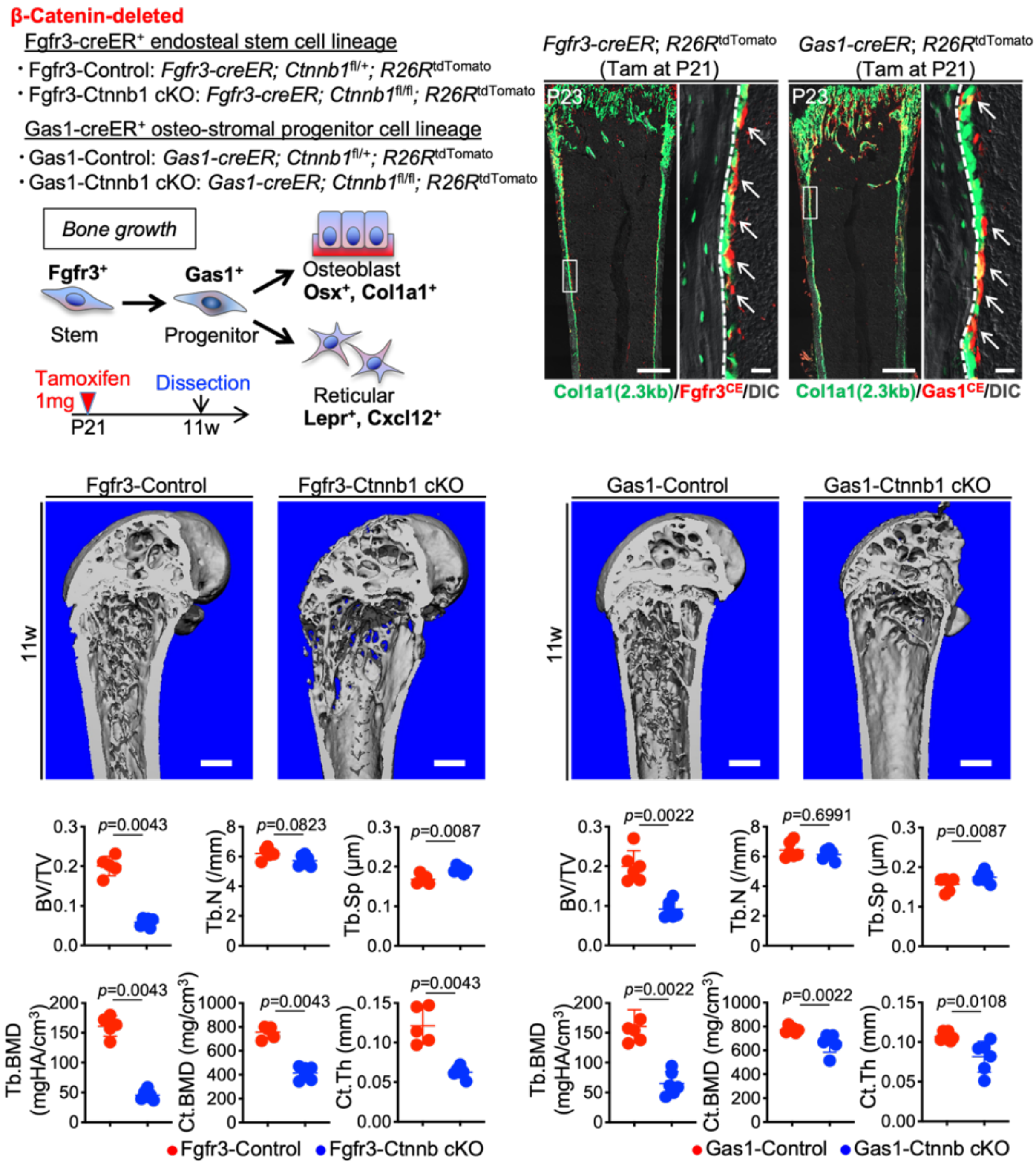

Taken together, these findings suggest that Wnt/β-catenin signaling functions in a stage- and lineage-dependent manner, playing a critical role in upstream skeletal stem/progenitor cells during steady-state bone homeostasis, while becoming activated in Lepr+ reticular stromal cell lineages primarily during injury-induced regeneration.

The following sentences have been added to the main text:

Line 454-459:

Micro-CT analysis of femurs revealed no significant differences in trabecular BV/TV or Tb.N between control and Lepr-Ctnnb1 cKO mice under uninjured conditions <u>*at 13 weeks of age (Fig. 9b). In contrast, Lepr-Ctnnb1 cKO mice showed reduced trabecular bone mass at 6 months of age (Fig. 9b), suggesting that β-catenin signaling in Lepr$^+$ stromal lineage cells becomes increasingly important for bone homeostasis as their contribution to the osteoblast lineage increases with age.*</u>

4. <Reviewer>

It is interesting that inflammatory pathways, including IL-17 signaling and the Toll-like receptor cascade, are enriched in exhausted stromal cells. The authors should discuss the possibility that the bone marrow immune system is involved in injury-induced stromal exhaustion.

<Response>

Thank you for this insightful suggestion. We agree that the enrichment of inflammatory pathways in exhausted stromal cells raises the possibility that the bone marrow immune microenvironment contributes to injury-induced stromal exhaustion. In response to the reviewer's comment, we have added a paragraph to the Discussion addressing this possibility. In the revised manuscript, we discuss that repeated injury may create a persistently inflammatory niche, including activation of IL-17 signaling and Toll-like receptor pathways, which could suppress regenerative stromal programs and contribute to the exhausted stromal state.

The following sentences have been added to the main text:

Line 526-531:

The enrichment of IL-17 signaling and Toll-like receptor pathways in exhausted stroma further suggests that the bone marrow immune microenvironment may contribute to stromal exhaustion. Repeated injury may generate a sustained inflammatory microenvironment that interferes with regenerative stromal responses, including Wnt/β-catenin–associated osteogenic reactivation, and instead promotes maladaptive remodeling.

Response to Reviewer #2

Thank you very much for your constructive comments and critiques.

0. <Reviewer>

In this manuscript Wu et al. examine the outcomes and cellular basis of the response to repeated injury in the skeletal system, ultimately establishing that impaired healing after repeated injury correlates with the presence of an "exhausted" state in a specific populations of Cxcl12 abundant reticular cells (CAR cells). Overall, there is a high degree of enthusiasm for this study, as concepts of progenitor exhaustion after repeated insults and trained immunity have emerged as important areas of study in other organ systems yet have largely not been similarly establishing in the bone field. Thus, this manuscript is felt to represent an important conceptual advance. Overall, the study is felt to be sound in approach, with most of the comments below representing requests for clarification or to adjust the terminology used. The only major comments are that this study would likely benefit from a bit more data to fully flesh out the relationship of this exhaustion state to sensenece.

<Response>

Thank you very much for your critical assessment on our study.

Major points

1. <Reviewer>

The senescence field in bone now typically holds that a single marker is insufficient to confidently determine that cells are senescent. A combination of numerous markers is now required as in work by Khosla and colleagues. If the authors want to make senescence claims regarding the exhausted CAR cells, a similar combination (p21 in addition to the p16 here, SA beta gal, evidence of senescence associated secretory phenotypes (SASP) ect...). However, this reviewer wonders if it is correct or desirable to effectively equate the exhaustion phenotype with the senescence phenotype that has now become widely studied in bone. It appears likely from both first principles and perhaps also the data here showing that the exhaustion state is not equivalent to aging that exhaustion is a distinct phenomenon from senescence. In this respect, demonstrating the differences between exhaustion and senescence is perhaps more important for the impact of the manuscript than emphasizing the similarities. However, this is just speculation on the part of this reviewer, and the data may ultimately indicate that exhaustion is highly similar to senescence. Ultimately, the relationship of the exhaustion phenotype to senescence should be better fleshed out with additional data.

<Response>

We thank the reviewer for this insightful and constructive comment. The reviewer's suggestion prompted us to more carefully examine the relationship between stromal exhaustion and cellular senescence, which significantly strengthened the conceptual framework of our study.

First, to evaluate cellular senescence more rigorously, we performed additional experiments using SA-β-gal staining and *p16-creER*; $R26R^{tdTomato}$ reporter mice to assess senescence after bone marrow

injury. These analyses revealed that senescent cells indeed increased in the bone marrow after injury. However, interestingly, senescence markers were predominantly detected in adipocytes, whereas stromal cells themselves showed relatively limited senescence signals. This observation suggests that the exhaustion phenotype of stromal cells is not simply a consequence of classical cellular senescence. These data have been included as revised **Figures 2d-h**.

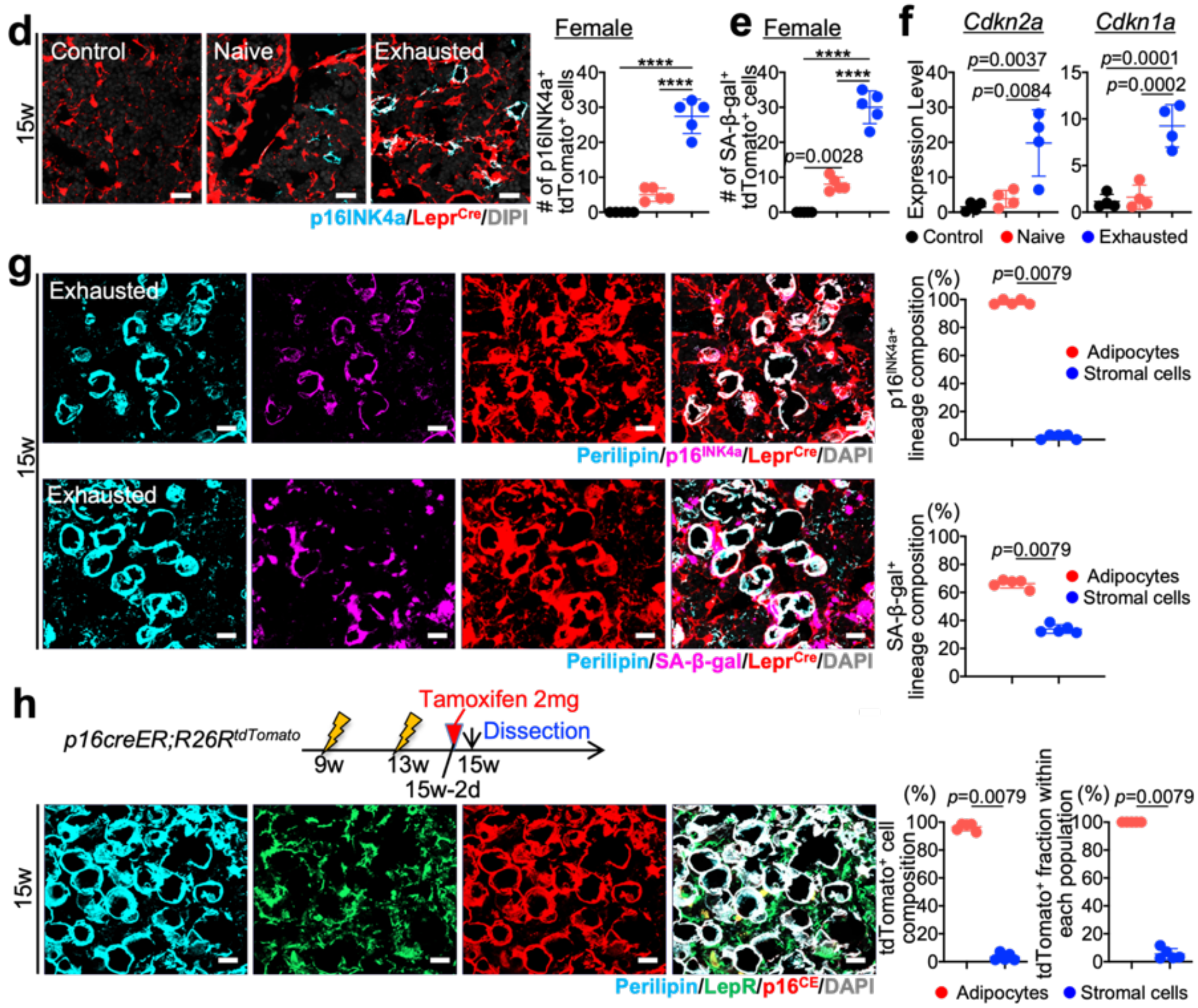

Additional related data are shown in the revised **Extended data figure 5**.

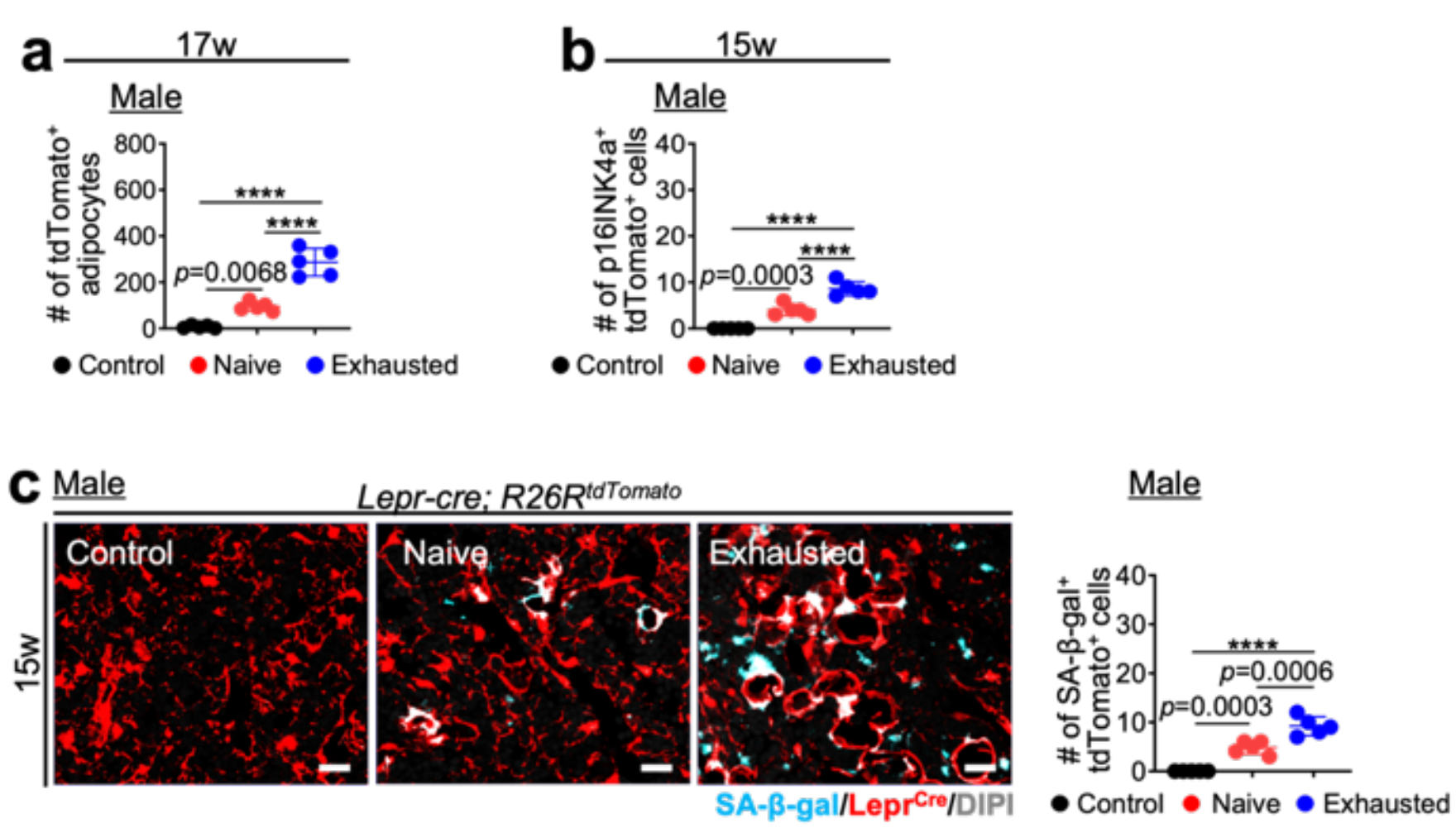

To further clarify the distinction between exhaustion and aging, we next performed bone marrow ablation in aged (18-month-old) mice, followed by histological analysis and scRNA-seq profiling of stromal populations. Although regenerative capacity in aged mice was reduced relative to young mice and partial adipogenic differentiation was observed, new bone formation still occurred after injury, and Lepr-cre^{+} stromal cells retained osteogenic differentiation potential, clearly contrasting with the exhausted condition. These data have been included as revised **Figure 6**.

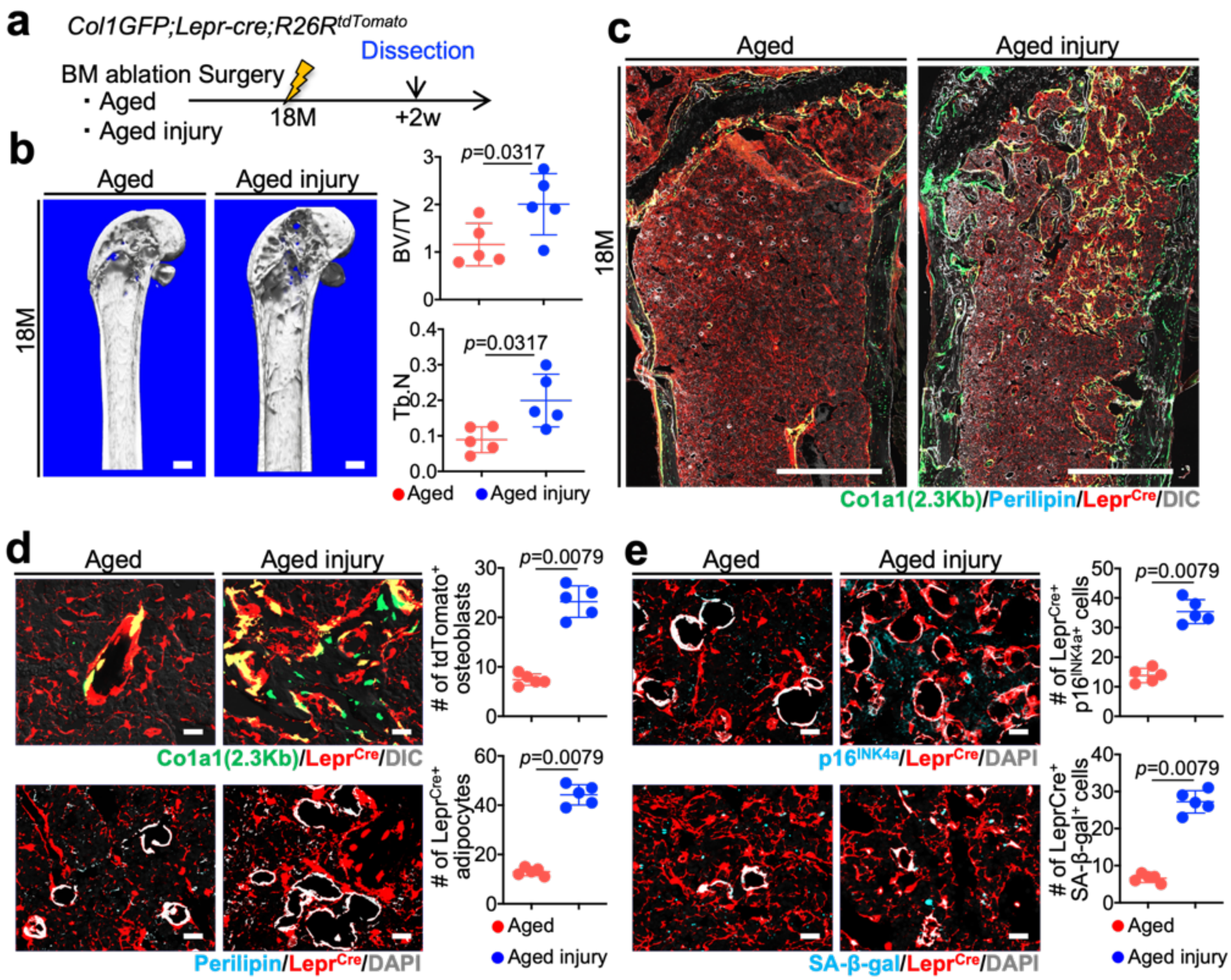

At the molecular level, pathway enrichment analysis revealed that aged-injury stroma was characterized by activation of regenerative programs, including Wnt signaling, TGF-β signaling, chondrocyte differentiation, connective tissue development, and vasculature development. In contrast, exhausted stroma showed enrichment of inflammatory and stress-associated pathways, including TNF signaling, NF-κB/STAT3-related responses, and extracellular matrix remodeling. These results indicate that aged stromal cells maintain coordinated injury-responsive regenerative programs, whereas exhausted stroma fails to properly reactivate these pathways after repeated injury. These data have been included as revised **Extended Data figures 8a,b**.

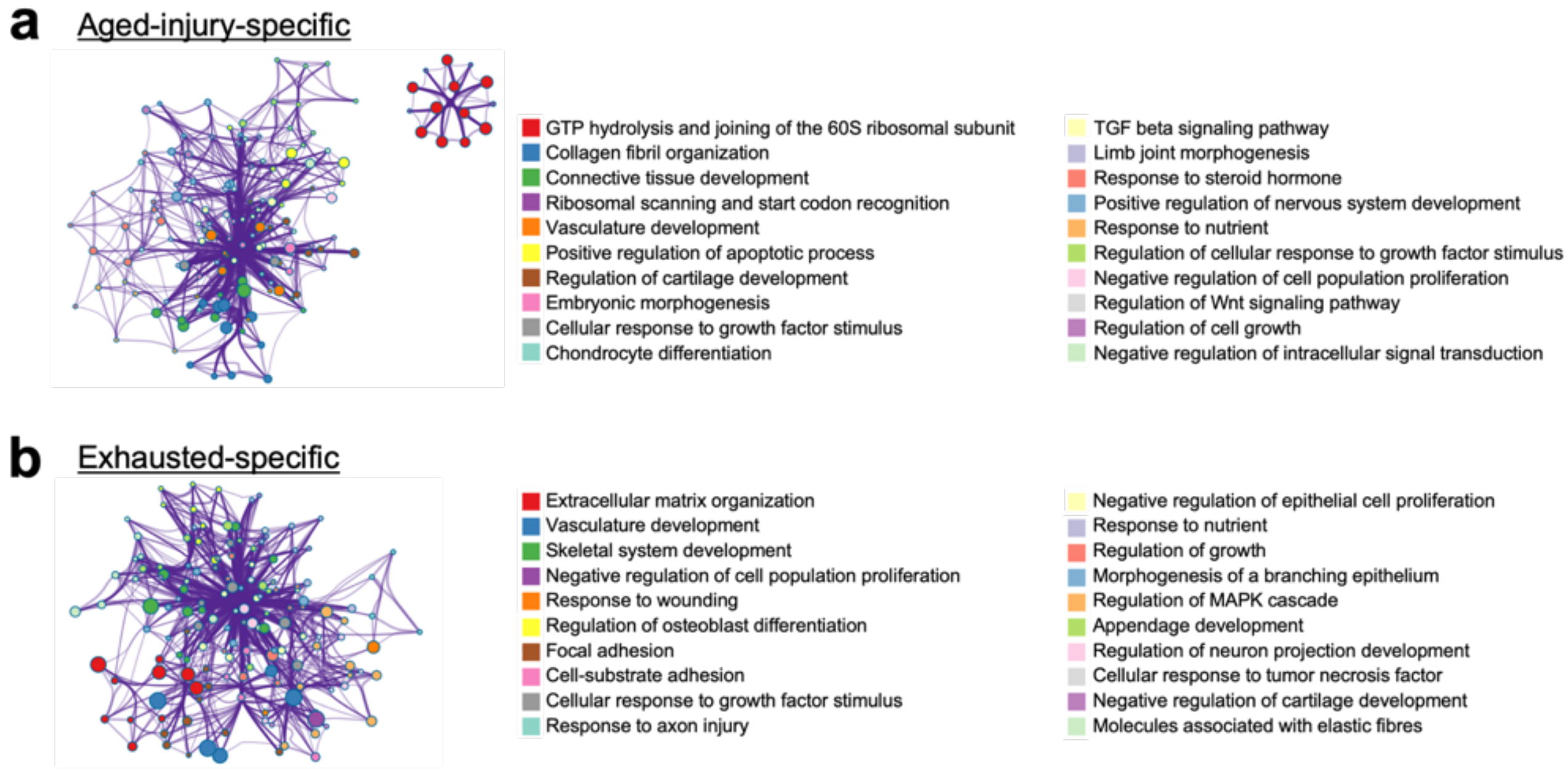

Taken together, these findings indicate that stromal exhaustion represents a state distinct from both physiological aging and classical cellular senescence, characterized primarily by the loss of injury-induced regenerative competence rather than the accumulation of senescent stromal cells. We have revised the manuscript to clarify this conceptual distinction.

The following sentences have been added to the main text:

Line 107-109:

~~While cellular senescence constitutes an aspect of this state~~ In this context, stromal exhaustion denotes a durable, stress-induced decline in plasticity and regenerative function that is mechanistically and phenotypically distinct from physiological aging.

Line 194:

3. Injury-driven exhausted stromal cells promote adipogenic differentiation ~~and exhibit cellular senescence~~

Line 213-215:

we performed immunofluorescence staining for the senescence-associated marker p16^{INK4a} and SA-β-gal staining. Two weeks post-surgery, LeprCre-tdTomato+p16^{INK4a+} and LeprCre-tdTomato+ SA-β-gal^{+} senescent cells were increased in the exhausted group

Line 219-233:

However, further histological analysis revealed that p16INK4a and SA-β-gal signals were predominantly detected in perilipin^{+} adipocytes rather than in LeprCre-tdTomato^{+} stromal cells (Fig. 2g). Consistently, p16-creER; R26R^{tdTomato} lineage cells labeled after the second injury showed that p16+ cells largely overlapped with adipocytes, whereas only a limited fraction overlapped with LepR^{+} stromal cells (Fig. 2h). These findings suggest that, although senescence-associated cells accumulate in the exhausted marrow

environment, the exhausted phenotype of stromal cells is not simply explained by classical cellular senescence of the stromal population itself.

In summary, our findings demonstrate that exhausted bone marrow stromal cells undergo significant adipogenic differentiation ~~and increased senescence post-injury~~, leading to elevated marrow fat accumulation. ~~Moreover, cellular senescence may underlie injury-driven stromal exhaustion and potentially contribute to impaired bone regeneration and marrow homeostasis.~~ Although senescence-associated cells increased in the exhausted marrow environment, these signals were predominantly associated with adipocytes rather than stromal cells themselves. Thus, the exhausted phenotype is characterized primarily by adipogenic conversion and loss of stromal regenerative capacity, rather than being simply explained by classical cellular senescence of stromal cells.

Line 328-344:

7. Stromal exhaustion is distinct from physiological aging

To determine whether stromal exhaustion simply reflects physiological aging of bone marrow stromal cells, we examined the regenerative response of aged stromal cells after injury. We performed bone marrow ablation surgery in 18-month-old Lepr-cre; R26RtdTomato mice and compared their response with that of no-surgery controls (Fig. 6a). Micro-CT analysis revealed that significant trabecular bone formation still occurred after injury (Fig. 6b). Histological analysis further showed that Lepr-cre lineage cells in aged mice retained the ability to contribute to osteoblasts after injury, whereas adipocyte accumulation was limited in aged mice under steady-state conditions and increased only modestly after injury (Fig. 6c,d). We also examined senescence-associated changes and found that the aged group showed only limited senescence-associated signals under steady-state conditions, although these signals increased after injury (Fig. 6e). Together, these results suggest that aged stromal cells retain injury-induced regenerative capacity, despite partial reductions in osteogenesis and increases in adipogenesis or senescence-associated features. Thus, stromal exhaustion is distinct from physiological aging and represents an injury-induced loss of regenerative capacity rather than a simple age-associated decline.

Line 392-401:

To further distinguish stromal exhaustion from physiological aging, we compared exhausted stroma with aged-injury stroma (Extended Data Fig. 8a,b). Pathway enrichment analysis revealed that aged-injury stroma retained injury-responsive regenerative programs, including Wnt signaling, TGF-β signaling, connective tissue development, and vasculature development. In contrast, exhausted stroma showed enrichment of inflammatory and stress-associated pathways, including TNF signaling, NF-κB/STAT3-related responses, and extracellular matrix remodeling. These findings indicate that aged stromal cells maintain coordinated injury-responsive regenerative programs, whereas exhausted stroma fails to properly reactivate these pathways after repeated injury, further supporting stromal exhaustion as a distinct injury-induced state rather than a simple extension of physiological aging.

Line 511-513:

They exhibit features of exhaustion, including impaired clonogenicity, diminished osteogenic capacity, and increased adipogenic ~~and senescent~~ characteristics,

Line 519:

Loss of Wnt/β-catenin activity not only promotes adipogenic shift ~~but also contributes to cellular senescence, forming a self-reinforcing cycle of dysfunction. Wnt/β-catenin signaling serves as a conserved guardian against cellular senescence, as its suppression accelerates senescence in diverse tissues, including the brain, kidney, and stem cell compartments. For example, β-catenin deficiency in mouse embryonic stem cells causes telomere shortening and premature aging, while pharmacological or genetic restoration of Wnt signaling in aged models of Parkinson's disease reactivates neurogenesis and promotes neural repair~~.

Line 535:

with stromal cells exhibiting features of adipogenic commitment~~ and cellular senescence~~.

2. <Reviewer>

In Fig 7, 13 weeks may be a bit young to see a phenotype at steady state from gene deletion in CAR cells, as past literature generally shows that CAR cell phenotypes have an onset after 4-5 months of age due to CAR cells emerging postnatally and having a gradually increasing contribution to the osteoblast pool, with excellent examples of these dynamics being described in the authors' past work. Ideally, this would be clarified with additional later timepoints, but it would also be acceptable to just robustly qualify the negative baseline data in the text without additional experimental data

<Response>

Thank you very much for your important comments. As the reviewer suggested, CAR cell-related phenotypes generally emerge after 4–5 months of age or later (Zhou et al., Cell Stem Cell 2014; Seike et al., Genes Dev 2018). To address the role of *Ctnnb1*-deleted CAR cells in detail, we assessed the phenotype of *Lepr-cre; Ctnnb1*$^{fl/fl}$*; R26R*^{tdTomato} (Lepr-Ctnnb1 cKO) and *Lepr-cre; Ctnnb1*$^{fl/+}$*; R26R*^{tdTomato} (Control) at 6 months of age. Importantly, Lepr-Ctnnb1 cKO mice exhibited reduced bone mass at 6 months of age, supporting the idea that the skeletal phenotype emerges at a later stage. These data have been included as revised **Figure 9b**.

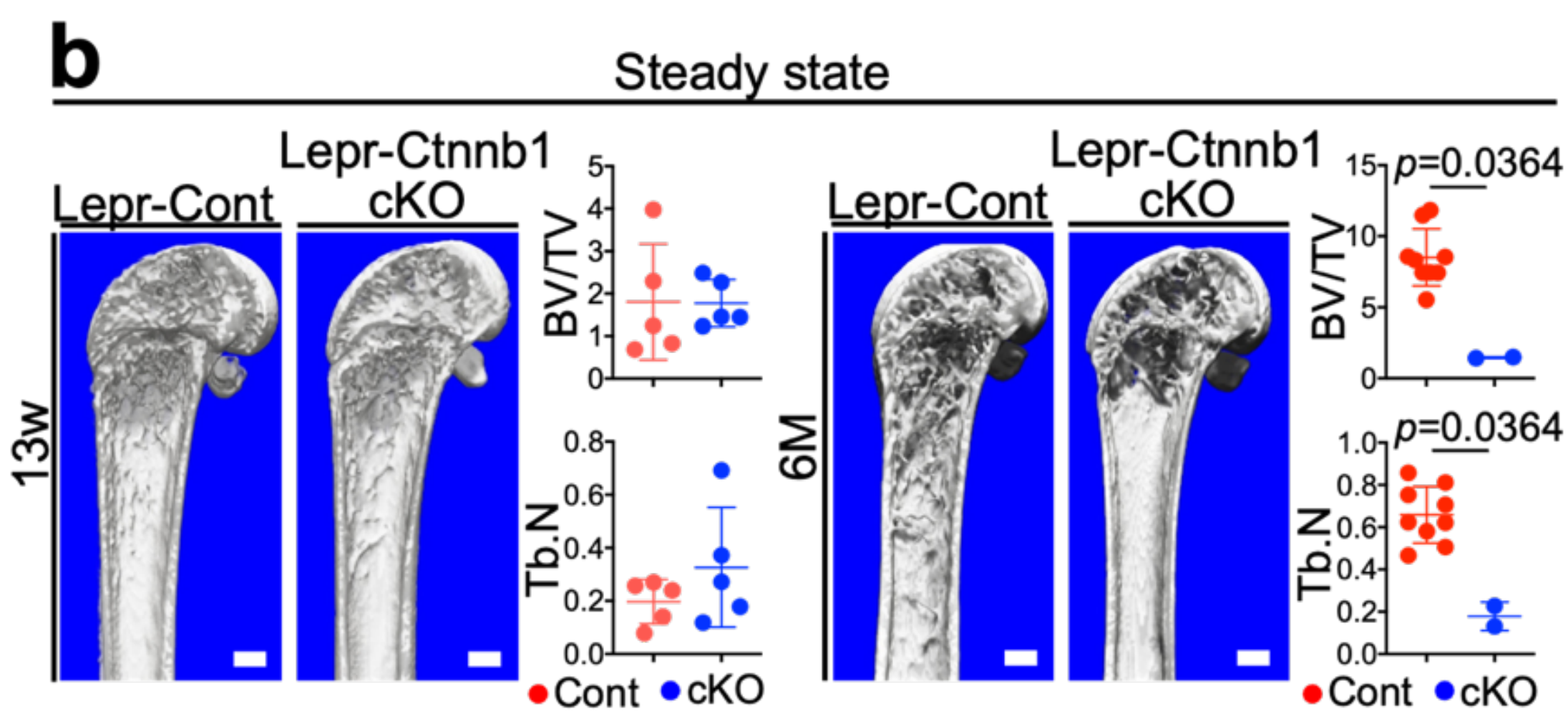

In addition, we investigated which mesenchymal cell sources are affected by Wnt/β-catenin signaling in the young-adult stage. Our previous work (Matsushita et al., Nat Commun 2023) demonstrated that Fgfr3^{+} endosteal stem cells and subsequent downstream Gas1^{+} osteo-stromal progenitor cells in bone marrow differentiate into Lepr^{+}Cxcl12^{+} reticular stromal cells and osteoblasts during bone growth. Therefore, we predicted that *Ctnnb1* deletion affects the upstream skeletal cells of Lepr^{+}Cxcl12^{+} reticular stromal cells. Then, we additionally investigated the role of *Ctnnb1* in Fgfr3^{+} endosteal stem cell lineage and Gas1^{+} osteo-stromal progenitor cell lineage in bone development. We took advantage of *Fgfr3-creER; Ctnnb1*$^{fl/fl}$*; R26R*^{tdTomato} (Fgfr3-Ctnnb1 cKO), *Fgfr3-creER; Ctnnb1*$^{fl/+}$*; R26R*^{tdTomato} (Fgfr3-Control), *Gas1-creER; Ctnnb1*$^{fl/fl}$*; R26R*^{tdTomato} (Gas1-Ctnnb1 cKO), and *Gas1-creER; Ctnnb1*$^{fl/+}$*; R26R*^{tdTomato} (Gas1-Control) mice. We injected tamoxifen at P21 and harvested the femurs at 11 weeks. Both Fgfr3-Ctnnb1 cKO and Gas1-Ctnnb1 cKO showed a significant bone loss in trabecular and cortical bones (more severe loss in Fgfr3-Ctnnb1 cKO). Although these data are not central to the main conclusions of the revised manuscript, they are provided **below** to address the reviewer's question.

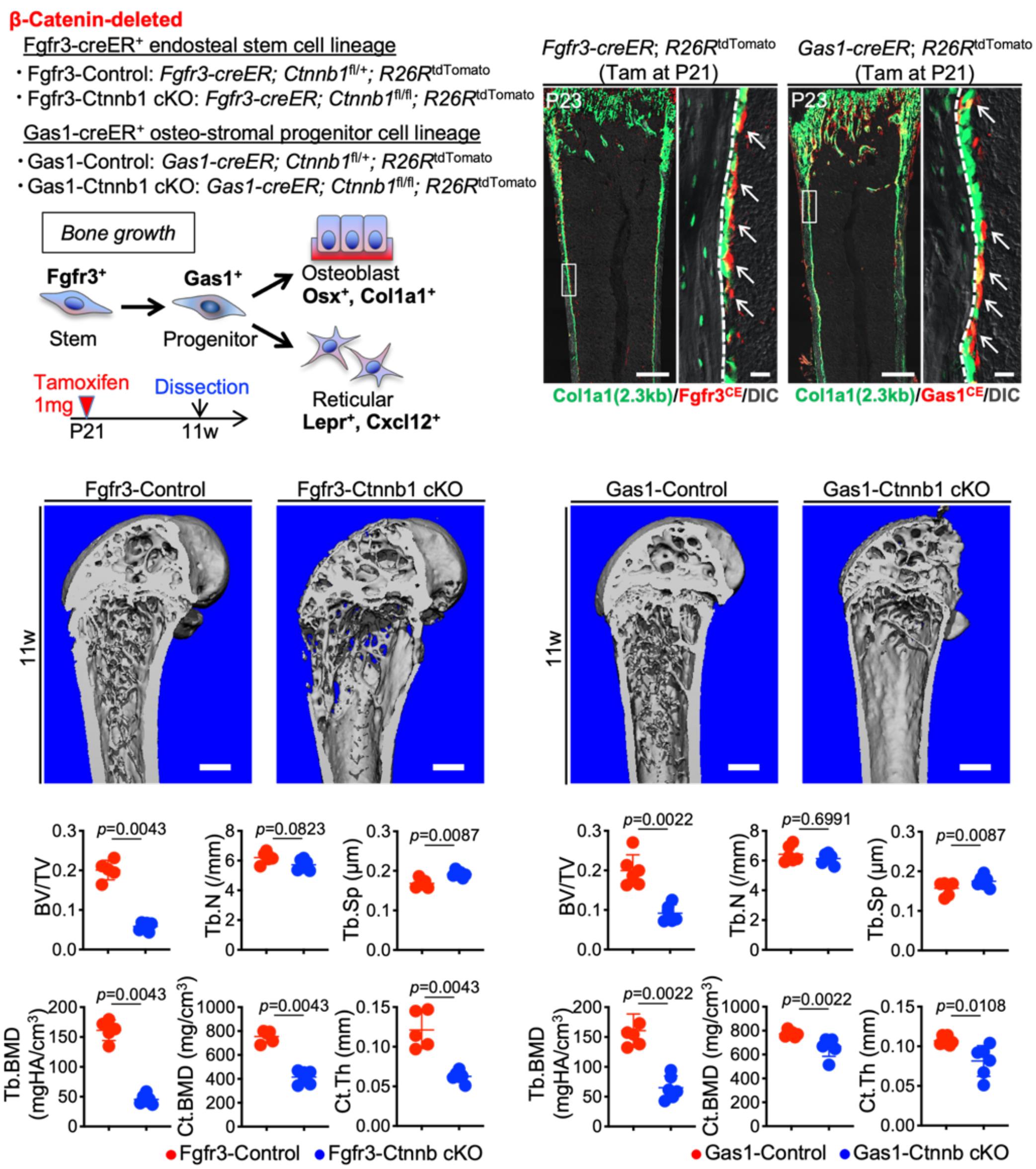

Together, these additional analyses support our conclusion that Wnt/β-catenin signaling is required for mesenchymal skeletal cell function in an age- and lineage-dependent manner, and further strengthen the interpretation of the Lepr-Ctnnb1 cKO phenotype.

The following sentences have been added to the main text:

Line 454-459:

Micro-CT analysis of femurs revealed no significant differences in trabecular BV/TV or Tb.N between control and Lepr-Ctnnb1 cKO mice under uninjured conditions *at 13 weeks of age (Fig. 9b). In contrast, Lepr-Ctnnb1 cKO mice showed reduced trabecular bone mass at 6 months of age (Fig. 9b), suggesting that β-catenin signaling in Lepr^{+} stromal lineage cells becomes increasingly important for bone homeostasis as their contribution to the osteoblast lineage increases with age.*

Minor points

3. <Reviewer>

The authors' use of the term "stem cell" to refer to CXCL12 abundant reticular (CAR) cells is unexpected and perhaps not the most accurate choice of terminology, especially as it is the authors' own work (especially Matsushita et al. Nat Comms 2023, Fig 4, but also Mizuhashi et al. Nature 2018 and even the Osx-based lineage tracing data here and also in Ono et al. Nat Cell Bio 2014) that provides clear and direct evidence of populations that are upstream of the postnatal generation of CAR cells. Given that perhaps the most direct definition of a skeletal stem cell is a self-renewing population that sits at the apex of their differentiation hierarchy, this would preclude the use of stem cell terminology to refer to CAR cells. From this perspective, it is recommended to avoid stem cell terminology in this manuscript to avoid further confusion on this point in the literature. Simialrly, "loss of stemness" should be removed from the text of Fig 8, as none of the assays performed here are appropriate for assessing stemness, as serial transplantation or similar assays would be required.

<Response>

We thank the reviewer for this important and constructive comment. We recognize that the terminology surrounding adult bone marrow stromal populations has varied in the literature. Adult Lepr^{+}Cxcl12^{+} cells are maintained long-term *in vivo* and can contribute to osteogenic and adipogenic lineages, and in some contexts to chondrocytes after injury. In addition, single-cell–derived clones can undergo serial passaging and tri-lineage differentiation *ex vivo* (Matsushita et al., Nat Commun 2020). However, we agree with the reviewer that these properties do not establish CAR cells as bona fide skeletal stem cells under a strict definition.

We also agree that, within the postnatal skeletal hierarchy, upstream populations that give rise to adult CAR cells have been identified by several lineage-tracing studies, including Fgfr3^{+} endosteal cells, Pthrp^{+} growth plate resting cells, and Osx^{+} cells (Matsushita et al., Nat Commun 2023; Mizuhashi et al., Nature 2018; Mizoguchi et al., Dev Cell 2014).

Therefore, to avoid ambiguity and to align with the reviewer's suggestion, we have removed "stem cell" and "stemness" terminology when referring to CAR cells throughout the manuscript. In Fig. 8, we removed the term "*stemness*" and revised "*Loss of stemness/functional plasticity*" to "*Loss of functional plasticity*," which more accurately reflects the scope of our assays. We believe these changes improve terminological clarity and avoid overinterpretation. We are grateful for the reviewer's constructive guidance.

The following sentences have been added to the main text:

Line 38-39:

Bone marrow stromal cells, marked by leptin receptor (Lepr) and C-X-C motif chemokine ligand 12 (Cxcl12), ~~serve as adult skeletal stem cells that~~ orchestrate osteogenesis and maintain bone homeostasis.

Line 43-44:

However, it remains unclear whether these stromal cells retain their full regenerative capacity ~~as stem cells~~ following prior injury.

Line 48-49:

These exhausted stromal cells are clearly distinct from aging stromal cells, as they ~~lack stemness,~~ exhibit metabolic abnormalities

Line 64-65:

In the process of bone injury repair, skeletal stem and progenitor cells ~~(SSCs)~~ residing in skeletal tissues play a crucial role.

Line 77-78:

pointing to an intrinsic limitation in ~~stem~~ regenerative cell function over time.

Line 80-81:

they may enter a state of functional exhaustion, characterized by ~~loss of stemness~~ impaired osteogenic potential and diminished regenerative capacity.

Line 84-87:

leptin receptor (Lepr)- and C-X-C motif chemokine ligand 12 (Cxcl12)-expressing reticular stromal cells in the central marrow space contribute to regeneration, particularly in adulthood, through injury-induced osteogenic differentiation and cellular plasticity.

Line 102-103:

whether Lepr+Cxcl12+ stromal cells can indefinitely retain their ~~stem cell~~ regenerative potential after injury remain unclear.

Line 599-601:

This dysfunctional state, characterized by impaired osteogenesis and adipogenic lineage shift, ~~and increased cellular senescence,~~ reflects a loss of ~~stemness and~~ functional plasticity in stromal cells.

4. <Reviewer>

Related to the point above, the choice in the introduction to present FGFR3+ endosteal cells and CAR cells as parallel populations is similarly unexpected, given that the authors' prior work demonstrate that FGFR3

cells are upstream of CAR cell production. This would appear to indicate that describing these two populations in a hierarchical manner is more appropriate than describing them as parallel.

<Response>

Thank you very much for this suggestion. We have revised the Introduction to avoid a parallel description of Fgfr3+ cells and CAR cells, and instead clarified the hierarchical relationship. Specifically, we now describe CAR cells as a downstream stromal population that is provided by upstream progenitors during bone growth, including cells associated with the fetal cartilage anlagen/perichondrium, growth plate SSCs, mesenchymal metaphyseal progenitors, and $Fgfr3^{+}$ endosteal SSCs. We believe this revision better reflects the differentiation hierarchy and improves conceptual clarity.

The following sentences have been added to the main text:

Line 84-86:

Within the bone marrow environment, leptin receptor (Lepr)- and C-X-C motif chemokine ligand 12 (Cxcl12)-expressing reticular stromal cells in the central marrow space contribute to regeneration

Line 88-92:

…these stromal cells are provided by distinct developmental skeletal stem and progenitor cells, including fetal chondrocytes and perichondrial cells, early postnatal growth plate stem cells, mesenchymal metaphyseal progenitors, and fibroblast growth factor receptor 3 (Fgfr3)-expressing juvenile endosteal stem cells.

5. <Reviewer>

Figure 1 feels somewhat disconnected from the rest of the manuscript, and it feels like the manuscript properly starts with Fig 2 as the main topic of exhaustion is addressed. Conversely, perhaps the data in Ext Fig 3 is very important to the main points of the manuscript and could be considered for placement in the main text figures. However, this is just an optional suggestion for consideration.

<Response>

Thank you for the helpful suggestion. We have moved the original Figure 1 to the Extended Data and promoted the original Extended Data Figure 3 to the main figure in the revised manuscript. We believe this revision improves the overall organization and better aligns the figures with the manuscript's central theme.

The following sentences have been added to the main text:

Line 267-268:

5. Inducible Cxcl12-creER lineage tracing validates stromal exhaustion in pre-existing reticular stromal cells

Line 270-271:

To further ~~confirm these findings~~ validate stromal exhaustion using a temporally controlled lineage-tracing approach,

Line 272-275:

In contrast to constitutive Lepr-cre labeling, Cxcl12-creER enables pulse labeling of Cxcl12$^+$ stromal cells present at the time of tamoxifen administration, allowing their fate to be followed through subsequent injury and repair.

Line 277-286:

Cxcl12-creER–labeled (Cxcl12CE) cells showed robust osteogenic contribution in the naive group, whereas this response was markedly reduced in the exhausted group at both 2 and 4 weeks post-surgery (Fig. 4b–d). Importantly, Cxcl12CE cells persisted within the bone marrow after both single and repeated injury, indicating that pre-existing Cxcl12$^+$ stromal cells were not eliminated by injury. Conversely, the contribution of Cxcl12CE cells to marrow adipocytes was significantly increased after injury, particularly in the exhausted group (Fig. 4e–g). These findings indicate that pre-existing Cxcl12$^+$ reticular stromal cells survive repeated injury but undergo a functional shift from osteogenic toward adipogenic differentiation, further validating stromal exhaustion in a temporally defined CAR cell subset.

6. <Reviewer>

The point made in Fig 1e that CAR cells are bipotent for the production of osteoblasts and adipocytes is likely controversial given competing models that propose that distinct sets of CAR cells are responsible for adipogenesis versus osteogenesis. Perhaps it is better to not endorse the bipotency model given that this is not an issue directly addressed by the present manuscript. This point is also made in lines 203 and 204--the studies cited in that line to support the bipotency claim are excellent studies but do not substantively address whether it is a single cell type, especially a single clonal lineage of CAR cells, that produces both osteoblasts and adipocytes. This text should also be revised and cartoon model figures should be considered from the perspective that it is unclear if CAR cells represent a single population that is clonally bipotent to generate both osteoblasts and adipocytes as opposed to being a pool of multiple lineage committed progenitors with distinct subsets that predominantly mediate osteogenesis versus adipogenesis.

<Response>

We thank the reviewer for this important and thoughtful comment. We agree that the bipotency of CAR cells remains an open and controversial issue in the field, particularly because existing studies do not definitively demonstrate that a single CAR cell or a single clonal CAR-cell lineage generates both osteoblasts and adipocytes in vivo. Since this question is not directly addressed in the present manuscript, we agree that it is more appropriate to avoid endorsing the bipotency model.

Accordingly, we have removed Fig. 1e from the revised manuscript. We also revised the relevant text to avoid suggesting that CAR cells represent a single clonally bipotent population. In addition, we modified the cartoon model revised **figure 11** so that it no longer depicts the fate transition as if driven by a single individual cell. Instead, the revised model presents naive stromal cells as a population, from which some cells become osteoblast precursors and then progress to exhausted stromal cells, whereas other naive stromal cells may transition more directly into exhausted stromal cells. We believe this revised

presentation more accurately reflects the current uncertainty in the field and avoids overinterpretation beyond the scope of our data.

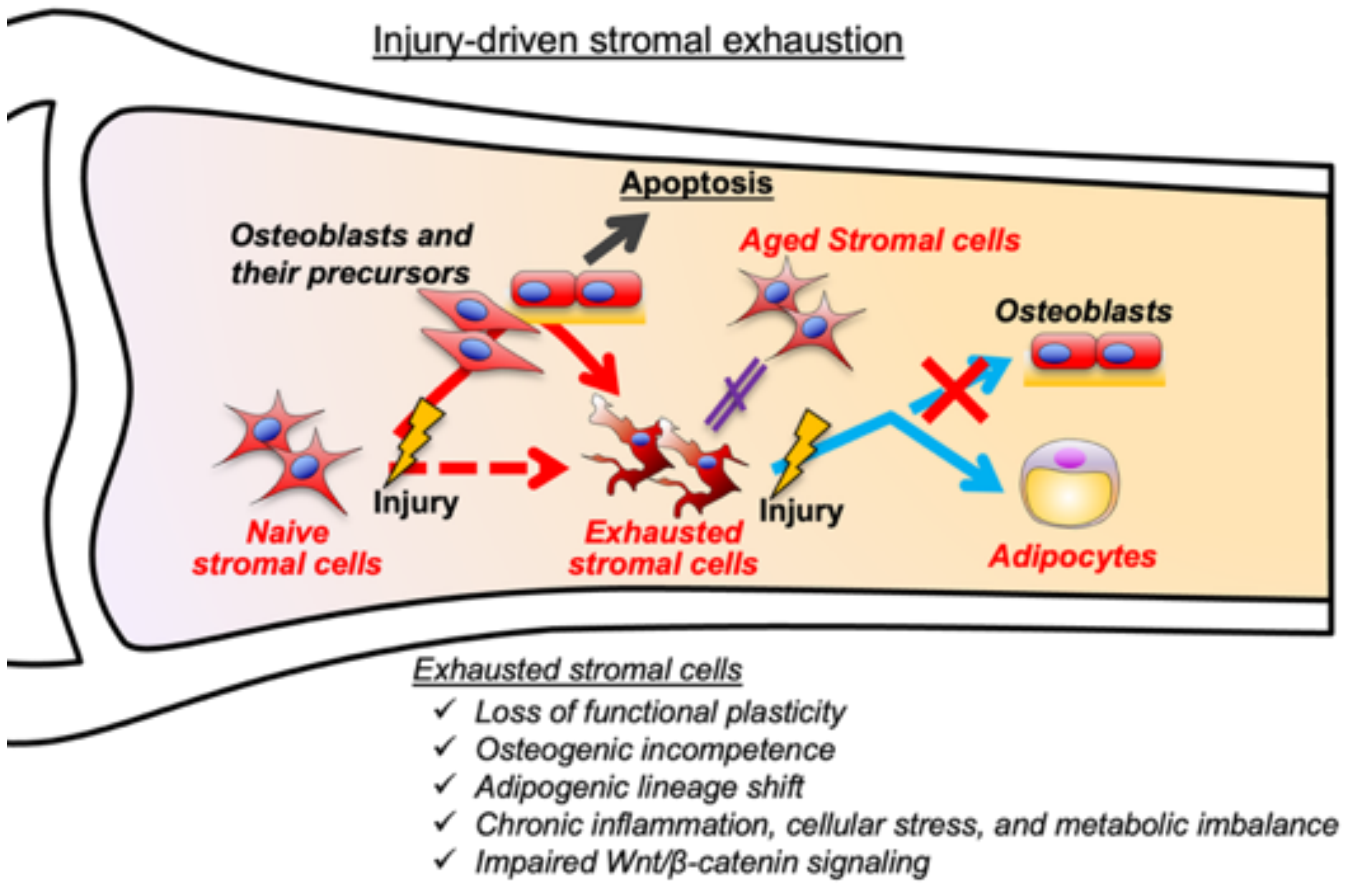

We are grateful for this constructive suggestion, which helped us improve both the conceptual precision and the presentation of the manuscript.

The following sentences have been added to the main text:

Line 196-197:

In the bone marrow, osteogenesis and adipogenesis are generally regarded as reciprocal processes

7. <Reviewer>

"Healthy" is probably not the best term to describe the comparator to the exhausted groups, as this appears to imply that the exhaustion state is a non-physiologic or "unhealthy" state. Exhaustion may represent an adaptive response in some cases. Perhaps "naive" or similar terminology would be more precise.

<Response>

Thank you very much for your suggestion. To avoid implying that the exhausted state is inherently "*unhealthy*," we replaced "*healthy*" with the more neutral and precise term "*naive*" (and adjusted related wording where appropriate) throughout the manuscript.

8. <Reviewer>

Line 177--it is not appropriate to term an in vitro CFU assay as informative regarding self-renewal. The CFU assay cannot discriminate proliferation occurring in concert with differentiation from true self-renewal.

<Response>

Thank you very much for your comments. We have revised the text accordingly.

The following sentences have been added to the main text:

Line 178-180:

Notably, the exhausted group exhibited fewer colonies than the control group, indicating ~~a severe decline in proliferation capacity and self-renewal potential~~ impaired proliferative and colony-forming capacity.

9. <Reviewer>

On what basis is cluster 7, line 287 termed periosteal? Pdpn is not sufficient to identify periosteal populations.

<Response>

Thank you very much for this comment. Because we newly added the Aged-injury group in the revised manuscript, we substantially updated the scRNA-seq plots and annotations. During this reanalysis, we annotated the cluster with high expression of *Postn*, *Acta2*, *Ctsk*, *Col3a1*, and *Pdpn* as periosteal cells (Cluster 7 in **Extended Data Figure 7a**). However, in the present subclustering analysis, we focused on skeletal cells within the bone and bone marrow compartments, taking anatomical context into account, and therefore excluded the periosteal cluster. Accordingly, the periosteal population has been removed from revised **Figure 7b**.

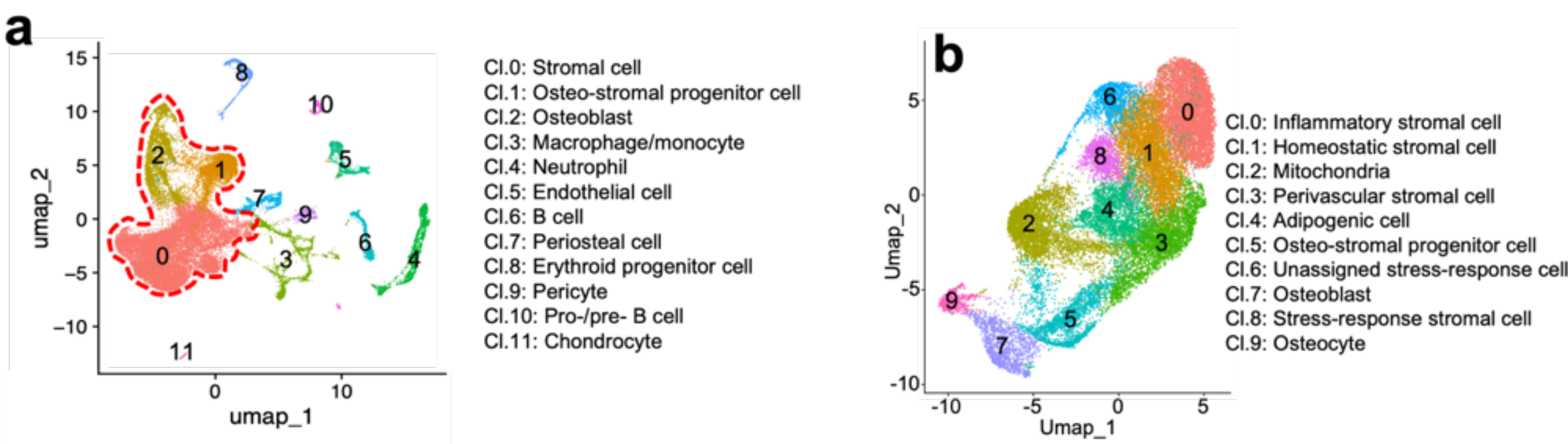

10. <Reviewer>

Line 349--are Fos and Nfkbia really markers of chronic inflammation? These genes have clear baseline physiological functions outside of inflammatory contexts.

<Response>

Thank you very much for your valuable comments. Following the reviewer's suggestion, we have removed *Fos* and *Nfkbia* from the list of chronic inflammation markers. In addition, we re-evaluated marker selection for inflammation, stress response, and adipogenic/lipid metabolism pathways to more accurately reflect the underlying biological processes. Furthermore, we revised the dot plot by including control samples, enabling a more robust, comparative visualization, as suggested by Reviewer 3. These revisions have improved the clarity and interpretability of the data. These data have been included as revised **Figure 8d**.

d

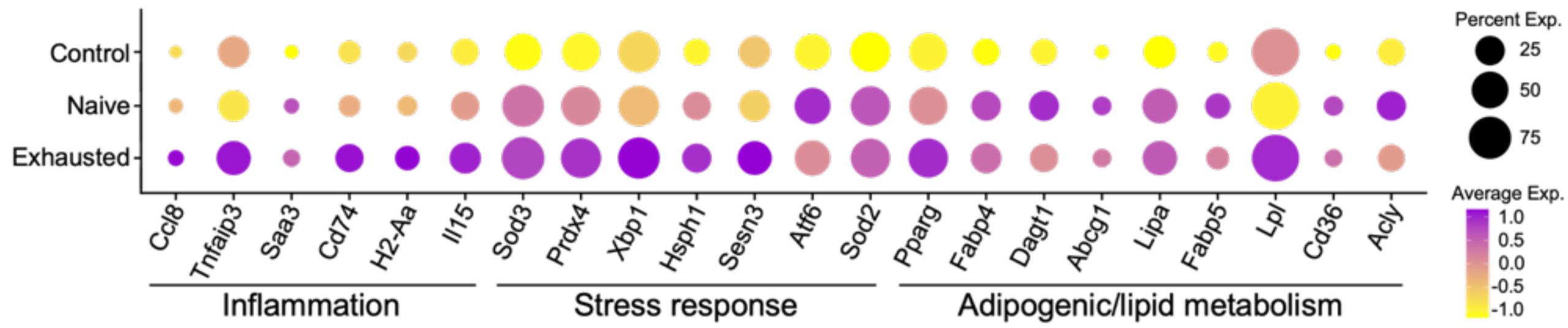

11. <Reviewer>

The results in Fig 2 would be easiest to interpret if it could be assumed that all of trabecular bone in the region of interest analyzed is newly formed after injury. A time=0 datapoint showing to what degree the trabecular bone in the region analyzed by uCT would be helpful. It is also requested that non-normalized versions of the uCT data in Fig 2c (the raw values, without dividing by the control group) be provided, especially for BV/TV.

<Response>

Thank you very much for your helpful comments. To directly clarify the extent to which trabecular bone in the analyzed region was newly regenerated after injury, we newly performed serial in vivo microCT analysis of the trabecular bone regeneration process after bone marrow ablation. Bone marrow ablation surgeries were performed at 9 weeks and 13 weeks of age, and the injured marrow area was evaluated at 1 day before 9 weeks (baseline), 1 day after 9 weeks (1st surg. +1d), 11 weeks (1st surg. +2w), 1 day before 13 weeks (1st surg. +4w; baseline before 2nd surg.), 1 day after 13 weeks (2nd surg. +1d), 15 weeks (2nd surg. +2w), and 17 weeks (2nd surg. +4w). These serial analyses showed that, after the first bone marrow ablation at 9 weeks, BV/TV decreased at 9w+1d, then markedly increased by 11 weeks, and returned to approximately the original baseline level by 13w-1d. After the second ablation at 13 weeks, BV/TV again decreased at 13w+1d, but showed only a slight increase by 15 weeks, indicating minimal trabecular bone regeneration compared with the response after the first injury. By 17 weeks, BV/TV had declined further to a level below the steady-state baseline (Extended Data Fig. 2). These findings indicate that trabecular bone is robustly regenerated after the first injury, but this regenerative response is markedly impaired after repeated injury. These data have been included as revised **Extended Data figure 3**.

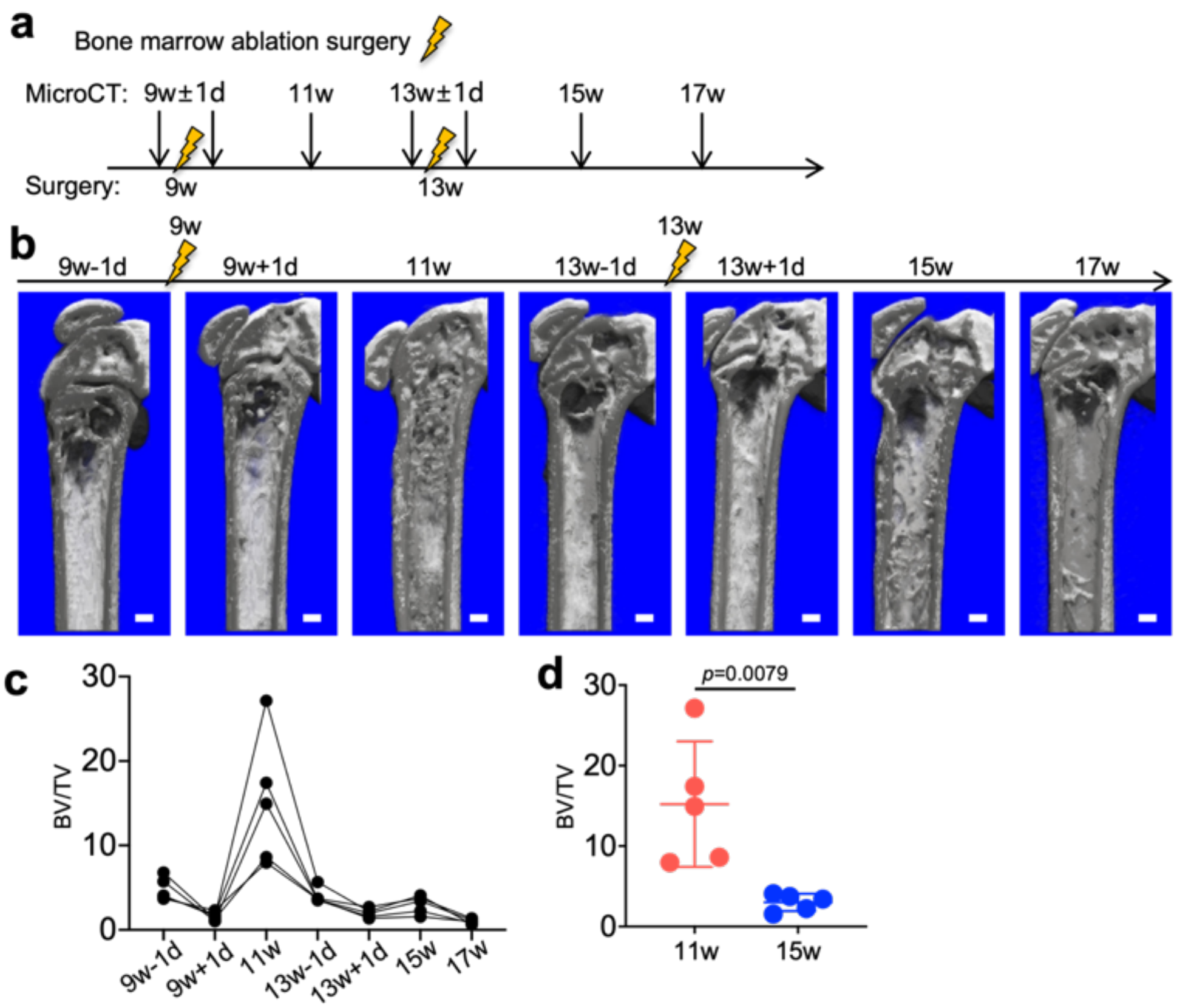

In addition, we have now provided the non-normalized microCT data in **Figure 1c**.

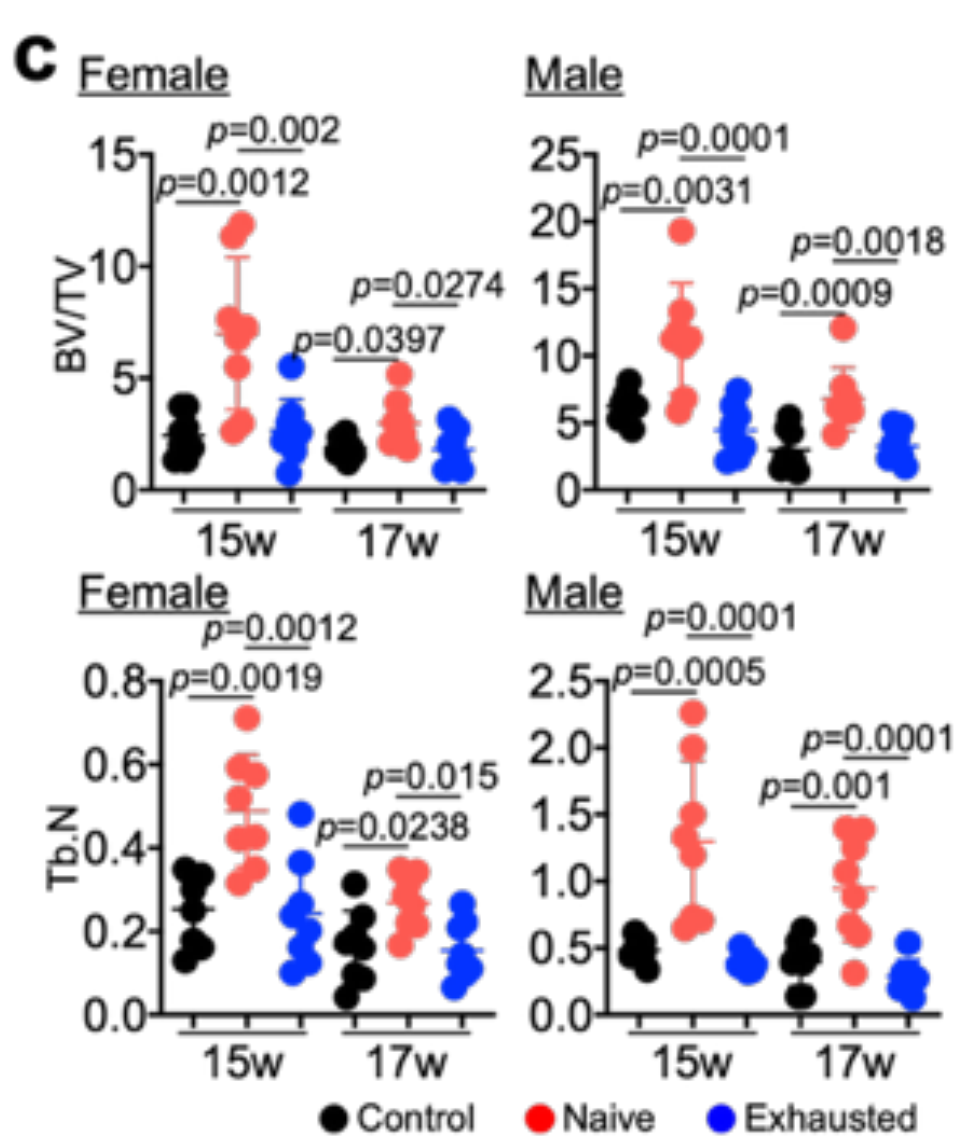

The following sentences have been added to the main text:

Line 162-165:

Serial in vivo micro-CT analysis including baseline time points further confirmed that trabecular bone in the analyzed region was robustly regenerated after the first ablation, whereas this regenerative response was markedly impaired after the second ablation (Extended Data Fig. 3a-c).

12. <Reviewer>

While the trajectory analysis in Fig 5 is useful, some qualification that trajectory analysis is inherently speculative should be made in the text. The authors may optionally consider briefly covering in the discussion the lineage tracing data that corroborates the model proposed based on trajectory analysis.

<Response>

Thank you very much for your comments. As Reviewer 2 and Reviewer 3 pointed out, trajectory analysis is useful but inherently speculative. Because the trajectory data is not fundamental in this manuscript and may cause readers to misunderstand, we removed the trajectory data.

Response to Reviewer #3

Thank you very much for your constructive comments and critiques.

0. <Reviewer>

The manuscript by Wu et al. explores the intriguing concept of stromal exhaustion in response to repeated skeletal injury as a potential root cause for impaired skeletal health and fragility. The authors conclude that mechanical injury impairs reticular cell function in the bone marrow preventing reversal to stromal cell states needed for injury responses. This is a well presented and written manuscript that uses male and female mice. However, the main claims of reversal of stroma-like cells, senescence, and exhaustion are not convincingly supported by provided data. Please refer to my specific comments below:

<Response>

Thank you very much for your critical and constructive assessment of our study.

1. <Reviewer>

ll.87 – 91: The authors mention endosteal and BM resident cells but not periosteal cells. For example, cited study by the Morisson group concluded contributions of periosteal cells. Please update that statement.

<Response>

Thank you very much for your comments. As the reviewer points out, multiple types of skeletal cells in periosteum and bone marrow contribute to bone regeneration. Because this manuscript only focuses on the regenerative process in bone marrow, we did not mention about periosteal contribution. But as the reviewer suggested, we agree that it is better to write the regenerative process accurately. We updated the statement and added the references.

The following sentences were added to the main text:

Line 82-87:

From a biological perspective, periosteal cells and bone-marrow mesenchymal cells coordinate to support bone regeneration by giving rise to osteoblasts and rebuilding skeletal tissue. Within the bone marrow environment, leptin receptor (Lepr)- and C-X-C motif chemokine ligand 12 (Cxcl12)-expressing reticular stromal cells in the central marrow space contribute to regeneration, particularly in adulthood, through injury-induced osteogenic differentiation and cellular plasticity

2. <Reviewer>

Figure 1b: Please annotate clusters. Please also provide individual plots for control and injured clustering to better assess overlap and differences.

<Response>

Thank you very much for your comments. We provided the cluster annotation and distinct UMAP plots of “Control” and “Injured”. We also moved this panel to **Extended data Figure 1b**, as suggested by reviewer 2.

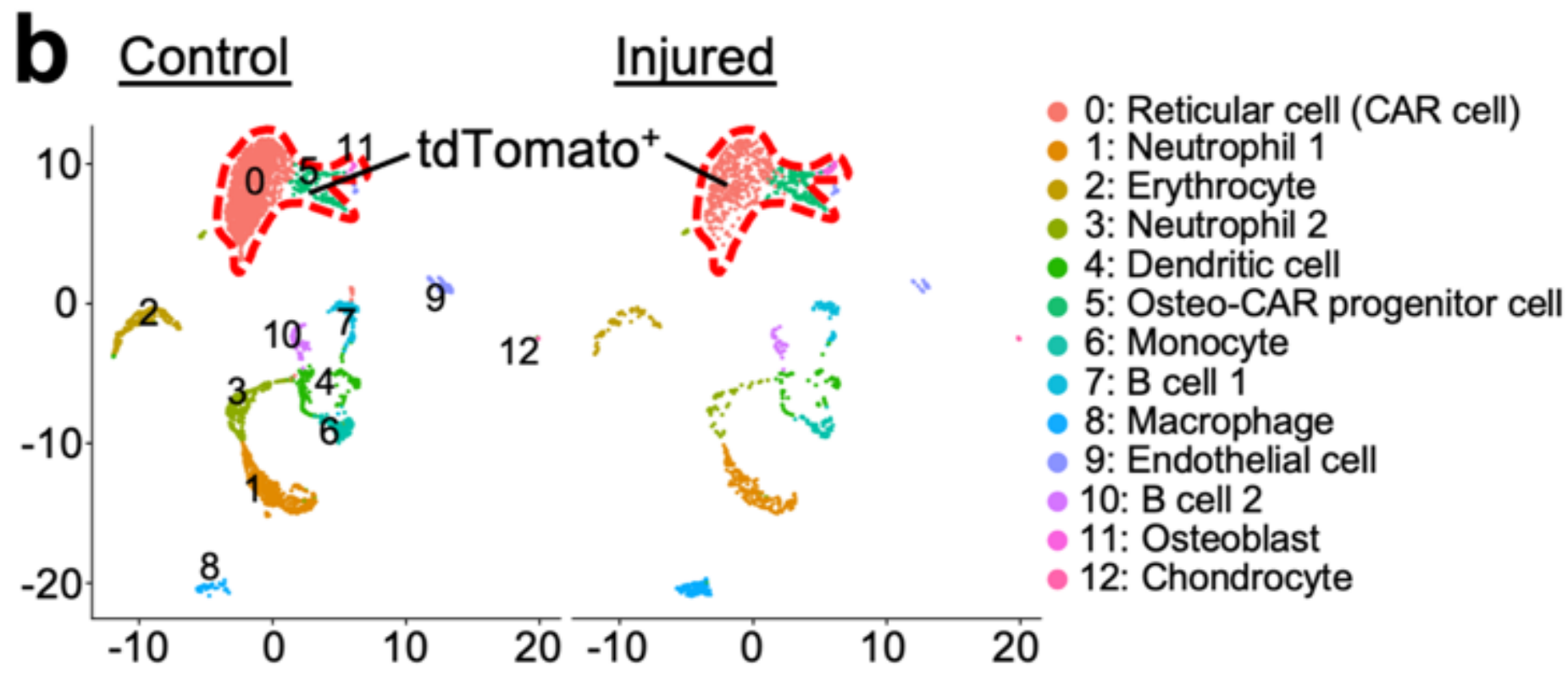

3. <Reviewer>

Figure 1d: Please provide dotplots for a broader set of markers. It seems quite selective as is. Is this for all cells or just some clusters?

<Response>

Thank you so much for the comment. The dot plots are generated specifically for the tdTomato+ clusters (Cl.0, 5, and 11), rather than for all cells. As the reviewer suggested, we also expanded the dot plots to include a broader panel of marker genes to provide a more comprehensive view of cluster identities. In addition, as noted above, we moved this panel to **Extended Data Figure 1c**.

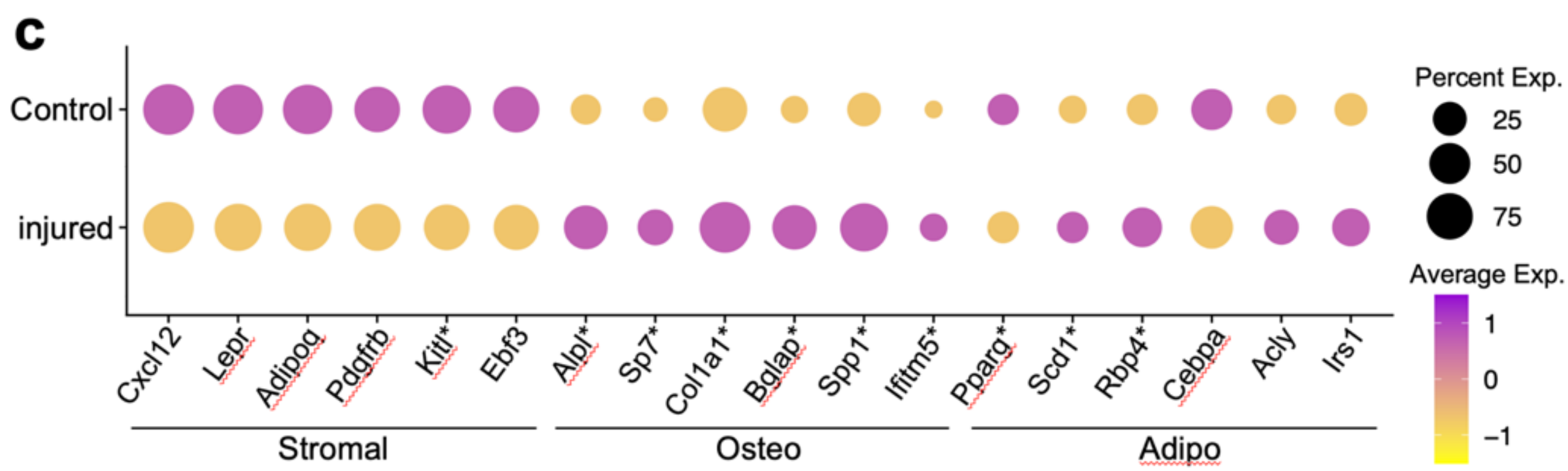

Overall, a single injury predominantly promotes osteogenic differentiation of stromal cells. Consistent with an osteogenic lineage shift, the expression of several stromal cell markers showed a modest decrease within the stromal population, although the changes were not statistically significant. In contrast, while adipogenic regulators such as *Pparg* were expressed at higher levels in the control condition and a clear induction of adipogenesis could not be concluded following a single injury, the increased expression of several adipogenic markers in the injured samples suggests that not all stromal cells uniformly shift toward osteogenesis.

The following sentences were added to the main text:

Line 136-138:

Interestingly, several adipogenesis-related genes, including Pparg, were expressed at higher levels in the control group, whereas others, such as Scd1 and Rbp4, were increased in the ablated group.

4. <Reviewer>

Extended data Fig.2: It appears the contribution of Lepr+ cells to total CFU-F is quite low. Are those Lepr- cells growth plate or periosteal cell “contaminations”?

<Response>

Thank you very much for your comments. During the cell preparation, we carefully remove growth plates and periosteal cells. In our previous data, interestingly, Lepr-cre$^+$ reticular stromal lineage cells in central marrow space contributed to only 3% of total CFU-Fs at P23 and around 50% at 8 weeks, instead, Fgfr3-creER endosteal stromal cells contribute almost 80% at P21 and 50% at 8weeks. The rate of Lepr-cre$^+$ cells in total CFU-Fs gradually increase along with aging (approximately 90% at 9 months) (**Figure b,g below**, Matsushita et al., Nat Commun, 2023). It is also proposed from other groups that the CFU-F-rich populations are located in the endosteal surface, not central marrow space (Siclari et al., Bone, 2013). Our current results consistent with those published data.

[Figure Redacted]

(Matsushita et al., Nat Commun, 2023)

5. <Reviewer>

Figure 2d suggests that there is more Lepr cell activity in periosteal areas in healthy group. Why would periosteal cells increase CFU-F in response to marrow ablation? Is the procedure consistent and highly reproducible?

<Response>

Thank you very much for your insightful comment. During the bone marrow ablation procedure, the cortical bone is briefly grasped with forceps to stabilize the femur, as shown in our previous study (**Figure 5b_v below**, Matsushita et al., Nat Commun, 2020). Although the cortex remains intact in this model, minor periosteal activation may occasionally occur due to surgical handling. In our experience, such periosteal responses are infrequent, occurring in 6.3% of cases (5 of 80 mice), and animals with obvious cortical damage or prominent periosteal/external callus-like reactions were not included in the main analyses. Importantly, because the cortical bone remains intact after ablation, this model is substantially less affected by periosteal disruption than fracture models, in which periosteal injury and callus formation are central components of the regenerative response. Thus, while a minor handling-related periosteal response may

explain the limited periosteal signal in revised **Figure 1d**, it is unlikely to account for the injury-responsive CFU-F activity observed in the marrow compartment.

Consistent with this interpretation, we previously showed that bone marrow ablation in Cxcl12-CreER mice (**Figure 5d-g below**, Matsushita et al., Nat Commun, 2020) reproducibly increased the proportion of injury-responsive Cxcl12-CreER$^+$ CFU-Fs (**Figure 6e,f below**, Matsushita et al., Nat Commun, 2020). scRNA-seq analysis further demonstrated that a subset of marrow Cxcl12$^+$Lepr$^+$ stromal cells undergoes injury-induced transcriptional changes toward a skeletal stem cell–like state after ablation (**Figure 7d,e below**, Matsushita et al., Nat Commun, 2020). We therefore consider marrow Cxcl12$^+$Lepr$^+$ stromal cells to be the major source of the increased CFU-F activity after injury.

[Figure Redacted]

(Matsushita et al., Nat Commun, 2020)

6. <Reviewer>

Figure 2 & Extended Data Fig.3: Overall, the authors mostly compare healthy with exhausted groups. It seems the exhausted phenotype more closely resembles the control group. It is unclear why this would suggest an impaired state. Do the increased BMAT fraction subside if bones were analyzed and compared at 20wks or longer after injury? It could be that the exhausted group might need additional times to recover.

Adipocytes are known to fill the initial void left by injury/ablation but can resolve. Do the authors observe the same after multiple bi-cortical fractures?

<Response>

Thank you very much for this thoughtful comment.

We appreciate the reviewer's careful observation that the Exhausted group appears closer to the Control group than to the Naive group in some aspects. We agree that this is an important point, and we believe that it directly supports the central concept of this study. In our model, Naive stromal cells, which have not previously experienced injury, respond to marrow ablation by activating a robust osteogenic regenerative program. In contrast, Exhausted stromal cells have already experienced a prior injury and returned to an apparently recovered baseline state, but upon re-injury they no longer show the same injury-induced osteogenic response. Thus, the resemblance between the Exhausted and Control groups is not a weakness of the phenotype; rather, it indicates that exhausted stroma has lost the capacity to re-engage the osteogenic program that is normally induced by injury. Therefore, we define stromal exhaustion not simply by an abnormal baseline histological appearance, but by a functional loss of injury-induced regenerative competence. In this sense, the similarity between Exhausted and Control groups provides important evidence that exhausted stromal cells no longer retain the injury-responsive osteogenic capacity observed in Naive stromal cells.

Consistent with this interpretation, in vivo microCT analysis showed that trabecular BV/TV after repeated injury tends to be reduced compared with controls. These data have been included as revised **Extended Data Figure 3**.

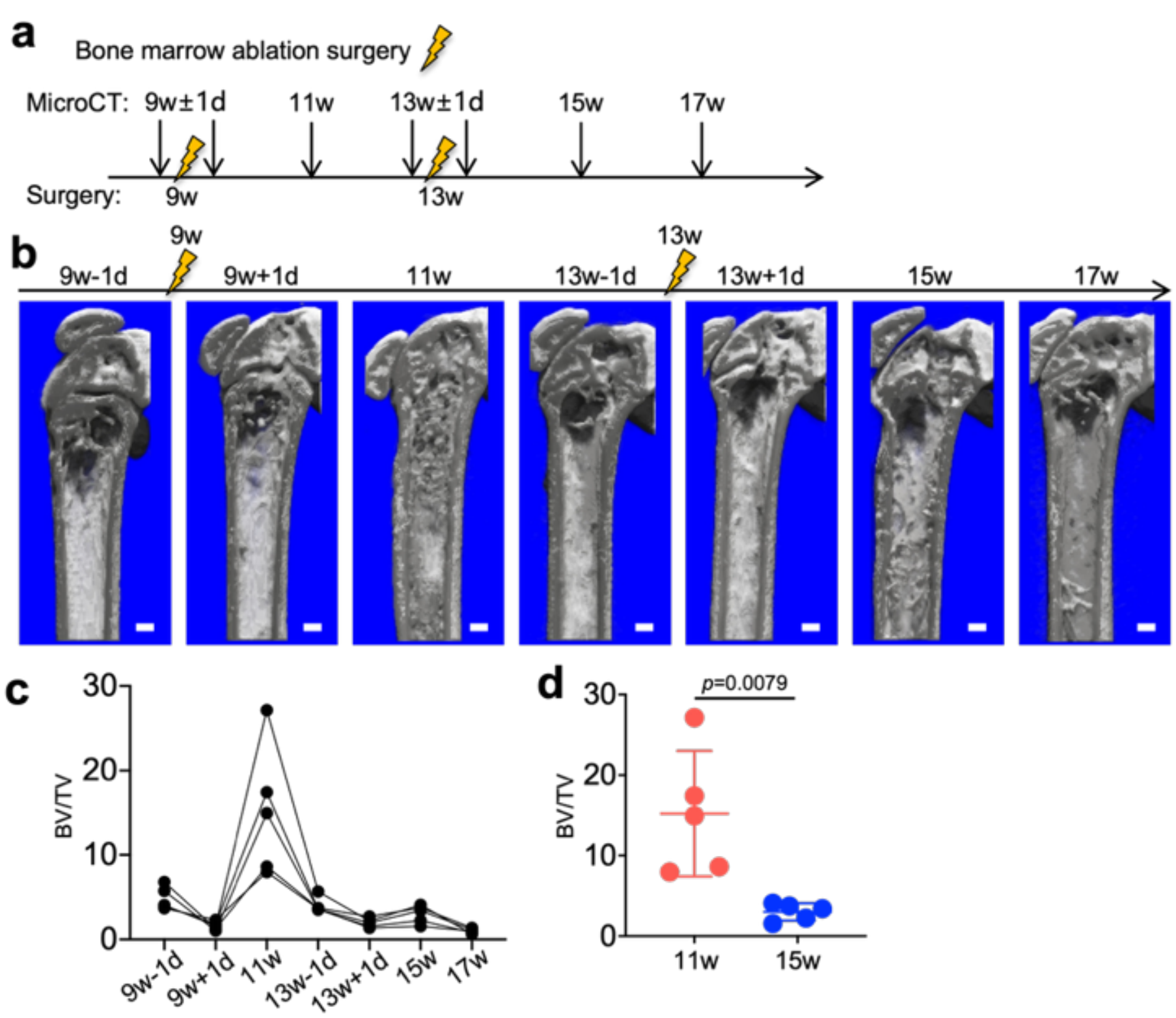

The following sentences have been added to the main text:

Line 162-165:

Serial in vivo micro-CT analysis including baseline time points further confirmed that trabecular bone in the analyzed region was robustly regenerated after the first ablation, whereas this regenerative response was markedly impaired after the second ablation (Extended Data Fig. 3a-c).

Furthermore, the phenotypic difference becomes even more apparent when examining bone marrow adiposity, as the Exhausted group shows a marked increase in adipocytes compared to controls. We appreciate the reviewer's observation that the Exhausted group appears similar to the Control group in some aspects. Indeed, radiographically, the Exhausted group resembles the Control group more closely than the Naive group. Histologically, the Exhausted group also resembles the Control group in terms of limited osteoblast formation. We consider this similarity to be informative, because it indicates that exhausted stroma no longer retains the injury-induced osteogenic capacity observed in Naive stroma. At the same time, the Exhausted group is not simply equivalent to Control, as it shows prominent adipocyte accumulation and clear molecular differences.

In response to Reviewer 2's comment, we re-evaluated marker selection for inflammation, stress response, and adipogenic/lipid metabolism pathways to better reflect the underlying biological processes. We also followed Reviewer 3's recommendation to directly compare the three conditions—Control, Naive, and Exhausted—which allowed us to better resolve their differences. Specifically, single-cell RNA-seq analysis revealed that markers associated with inflammation, stress response, and adipogenic/lipid metabolism are markedly upregulated in the Exhausted group compared with the Control condition (revised **Figure 8d**). Thus, exhausted stroma represents a radiographically Control-like but biologically distinct state, characterized by limited osteogenic reactivation, enhanced adipogenic remodeling, and inflammatory/stress-associated molecular signatures.

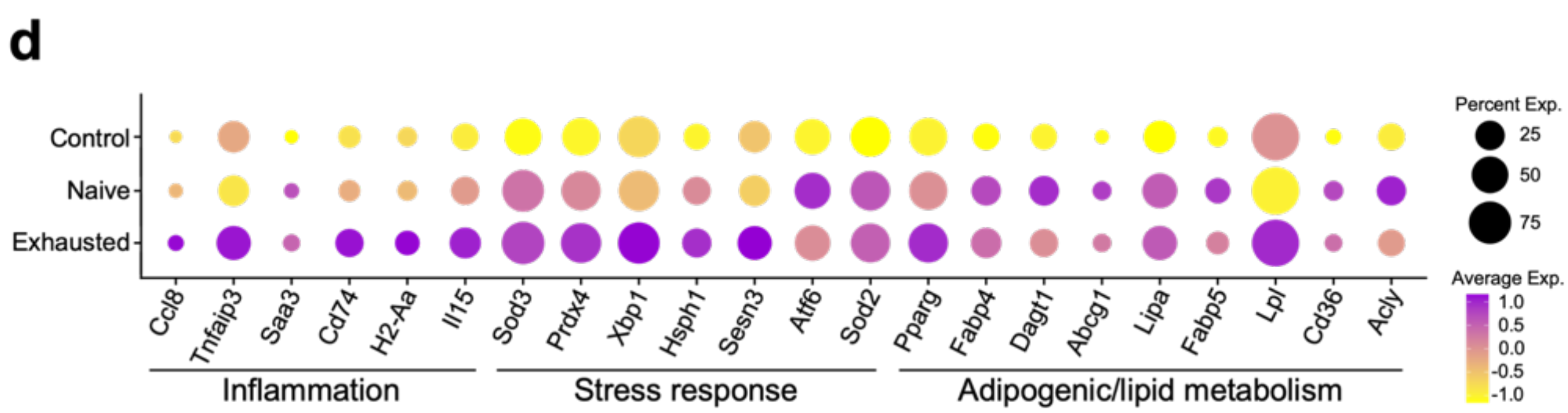

With respect to Wnt/β-catenin signaling, Lepr^{+} stromal cells do not show strong activation at baseline but normally activate this pathway in response to injury. Notably, in the Exhausted condition, this expected injury-induced Wnt/β-catenin activation is absent. Instead, these cells exhibit a transcriptional state more similar to the non-activated Control condition, despite prior injury exposure (revised **Figure 8e,f**).

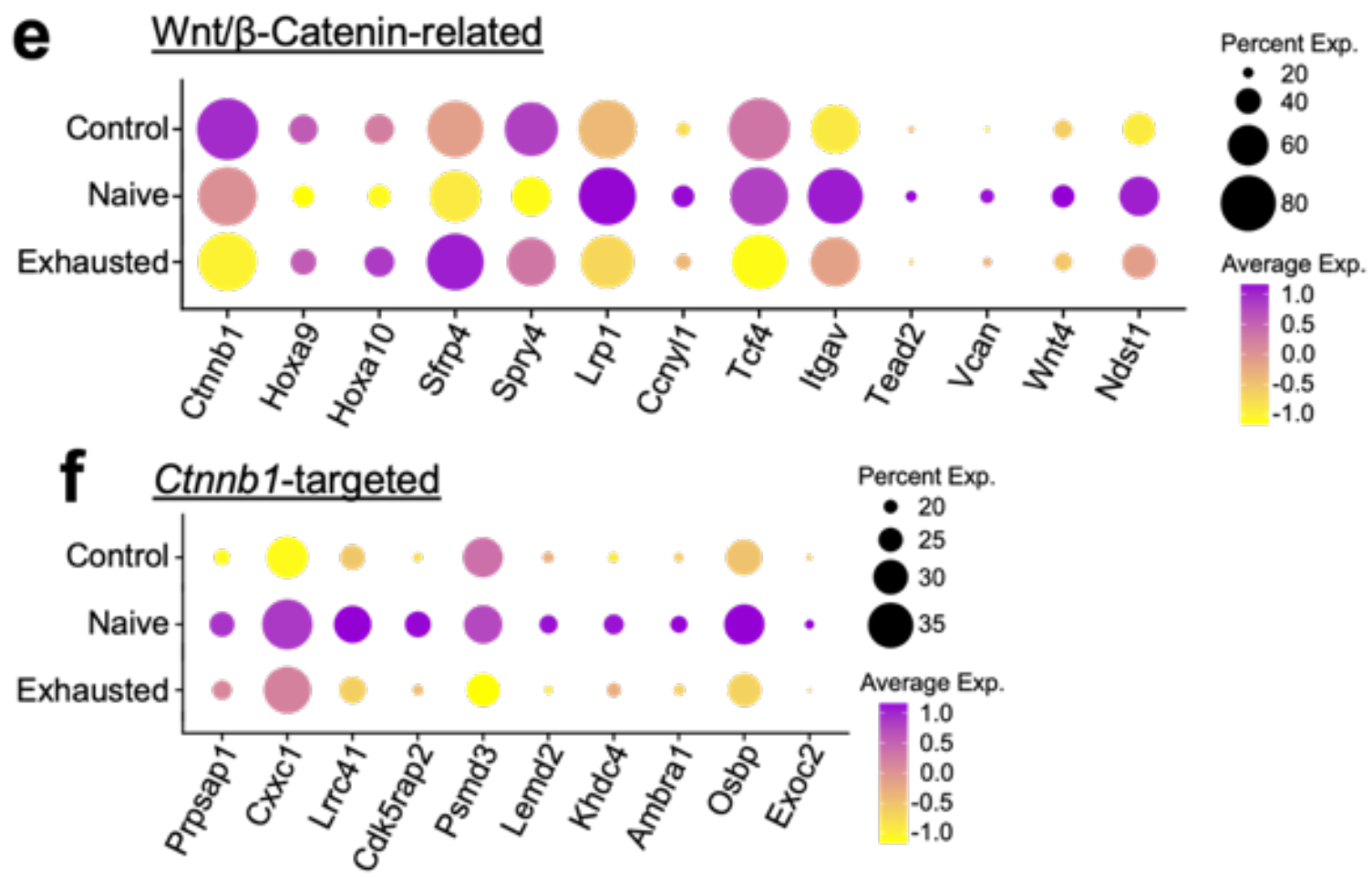

We consider this failure to appropriately reactivate Wnt/β-catenin signaling upon injury to be a key feature of stromal exhaustion, and a central aspect of the phenotype described in this study.

We agree that the temporal dynamics of BMAT and the possibility of delayed recovery are important points to consider. To address this issue more directly, we further examined the time course after a single bone marrow ablation. Following marrow ablation at 9 weeks of age, adipocytes were increased at 15 weeks of age, whereas adipocyte accumulation was reduced by 21 weeks of age. These observations indicate that injury-induced BMAT can subside over time after a single injury. This reduction may reflect potential reversibility of adipocytes toward stromal-like cells and/or clearance mechanisms such as apoptosis.

Importantly, however, the resolution of adipocyte accumulation did not indicate recovery of injury-induced osteogenic regenerative capacity. To directly test whether stromal cells recover their regenerative function after a prolonged interval, we performed a second marrow ablation at 21 weeks of age, following the first ablation at 9 weeks of age. In the Long-term group, mice that received a single marrow ablation at 9 weeks and were analyzed at 21 weeks showed a histologically restored marrow space, with reticular stromal cells re-established and adipocyte accumulation largely resolved. In contrast, when a second injury was performed after this prolonged recovery period, the Long-term rechallenged group showed minimal bone regeneration and a marked shift toward adipocyte differentiation, in stark contrast to the robust osteogenic response observed after the first injury in the Naive group. These data have been included as revised **Figure 3d–h**.

These findings indicate that, although injury-induced adipocyte accumulation can subside after a single injury, stromal cells do not regain their injury-induced osteogenic regenerative capacity. Thus, stromal exhaustion is not defined simply by persistent adipocyte accumulation at baseline, but rather by a sustained loss of osteogenic competence upon subsequent injury.

We also acknowledge the important point that BMAT can be reversible in some contexts. For example, irradiation-induced bone marrow adipocytes have been reported to reacquire stromal characteristics and contribute to bone regeneration (Hirakawa et al., Nat Genet 2023). In contrast, our data

suggest that after repeated physical injury, stromal cells remain functionally exhausted even after apparent resolution of adipocyte accumulation. This difference between irradiation and mechanical injury is intriguing and may reflect distinct forms of injury memory or stromal dysfunction, potentially involving mechanisms such as epigenetic remodeling. Although defining these mechanisms is beyond the scope of the present study, we have incorporated this point into the Discussion as an important direction for future investigation.

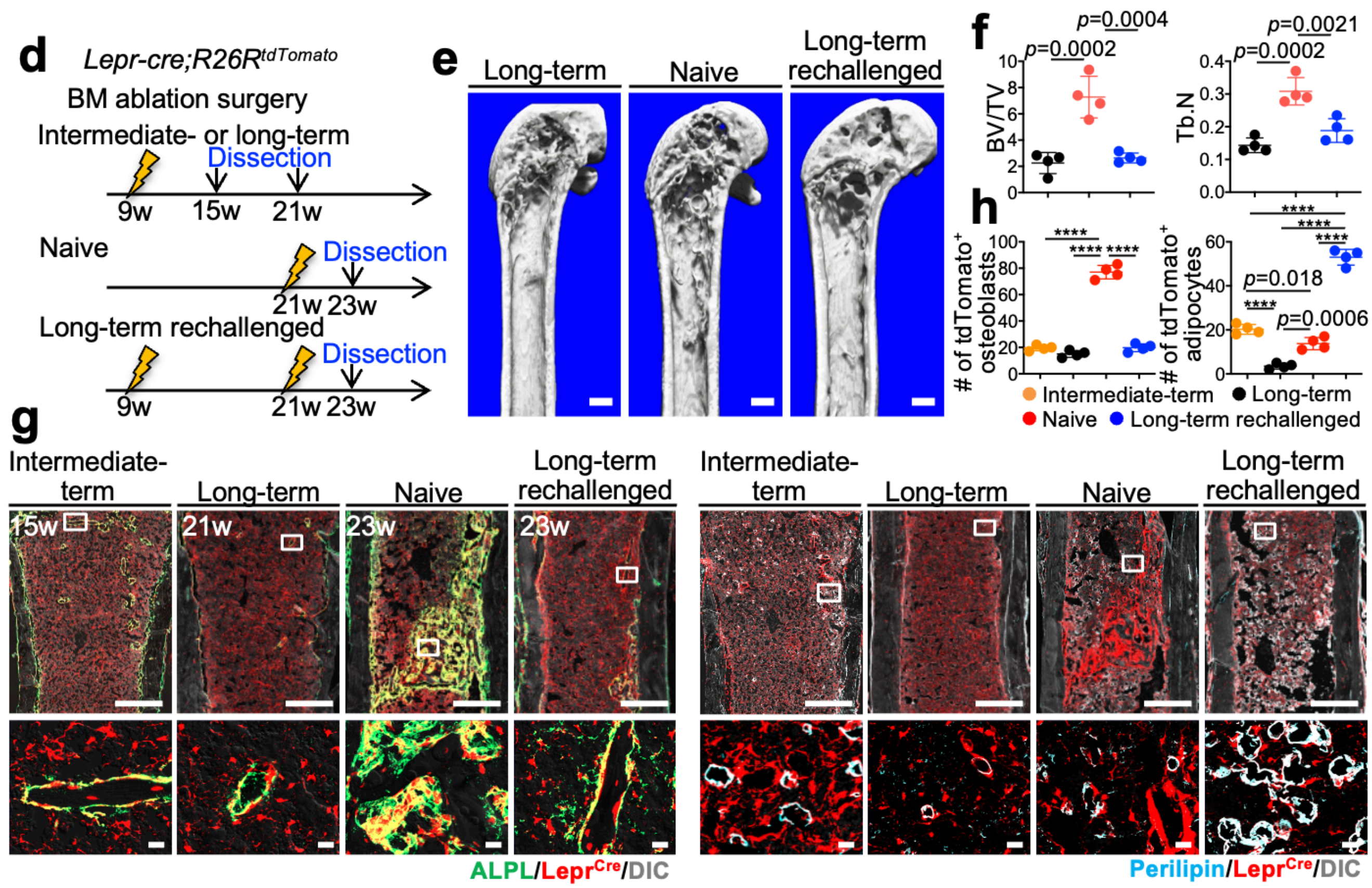

We also addressed whether a similar phenomenon occurs after repeated fractures. To this end, we performed additional experiments using a stabilized femoral fracture model in *Lepr-cre*; *R26R*^{tdTomato} mice. This model closely mimics clinical fracture treatment, in which an intramedullary pin is inserted and bone healing is monitored. Notably, after repeated fractures, we observed reduced bone regeneration, decreased Tomato^{+} osteoblasts, and increased Tomato^{+} adipocytes, consistent with our findings in the bone marrow ablation model. Although periosteal cells are known to be a major source of fracture repair, these results indicate that marrow stromal cells are also significantly affected by repeated physical injury. These data have been included as revised **Figure 3a–c.**

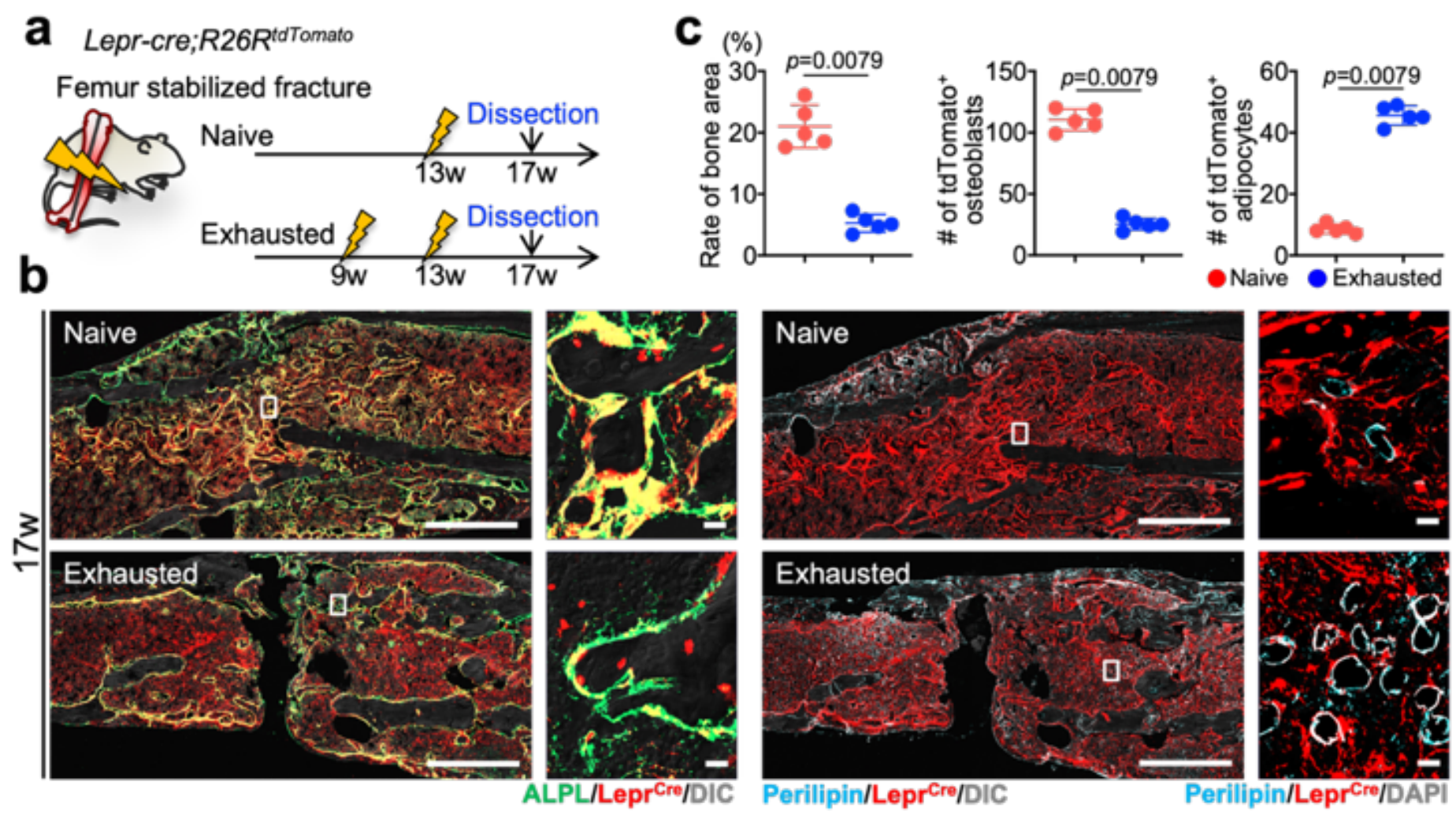

The following sentences were added to the main text:

Line 235-265:

4. Stromal exhaustion occurs in fracture repair and persists after prolonged recovery

To determine whether stromal exhaustion also occurs during fracture repair, we next examined LeprCre+ lineage cells using a femur stabilized fracture model (Fig. 3a). In this model, repeated fracture resulted in a reduced amount of newly formed bone and a decreased contribution of LeprCre+ lineage cells to osteoblasts at the repair site (Fig. 3b,c). In contrast, LeprCre+ lineage cells showed an increased contribution to marrow adipocytes after repeated fracture (Fig. 3b,c). These findings are consistent with the phenotype observed after repeated bone marrow ablation surgery and indicate that stromal exhaustion is not specific to the ablation model, but can also occur during fracture repair.

We further examined whether the four-week interval after the first injury was insufficient for recovery and whether stromal exhaustion reflected a transient delay rather than a sustained functional alteration. To address this, we performed a long-term rechallenge experiment, with a 12-week interval between the first and second bone marrow ablation surgeries (Fig. 3d). Micro-CT analysis revealed robust trabecular bone regeneration in the naive group, whereas the long-term rechallenged group showed markedly reduced trabecular bone formation after the second injury (Fig. 3e). Quantitative analysis confirmed significantly lower BV/TV and trabecular number in the long-term rechallenged group than in the naive group (Fig. 3f).

We then examined the histological changes underlying this impaired regenerative response. After a single marrow ablation surgery at 9 weeks of age, marrow adipocytes were modestly increased by 15 weeks of age but had largely subsided by 21 weeks of age, by which time the marrow cavity appeared largely histologically restored, with re-established reticular stromal cells and limited residual adipocyte accumulation (Fig. 3g,h). Importantly, however, this apparent histological recovery did not indicate restoration of injury-induced osteogenic regenerative capacity. In the long-term rechallenged group, LeprCre+ lineage cells exhibited a markedly reduced osteogenic response and enhanced adipogenic differentiation after the second injury (Fig. 3g,h).

These findings indicate that, although injury-induced adipocyte accumulation can subside after a single injury, stromal cells do not regain their injury-induced osteogenic regenerative capacity. Thus, stromal exhaustion is not merely a transient delay in recovery or persistent adipocyte accumulation at baseline, but rather reflects a persistent functional alteration of injury-experienced marrow stromal cells.

Line 514-516:

Although bone marrow adipocytes can exhibit reversibility under certain conditions, such as after irradiation, our findings suggest that repeated mechanical injury may induce a distinct, less reversible form of stromal dysfunction.

7. <Reviewer>

Figure 3 and Extended Data Fig 3e-g: It appears from images that the majority of BMAT is not Cxcl12-dTomato derived. Please provide individual fluorescence channels. Are those CAR cell derived adipocytes generated from SSC-generated stromal cells after Tamoxifen induction? Is that why images look like there is a significant difference in adipocyte number between Control and Healthy groups but the Fig.3c quantifications on tomato+ adipocytes does not show any difference (at least female looks significant)?

<Response>

Thank you very much for your comments. We apologize that we only showed the merged images. We show the individual fluorescence channels **below**. We would like to clarify that the majority of BMAT observed in our system is derived from Cxcl12-creER$^+$ or Lepr-cre$^+$ stromal cells. In the Cxcl12-creER model, tamoxifen induction preferentially labels Adipo-CAR cells (Matsushita et al., Nature Communications 2020), allowing us to trace their adipogenic fate.

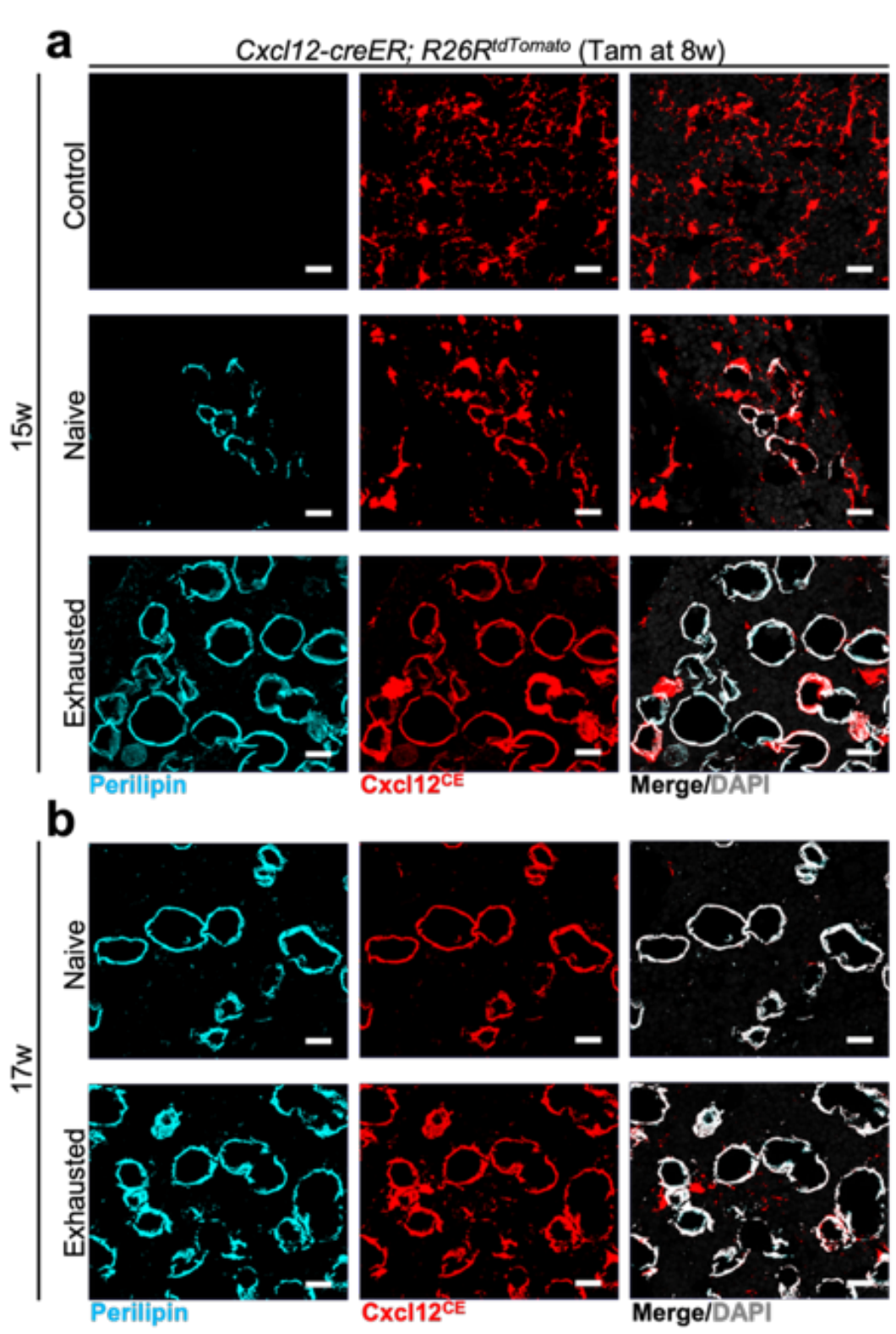

Although the numbers of tdTomato⁺ adipocytes between Control and Naive (former Healthy) groups in Fig. 2c (Lepr-cre mice, injury +2w, 15w of age) do not reach statistical significance, there is a clear trend toward an increase in the Naive group (female: $p = 0.054$; male: $p = 0.15$). Therefore, the visual impression from the images is consistent with the quantitative analysis rather than discrepant. Consistently, Fig. 2 and Fig. 4e–g indicate that bone marrow adipogenesis gradually increases after marrow ablation surgery. We also provide the original individual data **below** at 15 weeks of age in *Lepr-cre*; *R26R*^{tdTomato} mice to further support this trend.

Number of tdTomato+ adipocytes

	Female (Dis15w)		
Sample#	Control	Naive	Exhausted
1	0	121	208
2	0	86	334
3	0	115	455
4	7	73	392
5	14	81	530
	Male (Dis15w)		
Sample#	Control	Naive	Exhausted
1	0	170	329
2	2	53	176
3	0	17	131
4	2	38	270
5	0	103	362

8. <Reviewer>

p16INK4a by itself (and the expression of Cdkn2a and Cdkn1a) is not sufficient to claim senescent state (https://www.cell.com/cell/fulltext/S0092-8674(24)00640-8). Upregulation of these markers could just reflect an "emergency" state activated for proper resolution of injury. Additional assays should be performed, e.g., IHC for other markers and beta-Gal stain for stromal cells.

<Response>

Thank you very much for this important comment. We agree that the expression of p16INK4a and related markers alone is not sufficient to conclusively define a senescent state. To address this concern, we performed additional histological analyses using SA-β-gal staining, which revealed an increased presence of senescent cells in the exhausted group. These data have been included as revised **Extended Data Figure 5c and Figure 2e**.

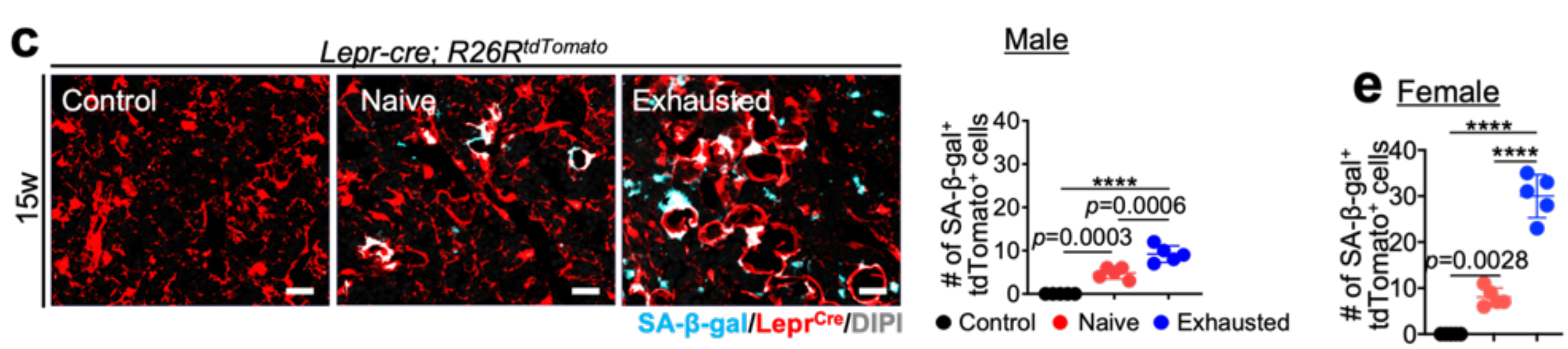

Furthermore, to strengthen our conclusions from an independent perspective, we utilized *p16-creER*; *R26R*^{tdTomato} mice to genetically label senescent cells and their lineages. In these experiments, tamoxifen was administered after the second injury to label cells that activated the p16 program at that time point. This approach allowed us to visualize cells that entered a p16-expressing state following repeated injury and to examine their presence within the exhausted microenvironment. Using this strategy, we confirmed an increased accumulation of p16-labeled cells in the exhausted group (revised **Figure 2h**).

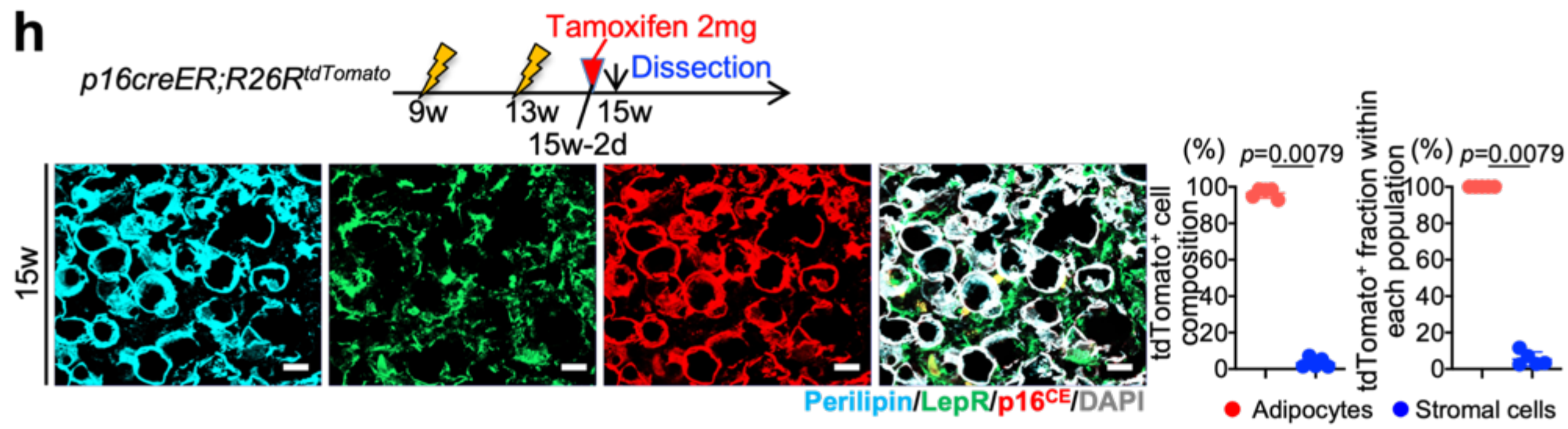

In addition, as suggested by the reviewer in comment 10, we further examined whether the senescent cells were stromal cells or adipocytes. Interestingly, these analyses revealed that stromal cells themselves do not exhibit substantial senescence. Instead, the increase in senescent cells in the exhausted group appears to be largely attributable to adipocytes. Together with the senescence-associated transcriptional signatures observed in our scRNA-seq dataset, these independent lines of evidence consistently support the presence of increased senescent cells in the exhausted microenvironment (revised **Figure 2g**).

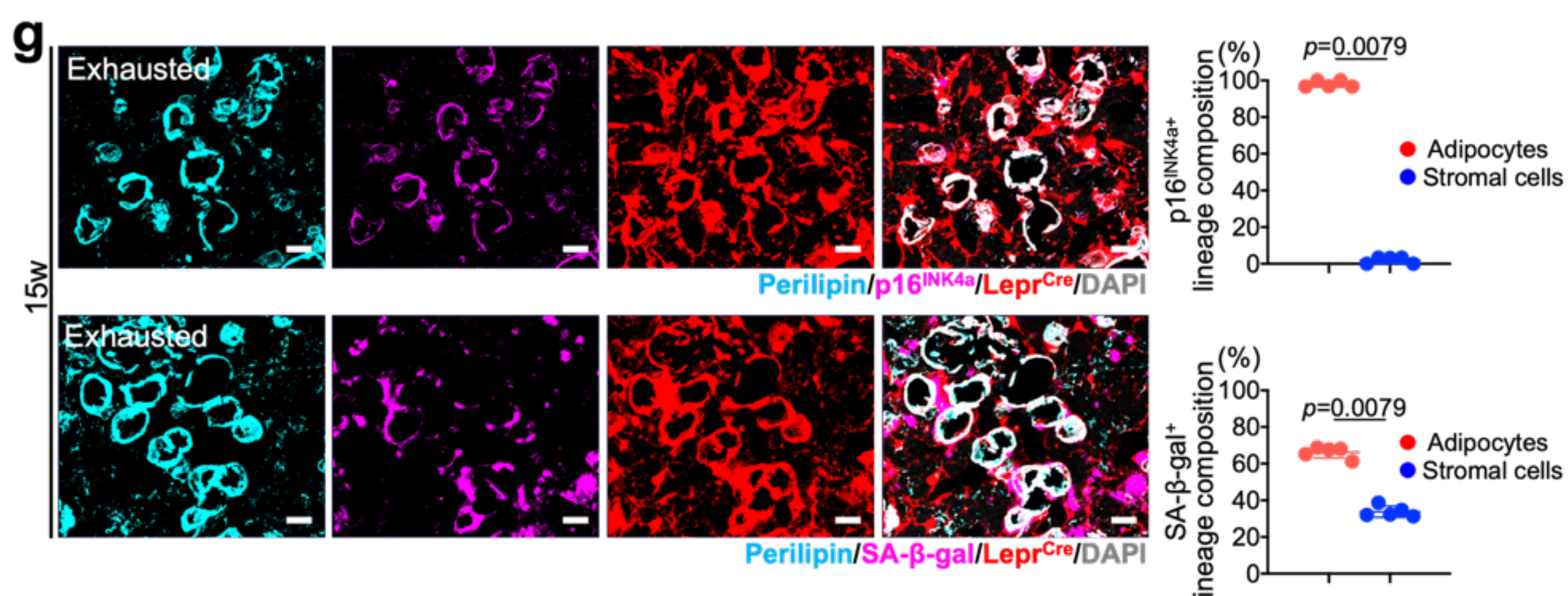

Importantly, these findings suggest that the exhausted phenotype cannot be explained solely by intrinsic senescence of stromal cells, but rather reflects broader alterations in the cellular composition and microenvironment following repeated injury.

The following sentences were added to the main text:

Line 213-215:

we performed immunofluorescence staining for the senescence-associated marker p16INK4a and SA-β-gal staining. Two weeks post-surgery, LeprCre-tdTomato+p16INK4a$^+$ and LeprCre-tdTomato+ SA-β-gal$^+$ senescent cells were increased in the exhausted group

Line 219-233:

However, further histological analysis revealed that p16INK4a and SA-β-gal signals were predominantly detected in perilipin$^+$ adipocytes rather than in LeprCre-tdTomato$^+$ stromal cells (Fig. 2g). Consistently, p16-creER; R26R^{tdTomato} lineage cells labeled after the second injury showed that p16+ cells largely overlapped with adipocytes, whereas only a limited fraction overlapped with LepR$^+$ stromal cells (Fig. 2h). These findings suggest that, although senescence-associated cells accumulate in the exhausted marrow environment, the exhausted phenotype of stromal cells is not simply explained by classical cellular senescence of the stromal population itself.

In summary, our findings demonstrate that exhausted bone marrow stromal cells undergo significant adipogenic differentiation ~~and increased senescence post-injury~~, leading to elevated marrow fat accumulation. ~~Moreover, cellular senescence may underlie injury-driven stromal exhaustion and potentially contribute to impaired bone regeneration and marrow homeostasis.~~ Although senescence-associated cells increased in the exhausted marrow environment, these signals were predominantly associated with adipocytes rather than stromal cells themselves. Thus, the exhausted phenotype is characterized primarily by adipogenic conversion and loss of stromal regenerative capacity, rather than being simply explained by classical cellular senescence of stromal cells.

9. <Reviewer>

Figure 4: The experimental setup does not allow for conclusions about reversion to stromal state as this can only be done if individually cells/clones were traced. Stromal fraction likely increases rather than OSX+ cells reverting. Reduction of OSX cells might also be driven by apoptosis. The conclusion is therefore not warranted and should be revised or demonstrated with additional experiments.

<Response>

Thank you very much for this important comment. As the reviewer correctly pointed out, our current analysis time points do not allow us to strictly trace the Osx lineage. Even in the BM ablation +2 week samples, there is a ~10-day interval after the initial tamoxifen administration, which prevents precise lineage tracing of Osx-derived cells.

To address this limitation, we analyzed Osx-creER–labeled cells at earlier stages of the bone regeneration process. Specifically, we examined the expression profile of Osx-creER$^+$ cells immediately after labeling during the early phase (tamoxifen injection at day 4, dissection at day 6) and the intermediate phase (tamoxifen injection at day 13, dissection at day 15) of regeneration. In both phases, Osx-creER$^+$ cells showed minimal expression of LepR. These findings indicate that Osx-creER predominantly labels

osteoblasts and their precursor populations, and that these cells can potentially give rise to LepR$^+$ cells during the regenerative process. These data have been included as revised **Extended Data Figure 6**.

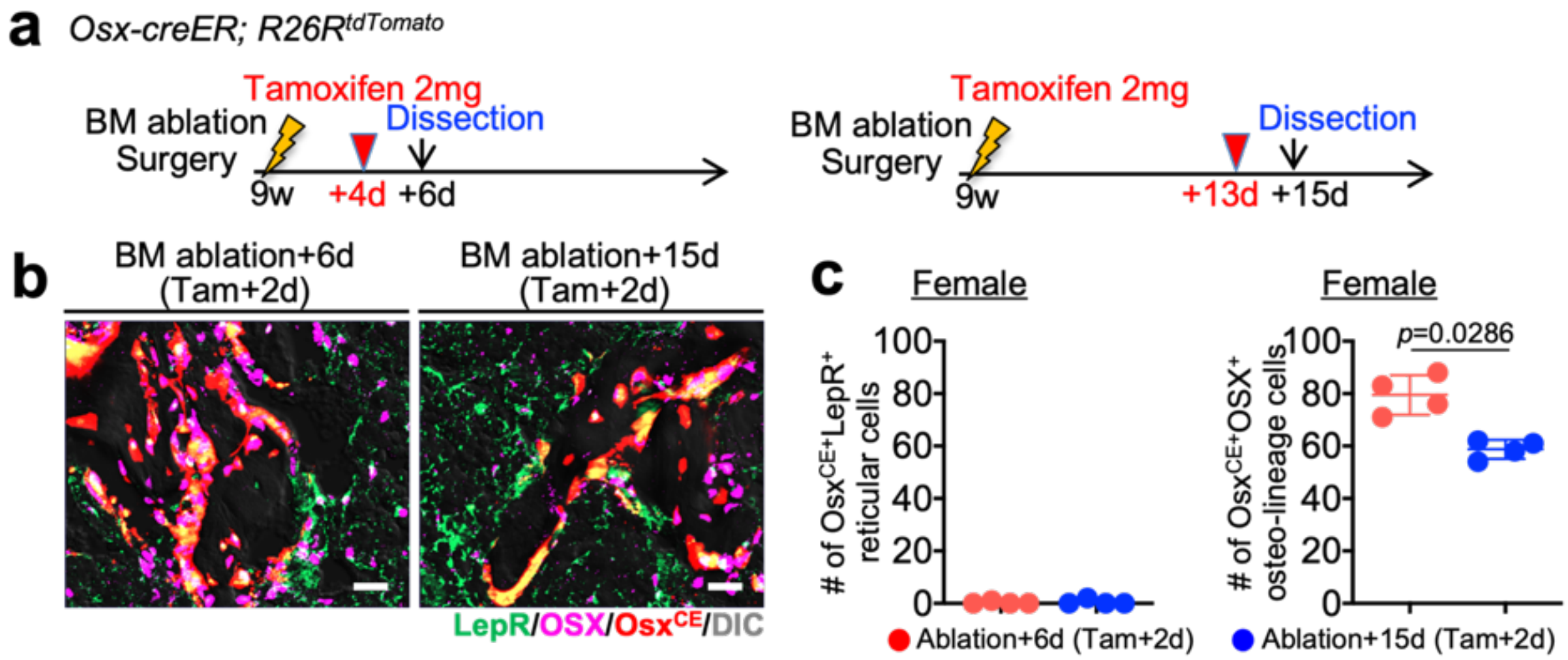

This observation is consistent with previous lineage-tracing studies demonstrating that Osx-creER–labeled cells at early postnatal stages (P5), which initially show minimal overlap with LepR$^+$ cells, subsequently give rise to many LepR$^+$ stromal cells by adulthood (15 weeks) (Mizoguchi et al., Dev Cell, 2014).

However, as the reviewer suggested, we cannot exclude the possibility that a small population of LepR$^+$Osx-creER$^+$ cells expands during the repair process. Indeed, stromal cells are known to proliferate in response to injury, as we previously reported (Matsushita et al., Nat Commun, 2020). In addition, our data indicate that a fraction of Osx-creER$^+$ cells undergo apoptosis during this process. Accordingly, we have revised the manuscript and revised **Figure 11** to avoid a unidirectional interpretation of reversion and to explicitly present multiple possible fates of regenerating osteogenic cells, including apoptosis, persistence in the osteogenic lineage, and possible return to a stromal state.

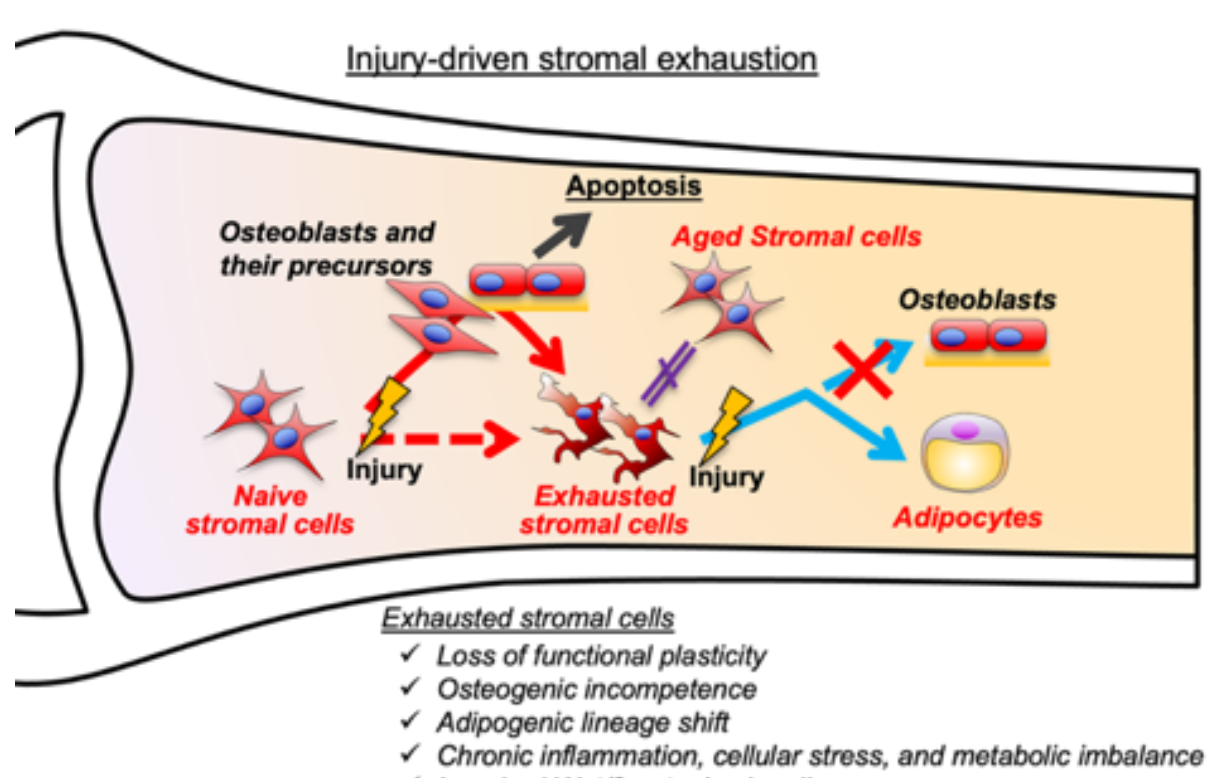

The following sentences were added to the main text:

<u>*Line 298-304:*</u>

To define the initially labeled Osx-creER$^+$ population, we performed short-chase analyses during regeneration. When tamoxifen was injected at day 4 or day 13 after injury and femurs were dissected 2 days

later, Osx-creER$^+$ cells predominantly overlapped with OSX$^+$ osteogenic cells, whereas only a minimal fraction overlapped with LepR$^+$ stromal cells (Extended Data Fig. 6a-c). At 2 weeks post-surgery, OsxCE$^+$ cells were still predominantly labeled as OSX$^+$ osteogenic cells, but the fraction contributing to LepR$^+$ stromal cells was slightly increased compared with the short-chase analysis.

Line 312-314:

These findings suggest that regenerating osteogenic cells undergo multiple fates, including apoptosis, reversion to the stromal state, and persistence in the osteogenic lineage.

Line 323-326:

Together, these results suggest that regenerating osteogenic cells exhibit multiple possible fates, including apoptosis, persistence within the osteogenic lineage, and potential return to a stromal-like state. Such stromal-like cells may become functionally altered and more susceptible to exhaustion, thereby limiting regenerative capacity after repeated injury.

Line 509-510:

Although these stromal cells initially differentiate into osteoblasts and their precursor cells upon injury,

10. <Reviewer>

Fig4G suggests that mainly Adipocytes are labeled by p16INK4a, but not other LepR cells, which would not align with the authors conclusion that stromal cells become senescent.

<Response>

Thank you very much for this insightful comment. As the reviewer pointed out, Fig. 4G suggests that p16INK4a labeling is predominantly observed in adipocytes rather than in Lepr-cre–labeled stromal cells. We agree that this observation requires careful interpretation and provided an important clue for refining our interpretation of the exhausted bone marrow phenotype.

To clarify the cellular identity of senescent cells, we first performed p16INK4a immunostaining together with SA-β-gal staining. These analyses showed that classical senescence markers were only weakly detected in Lepr-cre–labeled stromal cells (**Figure 2g**), whereas adipocytes displayed much stronger senescence-associated signals.

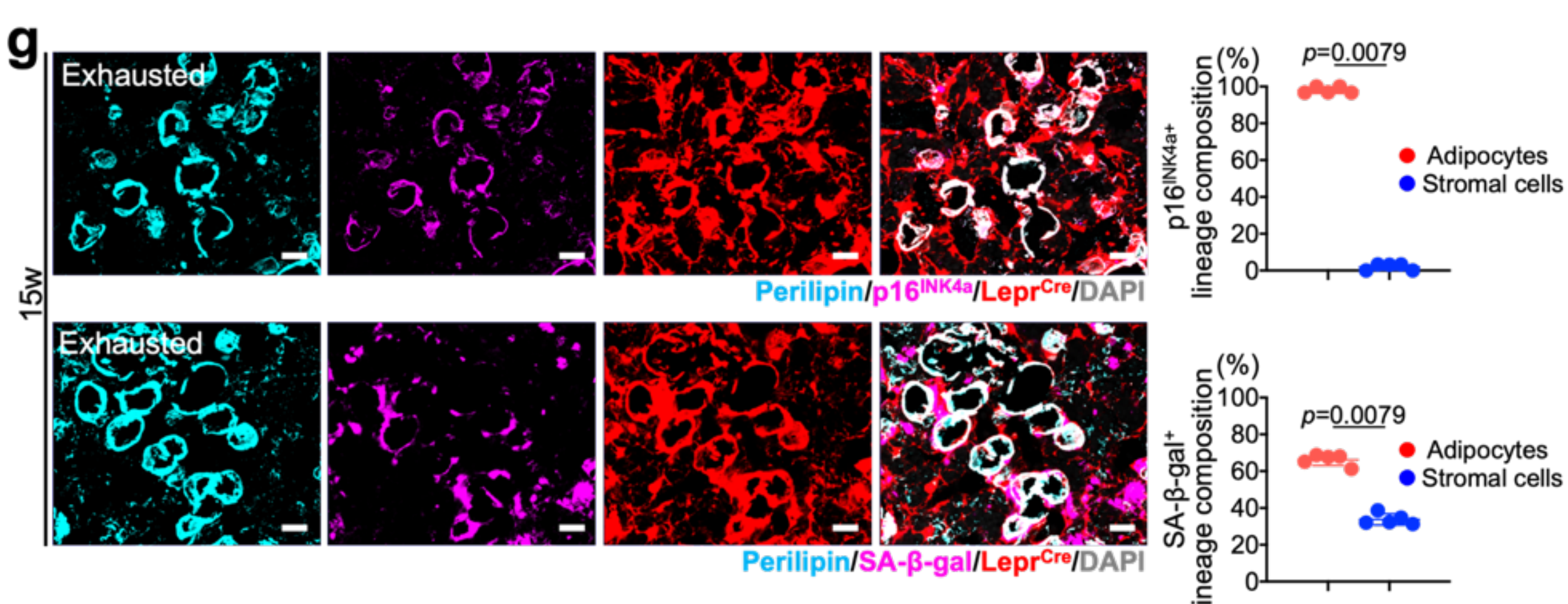

We further validated these observations using *p16-creER*; *R26R*^{tdTomato} mice. Consistent with the histological results, the majority of p16-labeled cells corresponded to adipocytes, while only a small fraction overlapped with LepR^{+} stromal cells. These data have been included as revised **Figure 2h**.

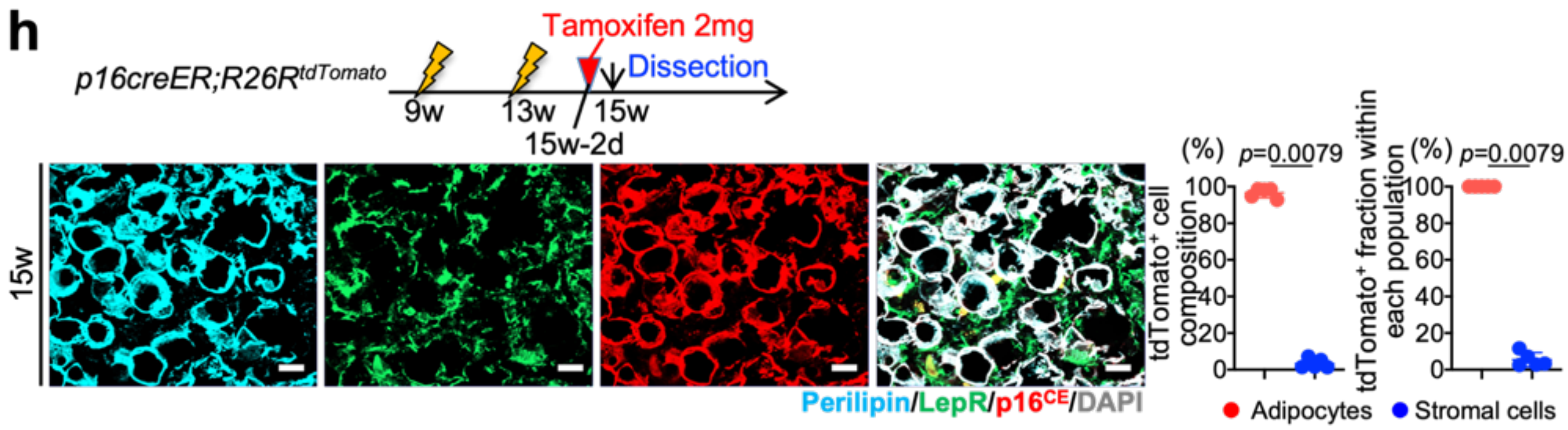

These findings indicate that although a minor subset of stromal cells may display senescence-associated features, the predominant senescent population in the exhausted bone marrow consists of adipocytes. Thus, the altered stromal phenotype after repeated injury cannot be explained primarily by canonical stromal senescence. Rather, our data support the concept of stromal exhaustion, in which stromal cells undergo functional deterioration without necessarily entering a classical senescent state. This interpretation is also compatible with the point raised by Reviewer 2 that stromal exhaustion may represent a biological state distinct from canonical cellular senescence. These findings therefore refine our conclusion by indicating that stromal dysfunction in this context reflects exhaustion rather than classical senescence.

The following sentences were added to the main text:

Line 219-233:

However, further histological analysis revealed that p16INK4a and SA-β-gal signals were predominantly detected in perilipin^{+} adipocytes rather than in LeprCre-tdTomato^{+} stromal cells (Fig. 2g). Consistently, p16-creER; R26R^{tdTomato} lineage cells labeled after the second injury showed that p16+ cells largely overlapped with adipocytes, whereas only a limited fraction overlapped with LepR^{+} stromal cells (Fig. 2h). These findings suggest that, although senescence-associated cells accumulate in the exhausted marrow environment, the exhausted phenotype of stromal cells is not simply explained by classical cellular senescence of the stromal population itself.

In summary, our findings demonstrate that exhausted bone marrow stromal cells undergo significant adipogenic differentiation ~~and increased senescence post-injury~~, leading to elevated marrow fat accumulation. ~~Moreover, cellular senescence may underlie injury-driven stromal exhaustion and potentially contribute to impaired bone regeneration and marrow homeostasis.~~ Although senescence-associated cells increased in the exhausted marrow environment, these signals were predominantly associated with adipocytes rather than stromal cells themselves. Thus, the exhausted phenotype is characterized primarily

by adipogenic conversion and loss of stromal regenerative capacity, rather than being simply explained by classical cellular senescence of stromal cells.

11. <Reviewer>

Fig 5: it is unlikely that the 10X scRNAseq assays can capture mature adipocytes (due to size and processing as discussed by authors). Cluster should be renamed into adipogenic or adipocytic in Fig.5c. Similarly, Clusters 5 and 6 might contain Osteo-CAR cells (CXCL12 low) and should be identified as such. Pie charts for differences in cluster abundance (individually) should be shown in Fig 5e.

<Response>

Thank you very much for this helpful suggestion. Following the reviewer's comments, as well as related feedback from other reviewers, we reanalyzed the single-cell RNA-seq dataset by additionally incorporating the Aged-injury group. This expanded dataset enabled us to perform a new round of sub-clustering analysis, which substantially refined the clustering structure compared with the previous version.

Based on the reviewer's suggestion, we also revised the cluster annotations. In particular, the cluster previously labeled as Adipocyte has been renamed Adipogenic cells. Although our dataset was generated using the BD Rhapsody platform rather than the 10X system, large mature adipocytes are generally difficult to capture efficiently in droplet- or microwell-based single-cell RNA-seq platforms due to their size and processing limitations. Therefore, the revised terminology more accurately reflects the likely identity of these cells. Furthermore, considering the lineage specificity of Lepr-cre–labeled cells, which include osteoblasts and osteocytes, we refined the annotation of osteogenic populations and identified Osteoblast and Osteocyte clusters as distinct cell populations, which is also consistent with our histological observations. These data have been included as revised **Figures 6a-d**.

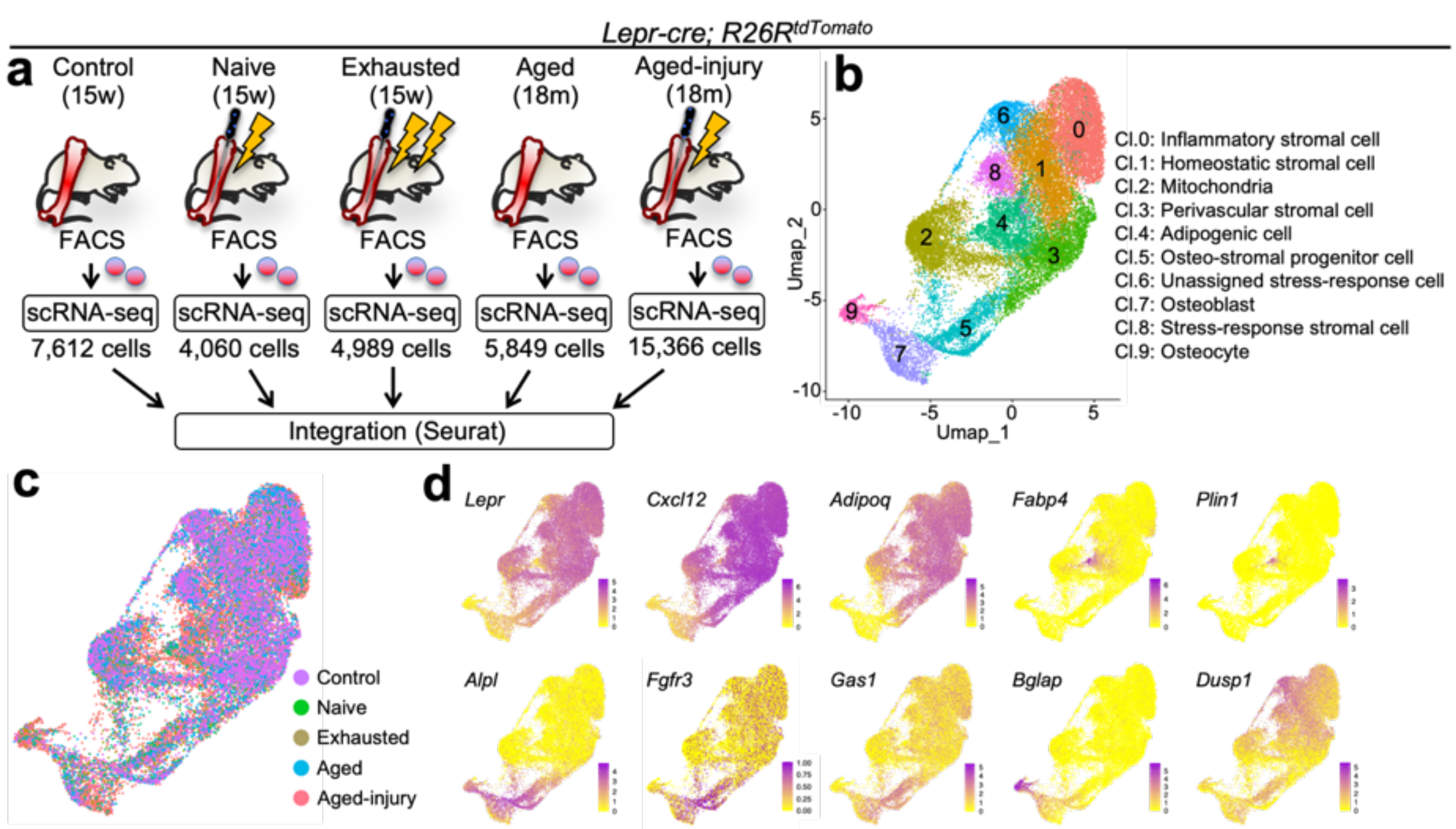

The following sentences were added to the main text:

Line 366-371:

Notably, the proportion of adipogenic cells did not substantially differ among groups in the scRNA-seq dataset. However, mature adipocytes are difficult to capture efficiently by single-cell RNA-seq because of their large size and fragility during tissue dissociation and processing. Therefore, adipocyte abundance, particularly in the exhausted group, may be underestimated in our scRNA-seq analysis despite clear histological evidence of adipocyte accumulation.

While we agree that the identification of Osteo-CAR–like populations is an important point, the cluster structure and cluster numbering changed in the revised analysis because we incorporated the Aged-injury group and repeated sub-clustering. Therefore, the reviewer's original Cluster 5/6 classification could not be directly mapped onto the revised clusters. In the revised dataset, Cxcl12 and Lepr were enriched in clusters 0, 1, 3, and 8; thus, we defined these clusters as CAR/stromal cell populations. Within these CAR/stromal clusters, we compared the relative expression of Adipo-CAR–associated markers (e.g., Cxcl12, Lepr, Adipoq), adipogenic markers (e.g., Fabp4, Plin1), and osteogenic markers (e.g., Bglap, Dmp1, Alpl). Cluster 3 showed relatively high expression of Cxcl12, Lepr, and Adipoq, suggesting an Adipo-CAR–like profile, whereas cluster 8 showed elevated expression of osteogenic markers such as Bglap and Dmp1, consistent with an Osteo-CAR–like profile. However, these identities were not mutually exclusive. Cluster 3 also expressed the osteogenic marker Alpl, and cluster 8 expressed adipogenic markers such as Fabp4 and Plin1. Because the revised dataset contains many injury-responsive stromal cells rather than steady-state CAR cells, these populations likely represent transitional or activated stromal states with mixed lineage features. We therefore avoided explicitly assigning these clusters to discrete Adipo-CAR or Osteo-CAR categories in the manuscript. These data have not been included in the revised manuscript, but are shown **below**.

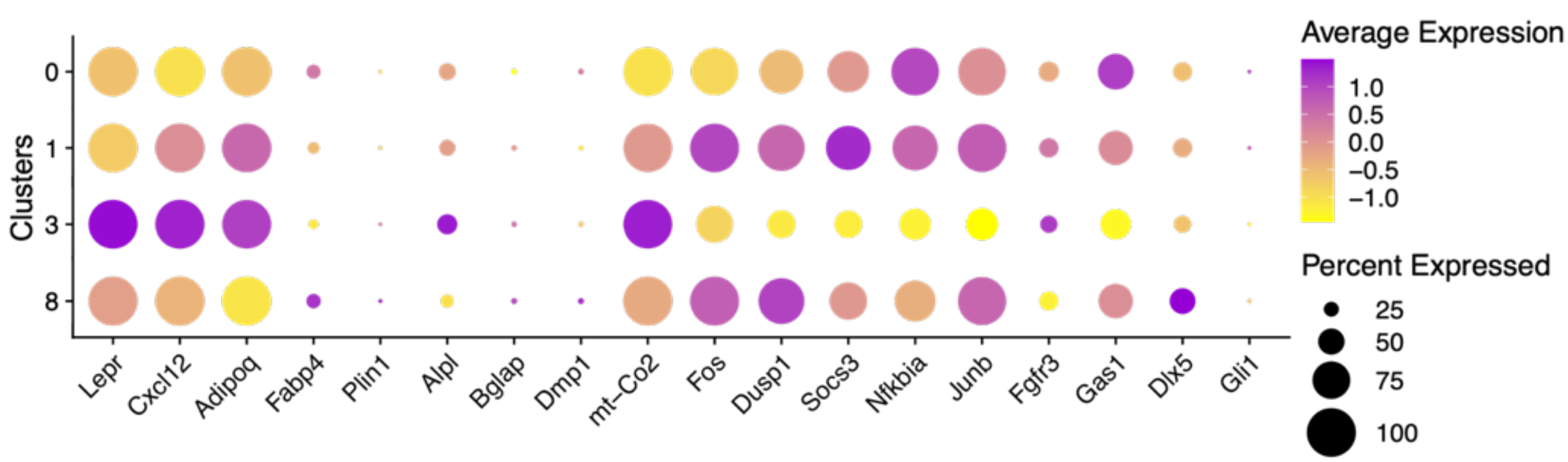

Finally, we appreciate the reviewer's helpful suggestion to summarize cluster abundance across conditions. We generated pie charts to provide an intuitive overview of the compositional differences among groups. Because these plots are descriptive and not intended for formal statistical comparison, we have

provided them below for the reviewer’s reference rather than including them in the revised figures. The data are shown **below** for reference.

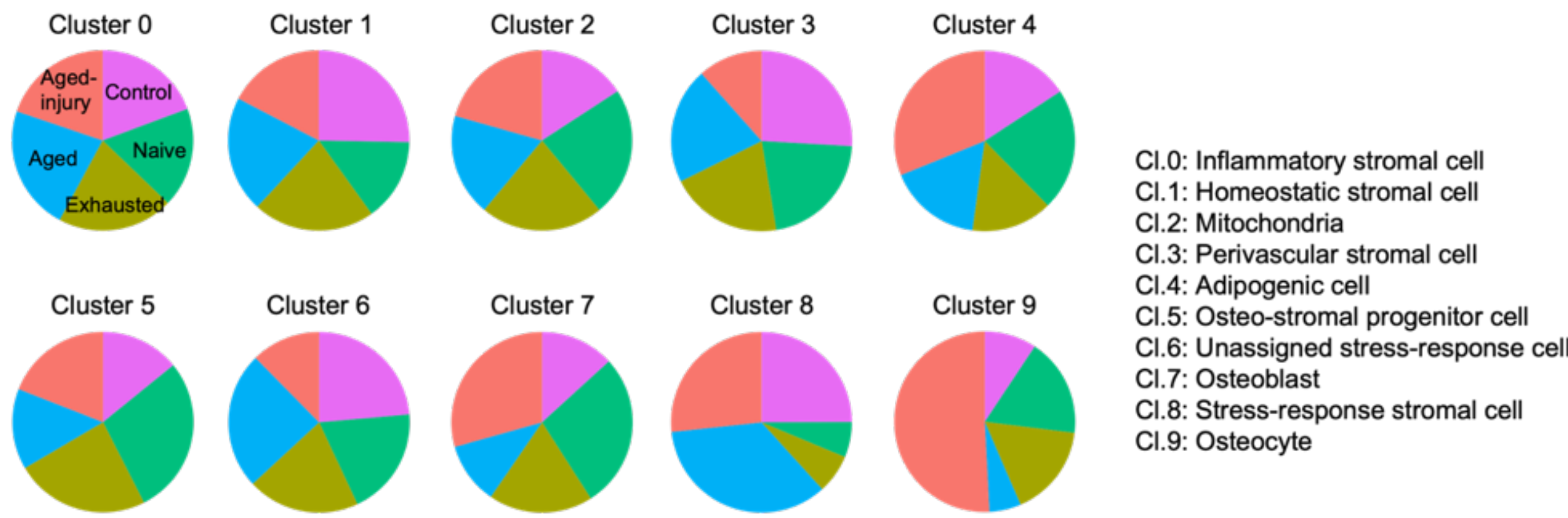

12. <Reviewer>

RNA velocity/Cell Rank data is speculative and does not account for different lineages within entire cell pool analyzed. What is the “Skeletal Progenitor” cluster – shouldn’t stromal cells also be considered skeletal progenitors if they generate osteo cells? Similarly, one could interpret results as if only periosteal cell contribute to adipogenic cells which does not align with the authors conclusions.

<Response>

Thank you very much for these important comments. As noted by Reviewers 2 and 3, trajectory analyses such as RNA velocity or CellRank can be informative but are inherently speculative. In the original figure, an arrow appeared to extend from the periosteal cluster toward a portion of the adipogenic cluster, although it did not point toward the peak of the adipogenic cluster. From a biological perspective, periosteal cells and bone marrow adipogenic cells are spatially separated by the cortical bone in the adult stage, making it unlikely that one population directly gives rise to the other. Therefore, we consider this trajectory to be biologically implausible and not meaningful. However, we agree that such a graphical representation could lead to misinterpretation of lineage relationships. Since this trajectory analysis is not central to the main focus of the manuscript, we have removed it from the revised version to avoid potential confusion.

In addition, as this study focuses specifically on stromal populations within the bone marrow compartment, we excluded the periosteal cluster during the sub-clustering analysis in the revised manuscript. Furthermore, we revised the cluster annotation. The cluster previously labeled as “skeletal progenitor” has been renamed “osteo-stromal progenitor” to better reflect its molecular characteristics and potential lineage outputs. This population is characterized by high expression of *Fgfr3*, *Gas1*, and *Alpl*, and likely represents skeletal progenitor cells capable of giving rise to both osteogenic and stromal lineages (including CAR cells), as reported in previous studies (Matsushita et al., Nat Commun 2023; Kondo et al., Commun Biol 2026). These data have been included as revised **Figures 6b,d**.

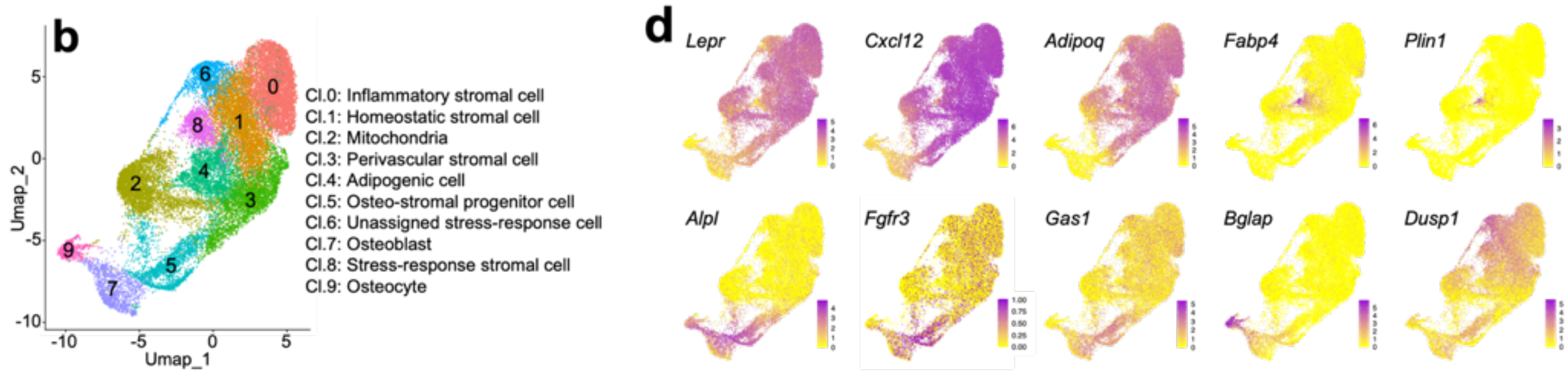

13. <Reviewer>

Fig 5i: Please include all four groups in dot plot analysis for transparency. What about changes in hematopoiesis support which is crucial for regeneration after ablation procedures? The common marker expression between exhaustion and aged groups are also quite limited and selective.

<Response>

Thank you very much for this helpful comment. We agree that transparency is important when comparing the molecular features of different groups. In the main figure, we focused the dot plot on the most relevant comparisons to highlight the contrast between aged and exhausted stroma while maintaining readability. In the revised manuscript, the number of conditions has increased from four to five, and including all groups in a single main figure panel would make the visualization more complex. To address the reviewer's concern and ensure transparency, we have therefore provided the complete five-group dot plot **below**, while retaining the simplified version in the main figure for clarity.

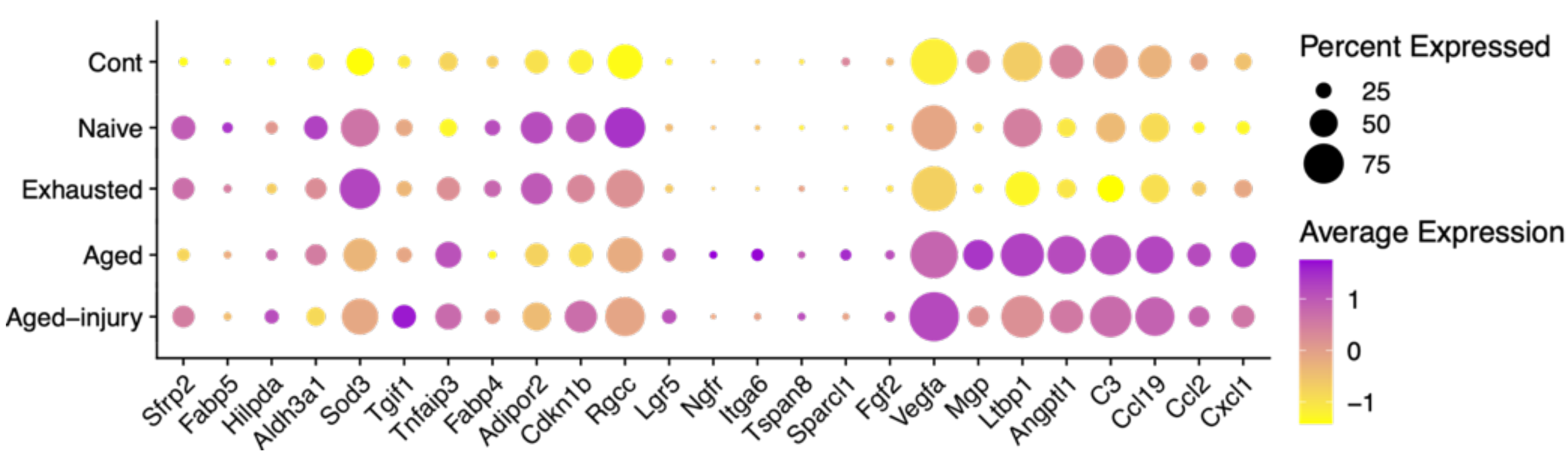

We also appreciate the reviewer's important point regarding hematopoietic support, which is highly relevant to regeneration after marrow ablation. To address this point, we examined representative hematopoietic niche/support genes and provide the results **below**. Although our goal was not to make definitive conclusions regarding the functional strength of hematopoietic support across groups, the expression patterns of niche-related genes differed substantially among conditions. Notably, exhausted and aged stroma showed clearly distinct profiles, further supporting the idea that stromal exhaustion represents a distinct microenvironmental state rather than a simple extension of aging. At the same time, we acknowledge that gene expression alone is not sufficient to determine the functional impact of these stromal

populations on hematopoiesis. Therefore, we have limited our interpretation to the transcriptional differences in niche-related genes and have avoided drawing conclusions about functional hematopoietic support in the revised manuscript.

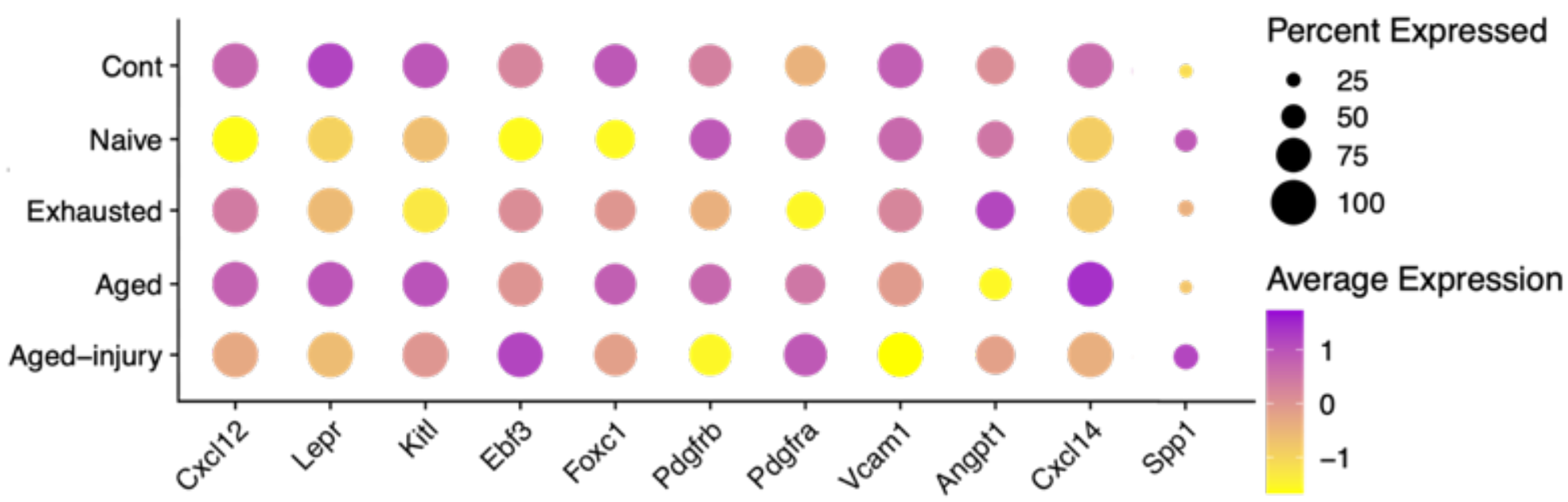

Regarding the relatively limited overlap of marker expression between the exhausted and aged groups, we consider this observation to be an important feature of the present study. Rather than indicating a limitation of the analysis, the limited and selective overlap suggests that the exhausted state is not simply an extension of aging, but instead represents a molecularly distinct stromal state induced by repeated injury. To ensure that this conclusion was not driven by selective marker choice, we performed unbiased DEG-based analyses. Specifically, we conducted Metascape enrichment analyses using all 33 genes commonly upregulated in both exhausted and aged stroma, as well as the top 100 DEGs specifically upregulated in exhausted stroma or aged stroma. These analyses consistently revealed marked differences between the two conditions. The results are shown **below**.

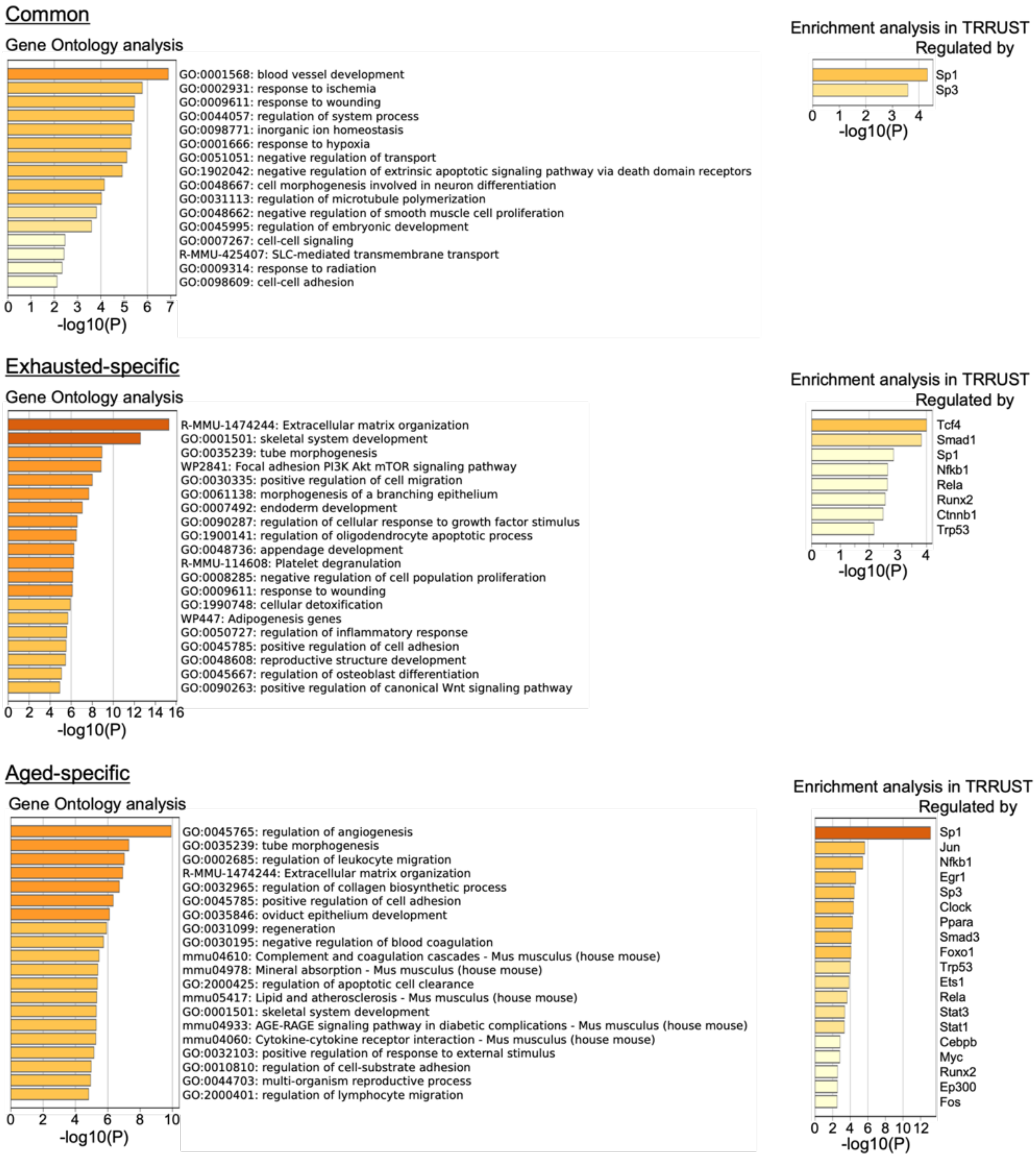

Compared with control stroma, both aged and exhausted stroma shared enrichment of pathways related to vascular-associated processes, wound response, hypoxia, and cell adhesion, suggesting a shift from a homeostatic stromal state toward a more reactive state. However, the biological nature of this remodeling appeared distinct between the two conditions. Aged stroma, derived from uninjured physiologically aged marrow, showed enrichment of pathways related to angiogenesis, leukocyte migration, cytokine-related processes, coagulation/complement pathways, and matrix remodeling, consistent with a chronically remodeled but relatively regulated stromal state. In contrast, exhausted stroma, induced by

repeated injury, was characterized by strong enrichment of extracellular matrix organization, wound response, cell migration, inflammatory regulation, and adipogenic signatures, suggesting a maladaptively activated failed-repair state rather than a simple extension of physiological aging. Overall, these results support the concept that stromal exhaustion represents a biologically distinct injury-induced state, rather than merely accelerated aging of the bone marrow niche.

These revisions further clarify the distinct molecular identity of exhausted stroma within the regenerating bone marrow microenvironment.

The following sentences were added to the main text:

<u>*Line 382-388:*</u>

Although both populations share a subset of reactive stromal features, including genes associated with wound response, stress response, and extracellular matrix remodeling, exhausted stroma was uniquely characterized by enhanced adipogenic programming (Fabp4, Adipor2) and cell cycle regulation/arrest-associated genes (Cdkn1b, Rgcc), consistent with functional exhaustion. In contrast, aged stromal cells retained genes associated with stromal maintenance and niche-supportive functions, including Lgr5, Itga6, Vegfa, Fgf2, Sparcl1, and Ltbp1 (Fig. 7h).

14. <Reviewer>

Fig.6: Again, Control group should be included here. Are these changes also seen in exhausted compared to untreated control? In 6g authors show control group data, which seems to resemble exhausted group. If these differences are just seen when compared to healthy, this could also imply that the one time ablation triggers a strong response, but not necessarily a "better" response. Would the same be observed if second injury/ablation was done 2-4 weeks later?

<Response>

Thank you very much for this important comment. First, we added the Control group to the dot plots in **Figures 8d-f** to allow direct comparison across groups. In addition, as suggested by Reviewer 2, we re-evaluated the marker selection for inflammation, stress response, and adipogenic/lipid metabolism pathways to more accurately reflect the underlying biological processes.

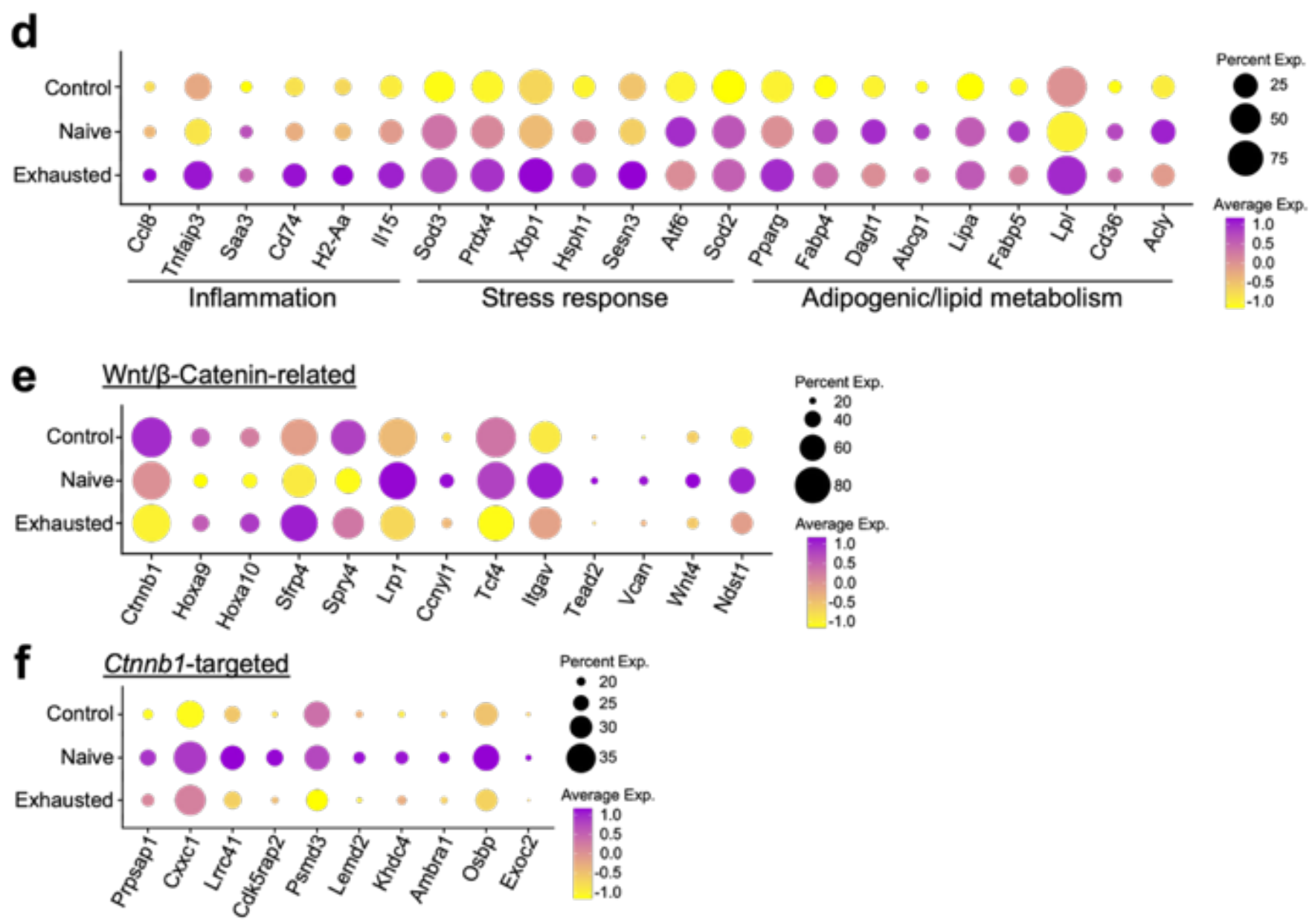

In this study, the key point is that naive (formerly healthy) stromal cells show a robust osteogenic regenerative response after the first injury. We do not define this response as "better" simply because it is stronger; rather, we interpret it as regenerative because it is associated with osteogenic differentiation and bone formation. In contrast, after repeated injury, this osteogenic regenerative program is not properly reactivated, and bone formation is markedly impaired. We therefore define stromal exhaustion as the loss of injury-induced regenerative competence.

Genes related to inflammation, stress response, and adipogenic/lipid metabolism were expressed at low levels in the Control group, but were upregulated after injury in both the Naive and Exhausted groups. Notably, their expression was particularly strongly increased in the Exhausted group. Together, these results indicate that stromal cells shift from a homeostatic state (Control) toward an inflammatory, stress-associated, and metabolically imbalanced state after injury, with the strongest alteration observed after repeated injury.

In contrast, Wnt/β-catenin signaling-related programs were specifically activated after the first injury (Naive group), reflecting the regenerative response of stromal cells. This activation was absent after repeated injury. Therefore, the observation that the Exhausted group shows a gene expression pattern similar to that of the untreated Control group for some Wnt/β-catenin–related genes does not contradict our interpretation. Rather, it is consistent with our interpretation that stromal cells in the exhausted state fail to mount an appropriate regenerative response after repeated injury. Although *Ctnnb1* mRNA showed relatively high expression in the Control group, activation of the Wnt/β-catenin pathway depends primarily on β-catenin stabilization at the protein level, rather than *Ctnnb1* transcript abundance alone. Consistently, β-catenin protein expression was increased only in the Naive stroma after a single injury (**Figure 8g**).

Importantly, in our experimental design, the second injury was performed four weeks after the first ablation surgery, directly testing the response to repeated damage. Furthermore, even when the second injury was performed 12 weeks after the first ablation surgery, stromal cells still failed to have a robust

osteogenic response and instead showed a shift toward adipogenic differentiation, consistent with the exhausted phenotype. These data have been included as revised **Figure 3d-h**.

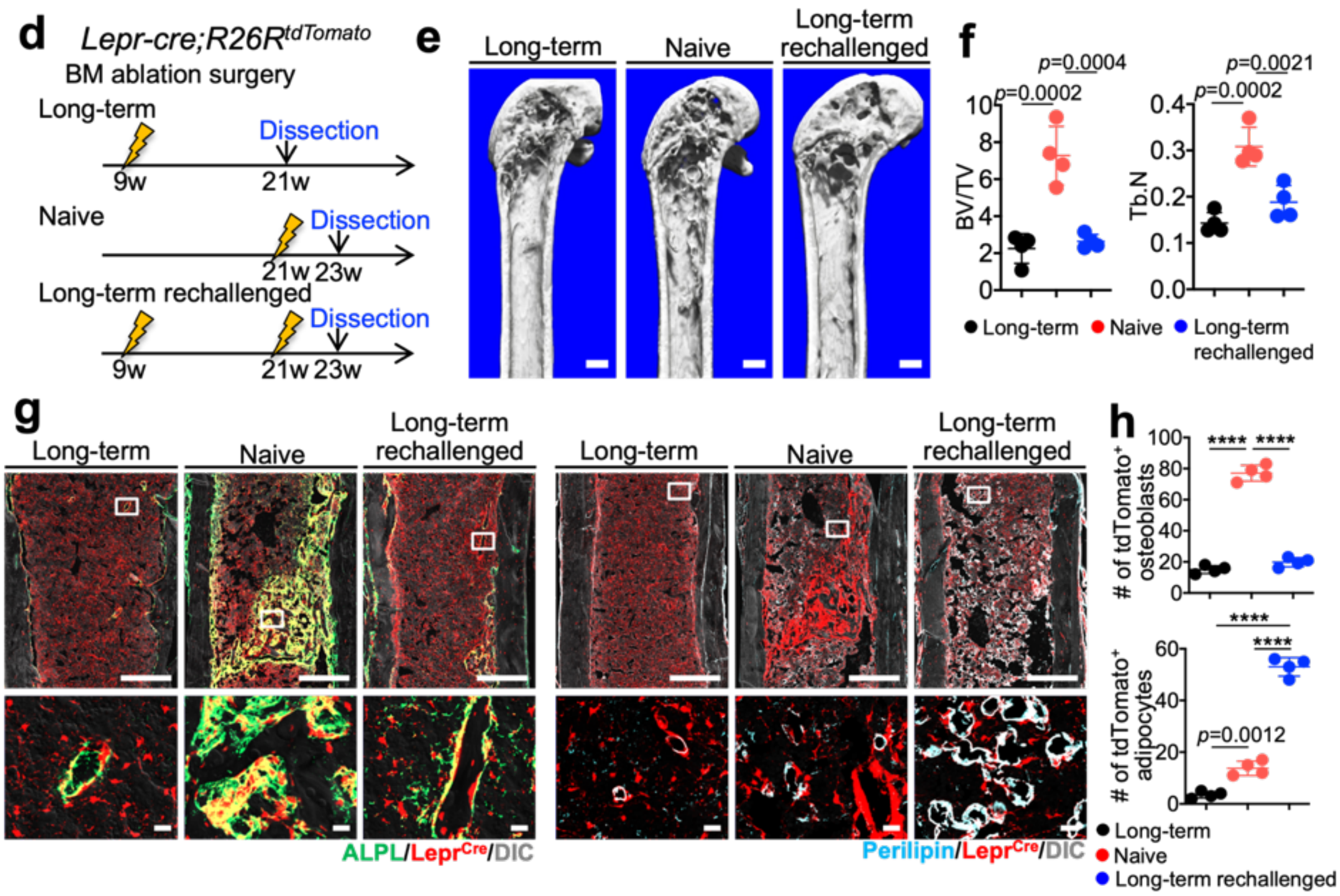

Together, these findings indicate that stromal exhaustion reflects a loss of regenerative responsiveness after repeated injury, rather than simply a difference between injured and naive states.

The following sentences were added to the main text:

<u>*Line 245-265:*</u>

We further examined whether the four-week interval after the first injury was insufficient for recovery and whether stromal exhaustion reflected a transient delay rather than a sustained functional alteration. To address this, we performed a long-term rechallenge experiment, with a 12-week interval between the first and second bone marrow ablation surgeries (Fig. 3d). Micro-CT analysis revealed robust trabecular bone regeneration in the naive group, whereas the long-term rechallenged group showed markedly reduced trabecular bone formation after the second injury (Fig. 3e). Quantitative analysis confirmed significantly lower BV/TV and trabecular number in the long-term rechallenged group than in the naive group (Fig. 3f). In the long-term group, analyzed 12 weeks after a single injury, the marrow cavity had largely returned to a histologically homeostatic state, with limited osteoblast or adipocyte accumulation (Fig. 3g). In contrast, in the long-term rechallenged group, Lepr-cre lineage cells showed a markedly reduced osteogenic response and enhanced adipogenic differentiation (Fig. 3g,h).

These findings indicate that stromal exhaustion is not merely a transient delay in recovery, but rather reflects a persistent functional alteration of injury-experienced marrow stromal cells.

15. <Reviewer>

Furthermore in Figure 6, the average expression is displayed as one color (not range as indicated in scale bar), which might be due to the fact that there are just two groups compared (on or off) making it hard to gauge how different expression differences are.

<Response>

Thank you very much for this helpful comment. We agree that the previous color scale made it difficult to appreciate differences in average expression. To improve visualization and interpretability, we changed the color scale from the blue-gray scale to a violet-yellow scale with a broader dynamic range. This revision makes the relative differences in average expression across groups easier to assess.

16. <Reviewer>

Fig.7: Given the widely known role of Wnt signaling in osteogenesis it is not surprising or new that ablation of Ctnnb1 would have the observed effect. The connection to potential stromal exhaustion remains weak. This model also does not allow distinction between changes at stromal vs more mature osteoblast level in response to Ctnnb1 ablation. There is also no data inquiring which distinct source (location) of stromal progenitors are affected. LepR expression is extremely broad, and the investigation of more defined subsets (multi marker) would have provided more novelty.

<Response>

Thank you very much for these important comments. As the reviewer noted, the role of Wnt/β-catenin signaling in osteogenesis is well established. However, the key finding of our study is not simply that Wnt signaling promotes bone formation, but that stromal exhaustion is characterized by a failure to re-activate Wnt/β-catenin signaling in response to injury, as revealed by our unbiased scRNA-seq analysis. We believe that this impaired reactivation represents a fundamental feature of the exhausted stromal state.

To further test the functional relevance of this observation, we performed rescue experiments by activating Wnt/β-catenin signaling using CHIR-99021, a GSK-3 inhibitor. Administration of CHIR-99021 around the second bone marrow ablation restored osteogenesis and reduced adipogenesis in the bone marrow. These results support the idea that impaired Wnt/β-catenin activation is mechanistically linked to stromal exhaustion. These data have been included as revised **Figure 10a-d**.

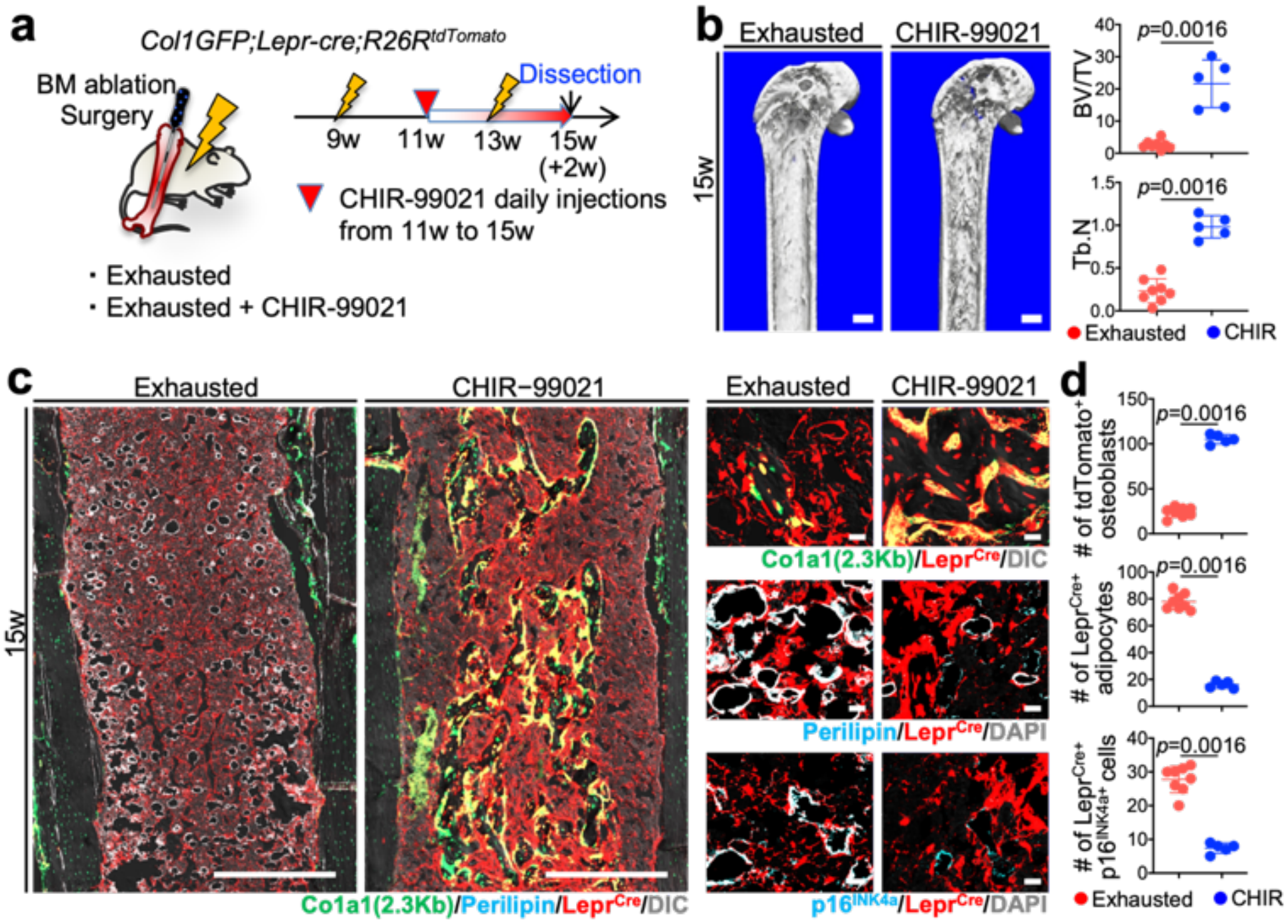

Furthermore, to genetically test this mechanism, we utilized *Lepr-cre*; *Ctnnb*$^{lox(ex3)/+}$; *R26R*^{tdTomato} mice, in which β-catenin is constitutively stabilized in Lepr-cre$^+$ stromal lineage cells. When these mice underwent two rounds of bone marrow ablation surgery, the adipogenic conversion of stromal cells that normally accompanies stromal exhaustion was markedly suppressed, and stromal cells retained their osteogenic potential. These data have been included as revised **Figures 10e-h**.

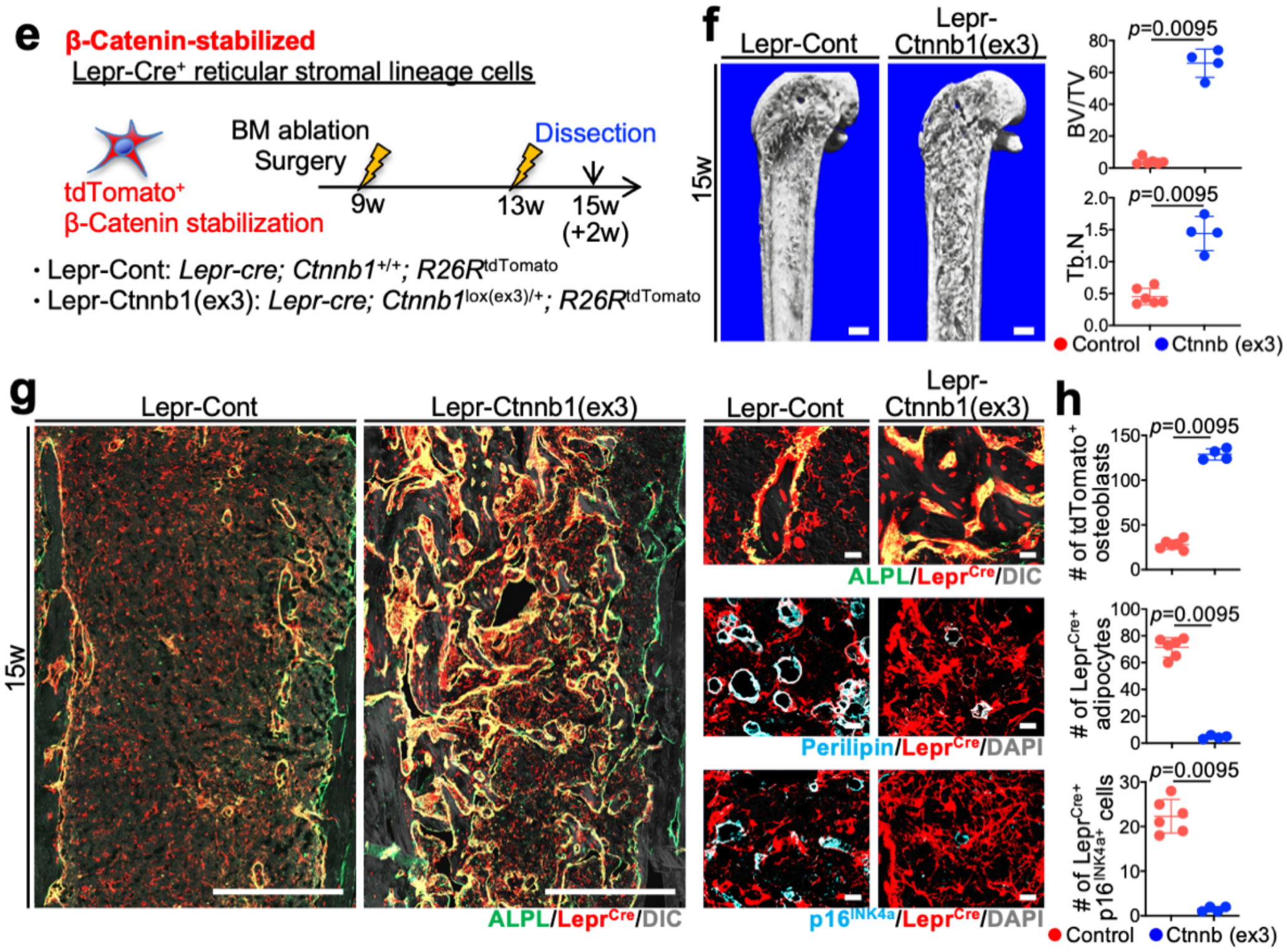

Together with the pharmacological rescue experiments using CHIR-99021, these findings indicate that the inability to re-activate Wnt/β-catenin signaling after repeated injury represents a central mechanism underlying stromal exhaustion. In other words, Wnt/β-catenin signaling is normally induced after the first injury to drive regenerative osteogenesis, but this injury-responsive activation fails after repeated injury. Importantly, restoring Wnt/β-catenin signaling was sufficient to suppress the exhaustion-associated adipogenic shift, demonstrating that impaired Wnt reactivation is a key driver of the stromal exhaustion phenotype.

The following sentences have been added to the main text:

Line 113-115

Conversely, pharmacological or genetic activation of Wnt/β-catenin signaling restores osteogenic regeneration and suppresses adipogenic conversion, supporting its causal role in preventing stromal exhaustion.

Line 474-493

To determine whether activation of Wnt/β-catenin signaling could reverse the exhausted phenotype, we next performed pharmacological and genetic rescue experiments. First, we administered daily injections of CHIR-99021, a GSK-3 inhibitor that activates Wnt/β-catenin signaling, from 11 to 15 weeks of age, during the period of the second bone marrow ablation surgery in the repeated-injury model (Fig. 10a). Micro-CT analysis revealed that CHIR-99021 treatment significantly increased trabecular bone regeneration after repeated injury, as shown by increased BV/TV and Tb.N compared with untreated exhausted mice (Fig. 10b). Histological analysis showed that CHIR-99021 restored the contribution of $Lepr^{Cre+}$ stromal lineage cells to $Col1a1\text{-}GFP^{+}$ osteoblasts while reducing the accumulation of $Lepr^{Cre+}$ adipocytes and p16INK4a-positive cells in the bone marrow (Fig. 10c,d). These findings indicate that pharmacological activation of Wnt/β-catenin signaling partially rescues the impaired osteogenic response and suppresses adipogenic conversion in exhausted stroma.

We further examined whether genetic activation of β-catenin in $Lepr^{+}$ stromal lineage cells could rescue stromal exhaustion. For this purpose, we used Lepr-cre; $Ctnnb1^{lox(ex3)/+}$; $R26R^{tdTomato}$ (Lepr-Ctnnb1(ex3)) mice, in which β-catenin is constitutively stabilized in $Lepr^{Cre+}$ cells (Fig. 10e). Following repeated injury, Lepr-Ctnnb1(ex3) mice showed increased bone mass compared with exhausted controls (Fig. 10f). Histological analysis further revealed that β-catenin stabilization preserved osteogenic differentiation of $Lepr^{Cre+}$ stromal lineage cells and markedly suppressed their adipogenic conversion, with a concomitant reduction in $p16^{INK4a}$ expression in the exhausted marrow environment (Fig. 10g,h).

Line 498-502

Conversely, pharmacological activation or genetic stabilization of β-catenin restored osteogenesis and suppressed adipogenic conversion after repeated injury. Together, these loss- and gain-of-function approaches identify impaired Wnt/β-catenin reactivation as a key mechanism underlying stromal exhaustion.

Line 586-594

Therapeutically, ~~our findings highlight actionable strategies to reverse stromal exhaustion, with the Wnt/β-catenin pathway emerging as a possible target~~ our rescue experiments identify Wnt/β-catenin signaling as a validated target for reversing key features of stromal exhaustion. Suppression of this pathway drives adipogenic bias and loss of osteogenic function in Lepr+ stromal cells, whereas pharmacological activation with CHIR-99021 or genetic stabilization of β-catenin restores osteogenic regeneration and suppresses adipogenic conversion after repeated injury. ~~suggesting~~ These findings suggest that restoring canonical Wnt signaling may ~~rejuvenate the niche~~ re-establish regenerative stromal function within the exhausted marrow niche. Potential translational approaches include sclerostin-neutralizing antibodies (e.g., romosozumab), already approved for osteoporosis, pharmacological activation via GSK3β inhibitors, or development of Wnt ligand mimetics.

Line 602-604

Importantly, the reversibility of key exhausted features through Wnt/β-catenin activation suggests that stromal exhaustion is a therapeutically targetable form of regenerative failure.

We also agree with the reviewer that identifying which mesenchymal populations are affected by Wnt/β-catenin signaling is important. Previous studies have shown that Lepr-cre$^+$ stromal lineage cells contribute relatively little to osteoblasts and osteocytes in young-adult bone, and their contribution gradually increases with age (Zhou et al., Cell Stem Cell 2014). Consistent with this model, although we did not observe a clear bone phenotype in young mice, in the revised study, we additionally analyzed *Lepr-cre*; *Ctnnb1*$^{fl/fl}$ mice at 6 months of age and observed a reduction in bone mass. This result suggests that Wnt/β-catenin signaling in Lepr-cre$^+$ stromal lineage cells becomes increasingly important for bone homeostasis as their contribution to the osteoblast lineage increases with age. These data have been included as revised **Figure 9b**.

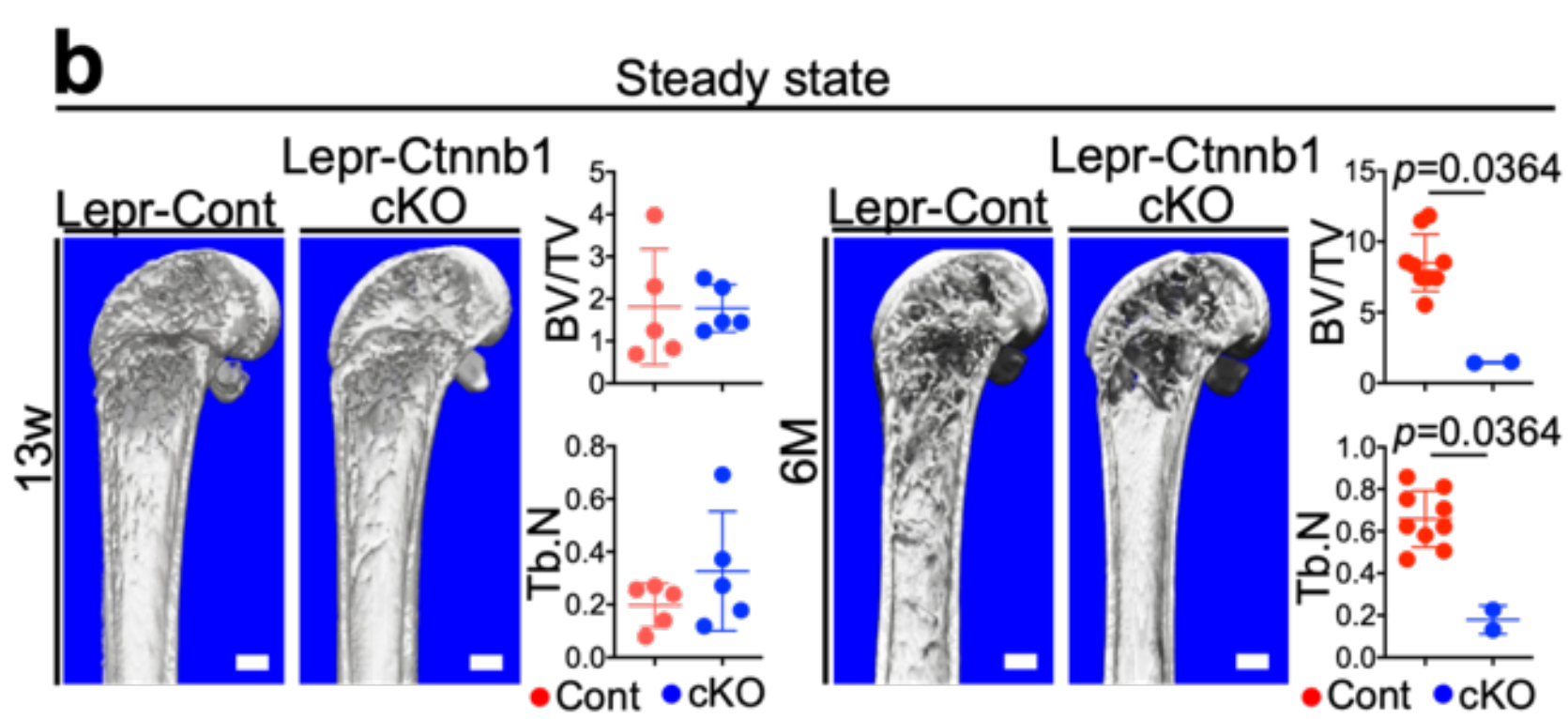

In addition, our previous work demonstrated that Cxcl12$^+$ reticular stromal cells can dedifferentiate into skeletal stem cell–like cells during bone regeneration (Matsushita et al., Nature Communications 2020), in which Wnt/β-catenin signaling becomes activated and contributes to osteogenic regeneration. This suggests that Wnt signaling in Lepr$^+$Cxcl12$^+$ stromal cells may play a particularly important role during injury-induced regenerative activation, rather than during steady-state bone maintenance. Furthermore, our other

study showed that Fgfr3⁺ endosteal skeletal stem cells and their downstream Gas1⁺ osteo-stromal progenitors give rise to Lepr⁺Cxcl12⁺ reticular stromal cells and osteoblasts during bone growth (Matsushita et al., Nature Communications 2023). Consistent with this hierarchical model, we additionally performed *Ctnnb1* deletion in upstream skeletal stem and progenitor populations using *Fgfr3-creER* and *Gas1-creER* mouse lines. This resulted in significant bone loss, indicating that Wnt/β-catenin signaling plays an essential role in these earlier skeletal lineages in young adults. Because these experiments address the broader skeletal lineage hierarchy rather than the central injury-induced exhaustion model, we provide these data **below** for the reviewer's reference rather than incorporating them into the revised manuscript.

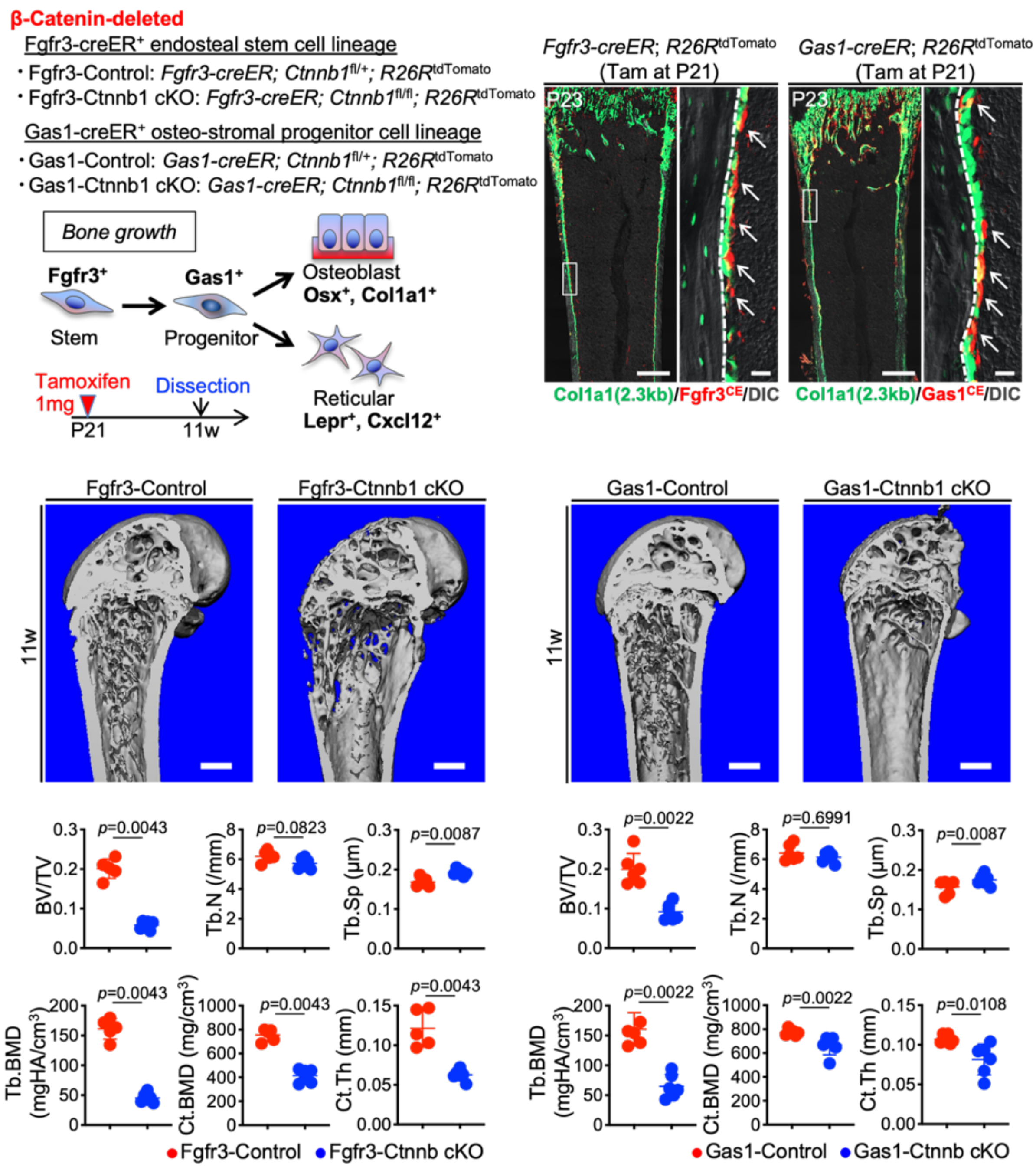

Taken together, these results indicate that Wnt/β-catenin signaling regulates osteogenic potential across multiple levels of the skeletal lineage hierarchy, from upstream endosteal stem and progenitor cells to injury-responsive stromal populations.

The following sentences have been added to the main text:

Line 454-459:

Micro-CT analysis of femurs revealed no significant differences in trabecular BV/TV or Tb.N between control and Lepr-Ctnnb1 cKO mice under uninjured conditions *at 13 weeks of age (Fig. 9b). In contrast, Lepr-Ctnnb1 cKO mice showed reduced trabecular bone mass at 6 months of age (Fig. 9b), suggesting that β-catenin signaling in Lepr^{+} stromal lineage cells becomes increasingly important for bone homeostasis as their contribution to the osteoblast lineage increases with age.*

17. <Reviewer>

It is unclear where the "metabolic imbalance" in the title is derived from.

<Response>

Thank you very much for this helpful comment. We agree that the phrase "through metabolic imbalance" may have overemphasized one aspect of the phenotype and did not fully reflect the broader concept of stromal exhaustion. We have therefore removed "through metabolic imbalance" from the title.
